# The role of healthy lifestyle in the association between hepatic fibro-inflammation and steatosis and brain aging—a cross-sectional study

**DOI:** 10.3389/fnagi.2026.1801577

**Published:** 2026-03-16

**Authors:** Jikang Shi, Zhuoshuai Liang, Wenhui Gao, Huizhen Jin, Yinglin Du

**Affiliations:** 1 Department of Clinical Nutrition, Peking University Shenzhen Hospital, Shenzhen PKU-HKUST Medical Center, Shenzhen, China; 2 Department of Epidemiology and Biostatistics, School of Public Health of Jilin University, Changchun, China; 3 Shandong Center for Disease Control and Prevention, Jinan, Shandong, China

**Keywords:** brain age prediction, hepatic fibro-inflammation, proton density fat fraction, steatotic liver disease, T1 mapping imaging

## Abstract

**Background:**

This study investigated the association between liver fat, liver fibro-inflammation, and brain age, and assessed whether modifiable lifestyle factors modified the associations between liver markers and brain age.

**Methods:**

A total of 19,566 adults free from dementia and other neurological disorders were included from the UK Biobank. Liver fat and fibro-inflammation were quantified using proton density fat fraction (PDFF) and iron-corrected T1 mapping (cT1) derived from liver MRI scans. Brain age was estimated using a machine learning model based on 1,079 brain MRI phenotypes, and brain-predicted age difference (brain-PAD) was calculated.

**Results:**

Among participants, 4,634 (23.7%) had significant steatotic liver disease (PDFF ≥ 5.5%), and 938 (4.8%) had significant fibro-inflammation (cT1 ≥ 800 ms). Both significant liver fat accumulation (β = 0.4, 95% CI: 0.21–0.59) and fibro-inflammation (β = 1.09, 95% CI: 0.74–1.44) were associated with increased brain-PAD. Joint exposure analysis showed that the association between liver fat/fibro-inflammation and increased brain-PAD was attenuated among individuals with healthy lifestyle choices.

**Conclusion:**

Liver fat accumulation and fibro-inflammation were associated with higher brain-PAD, with fibro-inflammation playing a key role. These associations may be attenuated in individuals with optimal lifestyle behaviors, suggesting a potentially modifiable target for intervention.

## Introduction

The incidence of chronic liver disease (CLD) associated with steatotic liver disease (SLD) has been increasing over the last decade (Teng et al., 2023). SLD is prevalent in the asymptomatic population, particularly among obese, diabetic, and hyperlipidemic patients, and can progress to liver inflammation, fibrosis, cirrhosis, and/or hepatocellular carcinoma (Demir et al., 2023). Recent studies have demonstrated that not only the presence of SLD, but also the accumulation of fat in the liver, is associated with remodeling of brain and heart structures (Arold et al., 2024; Qu et al., 2023).

The brain’s role in energy and glucose metabolism is pivotal, and its susceptibility to injury shares risk factors and mechanisms with liver health, highlighting the growing interest in the interplay between the liver and brain, known as the liver-brain axis. Several magnetic resonance imaging (MRI) studies of the brain have demonstrated that SLD is associated with brain atrophy (Gurholt et al., 2021) and white matter hyperintensity (WMH) (McCracken et al., 2022), while the relationship between SLD, cognitive decline, and dementia remains a topic of controversy (Huang et al., 2023; Wang et al., 2024). These inconsistencies may arise from the pathological heterogeneity of SLD. Steatohepatitis, characterized by fibro-inflammation, is associated with a more severe progression of disease compared to isolated hepatic steatosis (Sherry et al., 2023). Notably, inflammatory processes mediate the connection between liver fat and brain health (Cardamone et al., 2024), and liver inflammation induced by SLD can activate brain microglial cells and inflammatory cytokines, ultimately leading to neurodegeneration (Kim et al., 2016). Therefore, liver fibro-inflammation, rather than liver fat, may serve as the primary driver of brain damage.

Machine learning methods present a significant opportunity to estimate brain age using brain MRI data. In contrast to traditional structural neuroimaging assessments, such as regional volume and cortical thickness, brain age estimation can capture intricate patterns of structural variations and interactions among brain regions (Huang et al., 2024). The difference between the predicted brain age and chronological age is referred to as the ‘brain-Predicted Age Difference (brain-PAD)’, which serves as a marker of overall brain health. Brain-PAD reflects neuroanatomical abnormalities and is associated with an increased risk of mortality, cognitive decline, and dementia (Cole et al., 2019). Furthermore, brain-PAD can aid in the early detection of brain diseases and support differential diagnosis, prognosis, and treatment selection (Baecker et al., 2021). A recent study documented that non-alcoholic fatty liver disease (NAFLD) is linked to approximately 4.2 years of brain aging (Weinstein et al., 2018). However, considering the heterogeneity of CLD populations, environmental factors (e.g., cardiometabolic burden and lifestyle behaviors) and genetic predisposition may influence the association of SLD and fibro-inflammation with brain-PAD. Various modifiable lifestyle behaviors, such as engaging in regular physical activity and avoiding smoking and alcohol abuse, have been linked to decelerated brain aging (Cole, 2020; Franz et al., 2021). Nonetheless, it remains uncertain whether a healthy lifestyle can mitigate the adverse effects of SLD or fibro-inflammation. Importantly, it is also unclear whether liver fibro-inflammation acts as a more critical driver of brain aging than liver fat accumulation.

To address these gaps, we conducted a comprehensive investigation into the relationship between liver fat, fibro-inflammation, and brain aging, utilizing detailed neuroimaging and liver imaging data from a large population-based dataset provided by the UK Biobank. We characterized liver fat content and fibro-inflammation using iron-corrected T1 mapping (cT1) and proton density fat fraction (PDFF), which serve as reliable surrogate markers for hepatic fibro-inflammation and steatosis, respectively(Alkhouri et al., 2025; Gao et al., 2026; Liang et al., 2025). Additionally, we developed a machine learning model to estimate brain age based on multiple neuroimaging modalities. Our specific objectives were to: (1) examine the driving roles of SLD and fibro-inflammation in brain aging; (2) explore whether sex, genetic predisposition, and cardiometabolic burden modify these associations; and (3) assess whether the associations are more pronounced in males and in individuals with a high cardiometabolic burden, but attenuated in those with healthy lifestyle choices.

## Materials and methods

UK Biobank is a longitudinal cohort study with baseline assessments of more than 500,000 participants aged 40 to 70 years between 2006 and 2010. The study collected a wealth of information, including physical measurements, financial status, social activity, and follow-up, details of which are available online. A subsample of 44,464 individuals underwent brain MRI scans approximately 9 years after baseline (between 2014 and 2020). The analysis was restricted to 37,458 participants who had complete information on all available brain imaging-derived phenotypes (IDPs) (details for the missing values for each IDP can be found in Supplementary Table 1). We subsequently excluded 1,084 participants with chronic neurological disorders (including dementia) at the time of the MRI scan (Supplementary Table 2 for details), as well as 7,181 with missing PDFF, 5,238 with missing cT1, and 4,389 with missing covariates, resulting in a final sample of 19,566 participants (Supplementary Figure 1). The study received ethical approval from the North West Multicenter Research Ethics Committee, and all participants provided written informed consent.

### Acquisition of liver imaging-derived metrics

Liver MRI scans were performed on a 1.5 T Siemens scanner as part of the UK Biobank Abdominal Imaging protocol. Detailed descriptions of the acquisition and processing protocols have been published previously (Littlejohns et al., 2020). PDFF is a reliable indicator of liver fat content calculated with water-fat separation masks, and PDFF ≥ 5.5% indicates hepatic steatosis (He et al., 2023). cT1 is a promising marker of fibro-inflammation derived from both T1 and T2* that is associated with adverse liver-related health events and cardiovascular disease events. A clinical cut-off of cT1 ≥ 800 ms was applied, consistent with its use as the upper bound of normal and as a recommended threshold for detecting progression from simple steatosis to NASH (Andersson et al., 2022), for predicting relapse or loss of sustained remission in autoimmune hepatitis (Arndtz et al., 2021), and for identifying mild fibrosis in heterogeneous chronic liver disease cohorts (Banerjee et al., 2014; McDonald et al., 2018).

We employed three classification methods to assess the effect size of PDFF and cT1 on brain-PAD. Classification 1 (Clinical Threshold Group) divides the population into two groups based on the aforementioned clinical thresholds. Classification 2 (Quartile Groups) categorizes the population into four groups according to the quartiles of PDFF or cT1. Additionally, to capture the dose-response relationship, we considered PDFF and cT1 as continuous variables. Given the right-skewed distribution of PDFF and cT1 (Supplementary Figure 2), we consistently utilized log-transformed values of PDFF and cT1 in our analyses.

### Acquisition of brain imaging-derived phenotypes

Brain MRI scans were performed using a Siemens Skyra 3T scanner. Detailed description of the protocol used to acquire and process brain MRI images has been published previously and are summarized in Supplementary Table 3.

A total of 1,079 IDPs were extracted across six MRI modalities (Dove et al., 2024): T1-weighted MRI (*n* = 165), T2-weighted fluid-attenuated inversion recovery (T2-FLAIR) (*n* = 1), T2* (*n* = 14), diffusion-MRI (*n* = 675), resting-state fMRI (*n* = 210), and task fMRI (*n* = 14). Briefly, T1-weighted imaging provides insights into the volume and thickness of various brain regions, while T2-FLAIR imaging identifies WMH, indicative of vascular brain damage. T2* imaging is employed to detect brain microbleeds, and diffusion MRI assesses the microstructural integrity of white matter. Resting-state fMRI measures brain activity during rest to evaluate the intrinsic functional connectivity of neural networks, whereas task fMRI examines brain activity when participants engage in a task or respond to a sensory stimulus, such as a face or shapes matching task (Miller et al., 2016). A full list of all 1,079 IDPs is included in Supplementary Table 1. The WMH volume data were obtained from T2-FLAIR MRI scans, which are highly sensitive to white matter lesions, including hyperintensities linked to small vessel pathology (Cote et al., 2023).

### Assessment of brain age and brain-PAD

The workflow for calculating brain age and brain-PAD is illustrated in Supplementary Figure 3, and the detailed description is available in the Supplementary material. First, we identified 4,333 healthy individuals aged between 40 and 70 years who had no ICD-10 diagnoses and were free from self-reported long-term illnesses, disabilities, or frailty (Field ID: 2188), as well as those reporting fair or poor health status (Field ID: 2178). We then randomly divided these participants into a training set (*n* = 3,466) and a validation set (*n* = 867) in a 4:1 ratio. Subsequently, we converted 1,079 IDPs to Z-standardized scores, and combined three feature selection strategies (no feature selector, FeatureWiz, and recursive feature elimination with cross validation) with three commonly used machine learning models [least absolute shrinkage and selection operator regression (LASSO), eXtreme gradient boosting, and support vector regression] for predicting brain age to obtain a total of nine models (The parameter ranges for hyperparameter optimization of the 9 models is shown in Supplementary Table 4). Bayesian optimization was used to fine-tune the hyperparameters of all nine models over 100 epochs (Supplementary Tables 5, 6). Following optimization, the models were applied to the validation set to facilitate performance comparison. Of the 9 models, the LASSO model without feature selection yielded the lowest mean absolute error (Supplementary Tables 7, 8) and was selected for predicting brain age across all participants.

It is important to note that brain age tends to be overpredicted in younger individuals and underpredicted in older individuals. To address this age bias, we corrected the brain age estimates using the following formula:

*brain age_*corrected*_* = * (brain age_*original*_ -*β*)/*α, where coefficients α and β represent the slope and intercept from the training set regression equation: *brain age_*training* *set*_* = α * chronological age_*training*_ _*set*_ + β (Dove et al., 2024; Huang et al., 2024; Supplementary Figure 4).

Brain-PAD was calculated as *brain-PAD* = *brain age_*corrected*_* -*chronological age_*time of MRI*_*. Positive values for Brain-PAD indicate a brain that is older than expected, while negative values indicate a brain that is younger than expected based on the individual’s chronological age.

### Covariates

Prior selection of covariates was conducted, including demographic factors (age, sex, ethnic background, and location of assessment center), socioeconomic factors (Townsend Deprivation Index, and education level), lifestyle factors [smoking status (current, former, and never), alcohol intake (continue), physical activity (high, moderate, and low)], BMI, regular social connection, and cardiometabolic burden. Alcohol intake was assessed following previously established methods (Liang et al., 2026). Supplementary method provides a detailed description of each covariate.

An optimal lifestyle was defined as never smoking, no or light/moderate alcohol consumption [≤ 2 units/day (16 g/day) according to current U.K. guidelines on alcohol consumption for both men and women], and high level of physical activity.

### Genetic predisposition

We utilized the Alzheimer disease (AD)-related polygenic risk score (PRS_*AD*_) and the *APOE* genotype to represent genetic predisposition. PRS_*AD*_ was derived from the UK Biobank’s Standard PRS Set (Thompson et al., 2024). Higher levels of PRS_*AD*_ indicate a greater genetic susceptibility to Alzheimer disease. In this study, we classified participants into three groups (low, intermediate, and high) based on tertiles of PRS_*AD*_. The *APOE* ε4 allele has been established as a significant genetic risk factor for AD; therefore, we determined the *APOE* genotype using the SNPs rs429358 and rs7412. Participants carrying the *APOE* ε4 allele (genotypes ε2/ε4, ε3/ε4, and ε4/ε4) were categorized as *APOE* ε4 carriers, while those without it (genotypes ε2/ε2, ε2/ε3, and ε3/ε3) were classified as *APOE* ε4 non-carriers.

### Statistical analysis

Continuous variables were presented as either mean [± standard deviation (SD)] or median (25th percentile, 75th percentile), while categorical variables were presented as counts (percentages). Linear regression models were employed to estimate effect value (Beta) and 95% confidence intervals (CIs) for the association of PDFF and cT1 with brain-PAD. The least-squares means of brain-PAD across different groups were additionally estimated from the margins of the linear regression models. We also utilized restricted cubic spline regression to investigate the potential non-linear relationship between PDFF/cT1 and brain-PAD. Subsequently, a stratified analysis was conducted to explore the influence of sex, cardiometabolic burden, lifestyle, *APOE* genotype, and PRS_*AD*_ on the association of PDFF and cT1 with brain-PAD. Furthermore, we performed a joint exposure analysis by incorporating a multi-category indicator variable that combined an imaging-derived metric [PDFF ( < 5.5% vs. ≥ 5.5%)/cT1 ( < 800 ms vs. ≥ 800 ms)] and these modifying factors into the linear regression model. Interactions were assessed by adding the cross-product term to the models. Model 1 was initially adjusted for age, sex, ethnic background, location of the assessment center, Townsend Deprivation Index, and education level, while Model 2 was further adjusted for smoking status, alcohol intake, physical activity, BMI, regular social connections, and cardiometabolic burden. To ascertain whether hepatic fibro-inflammation is a more significant driver of brain aging than liver fat, we additionally constructed a simultaneous model in which PDFF and cT1 were entered into the same regression. In reporting the results, we primarily focus on Models 2 and 3, with Model 3 serving as the main model because it more fully accounts for potential confounding arising from the interrelationship between PDFF and cT1. By modeling PDFF and cT1 jointly, we estimated their associations with brain age while controlling for each other. Because PDFF and cT1 were evaluated within a single pre-specified model and we did not conduct separate, independent hypothesis tests across multiple models, no multiple-testing correction was applied. To further ensure that collinearity did not materially affect coefficient estimation, we assessed multicollinearity using variance inflation factors.

In sensitivity analyses, we repeated the main analyses: (1) in a subsample without other known liver disease or excessive alcohol intake (Supplementary Table 9); (2) considering the clinical relevance of metabolic dysfunction-associated steatotic liver disease (MASLD) (a subgroup of the SLD cohort in which participants had at least one cardiometabolic risk factor but no other possible cause of steatosis), we used MASLD instead of SLD for our analysis, as metabolic dysfunction likely plays a more crucial role in the association between hepatic steatosis and brain aging in this subgroup; (3) using models that further adjusted for waist circumference or visceral fat; and (4) in different age groups (≤ 60 years vs. > 60 years). To elucidate potential mechanistic pathways, we also examined whether lifestyle factors modified the association between cT1 and PDFF and WMH volume. To account for between-individual variation in head size, WMH volume was adjusted for intracranial volume (WMH volume/intracranial volume) to derive a standardized metric (Wang et al., 2026). The resulting adjusted measure was then log-transformed to reduce positive skewness. In addition, given that cT1 may be sensitive to hepatic iron deposition, we excluded participants with a history of hemochromatosis from the analyses. We fitted the mediator and outcome models using linear regression and conducted causal mediation analyses using the R package mediation, with inference based on non-parametric bootstrap resampling (2,000 simulations). BMI and cardiometabolic burden were specified as mediators to assess whether the associations of PDFF and cT1 with brain-PAD persist beyond systemic metabolic dysfunction, thereby estimating their direct effects. All statistical analyses were performed using Python 3.9, as well as R software version 4.2.1, the details for software and algorithms can be available in Supplementary Table 10. Statistical significance was considered at a two-tailed *P* < 0.05.

## Results

### Characteristics of participants

Baseline characteristics of 19,566 participants [mean (SD) age: 63.42 (7.51) years; 10,169 females (52.0%)] are summarized in Table 1. A total of 4,634 participants (23.7%) had significant SLD (PDFF ≥ 5.5%), and 938 participants (4.8%) had significant fibro-inflammation (cT1 ≥ 800 ms). Individuals with SLD or high fibro-inflammation were more likely to be male and exhibited lower educational attainment. Additionally, they had higher rates of smoking, lower levels of physical activity, increased alcohol consumption, resided in more deprived areas, and presented with elevated blood pressure, BMI, and brain-PAD. Furthermore, these individuals had a higher prevalence of diabetes, hypertension, and hyperlipidemia (Table 1).

**TABLE 1:** Characteristics of the study population stratified by PDFF or cT1.

	Overall	PDFF < 5.5%	PDFF ≥ 5.5%	*P* for PDFF	cT1 < 800 ms	cT1 ≥ 800 ms	*P* for cT1
Characteristics	(*N* = 19,566)	(*N* = 14,932)	(*N* = 4,634)		(*N* = 1,8,628)	(*N* = 938)	
Age, y, mean ± SD	63.42 ± 7.51	63.39 ± 7.59	63.52 ± 7.25	0.285	63.47 ± 7.51	62.56 ± 7.36	< 0.001
Sex, *n* (%)				< 0.001			<0.001
Female	10,169 (52.0)	8,381 (56.1)	1,788 (38.6)		9,781 (52.5)	388 (41.4)	
Male	9,397 (48.0)	6,551 (43.9)	2,846 (61.4)		8,847 (47.5)	550 (58.6)	
TDI, n (%)	4,947 (25.3)	3,828 (25.6)	1,119 (24.1)	0.039	4,734 (25.4)	213 (22.7)	0.01
Q1				0.274			0.259
Q2	4,884 (25.0)	3,741 (25.1)	1,143 (24.7)		4,656 (25.0)	228 (24.3)	
Q3	4,899 (25.0)	3,739 (25.0)	1,160 (25.0)		4,676 (25.1)	223 (23.8)	
Q4	4,836 (24.7)	3,624 (24.3)	1,212 (26.2)		4,562 (24.5)	274 (29.2)	
Location of assessment center, n (%)	17,922 (91.6)	13,653 (91.4)	4,269 (92.1)		17,052 (91.5)	870 (92.8)	
England				< 0.001			<0.001
Scotland	1,326 (6.8)	1,039 (7.0)	287 (6.2)		1,276 (6.8)	50 (5.3)	
Stockport	269 (1.4)	205 (1.4)	64 (1.4)		253 (1.4)	16 (1.7)	
Wales	49 (0.3)	35 (0.2)	14 (0.3)		47 (0.3)	2 (0.2)	
Education level, n (%)	6,174 (31.6)	4,928 (33.0)	1,246 (26.9)		5,914 (31.7)	260 (27.7)	
High				0.587			0.571
Intermediate	12,506 (63.9)	9,409 (63.0)	3,097 (66.8)		11,898 (63.9)	608 (64.8)	
Low	886 (4.5)	595 (4.0)	291 (6.3)		816 (4.4)	70 (7.5)	
Ethnic background, n (%)	18,430 (94.2)	14,057 (94.1)	4,373 (94.4)		17,542 (94.2)	888 (94.7)	
White				0.26			0.073
Other	1,136 (5.8)	875 (5.9)	261 (5.6)		1,086 (5.8)	50 (5.3)	
Social connection, n (%)	1,411 (7.2)	1,059 (7.1)	352 (7.6)		1,329 (7.1)	82 (8.7)	
Irregular							
Regular	1,8155 (92.8)	1,3873 (92.9)	4,282 (92.4)		1,7299 (92.9)	856 (91.3)	
BMI, kg/m^2^, mean ± SD	0.00 ± 1.00	–0.24 ± 0.86	0.79 ± 1.02	< 0.001	–0.06 ± 0.95	1.20 ± 1.17	< 0.001
Smoking status, n (%)	613 (3.1)	470 (3.1)	143 (3.1)	< 0.001	581 (3.1)	32 (3.4)	<0.001
Current				< 0.001			< 0.001
Former	6,529 (33.4)	4,789 (32.1)	1,740 (37.5)		6,151 (33.0)	378 (40.3)	
Never	1,2424 (63.5)	9,673 (64.8)	2751 (59.4)		1,1896 (63.9)	528 (56.3)	
Alcohol intake, g/w, median (25th percentile, 75th percentile)	72.00 (22.33, 132.00)	68.00 (22.33, 124.00)	80.00 (21.40, 168.00)		72.00 (23.26, 132.00)	48.00 (8.37, 128.00)	
Physical activity, n (%)	9,373 (47.9)	7,583 (50.8)	1,790 (38.6)	< 0.001	9067 (48.7)	306 (32.6)	<0.001
High				< 0.001			<0.001
Moderate	8,132 (41.6)	6,019 (40.3)	2,113 (45.6)		7,708 (41.4)	424 (45.2)	
Low	2,061 (10.5)	1,330 (8.9)	731 (15.8)		1,853 (9.9)	208 (22.2)	
Hyperlipidemia, n (%)	16,657 (85.1)	12,989 (87.0)	3,668 (79.2)		15,922 (85.5)	735 (78.4)	
No				< 0.001			<0.001
Yes	2,909 (14.9)	1,943 (13.0)	966 (20.8)		2,706 (14.5)	203 (21.6)	
Hypertension, n (%)	7,767 (39.7)	6,478 (43.4)	1,289 (27.8)		7,513 (40.3)	254 (27.1)	
No				< 0.001			<0.001
Yes	11,799 (60.3)	8,454 (56.6)	3,345 (72.2)		11,115 (59.7)	684 (72.9)	
Type 2 diabetes, n (%)	18,610 (95.1)	14,487 (97.0)	4,123 (89.0)		17,828 (95.7)	782 (83.4)	
No				< 0.001			< 0.001
Yes	956 (4.9)	445 (3.0)	511 (11.0)		800 (4.3)	156 (16.6)	
SBP, mmHg, mean ± SD	140.66 ± 19.79	138.87 ± 19.80	146.40 ± 18.62		140.35 ± 19.76	146.67 ± 19.44	
Brain-PAD, y, mean ± SD	0.28 ± 5.06	0.03 ± 4.92	1.09 ± 5.40	< 0.001	0.20 ± 4.97	1.97 ± 6.29	< 0.001

SD, standard deviation; PDFF, proton density fat fraction; cT1, iron-corrected T1 mapping; BMI, body mass index; TDI, Townsend deprivation index; SBP, systolic blood pressure; Brain-PAD, brain-Predicted Age Difference.

### Steatotic liver disease, fibro-inflammation, and brain-PAD

In Model 2, compared to individuals with PDFF < 5.5%, those with PDFF ≥ 5.5% exhibited a significantly increased brain-PAD (β = 0.54 [95% CI 0.36, 0.73]). A 1-unit increase in log-transformed PDFF was associated with 0.42 (95% CI 0.29, 0.55) years increase in brain-PAD. Furthermore, individuals in the highest quartiles of PDFF demonstrated a significantly higher brain-PAD than those in the first quartile [β = 0.62 (95% CI 0.38, 0.86)]. A similar pattern was observed for cT1, where cT1 ≥ 800 ms was associated with an increased brain-PAD [β = 1.27 (95% CI 0.93, 1.61)]. Specifically, a 1-unit increase in log-transformed cT1 correlated with 5.13 (95% CI 4.10, 6.16) years increase in brain-PAD. Brain-PAD was observed to rise as high as 0.89 (95% CI 0.68, 1.11) years among individuals in the highest quartiles of cT1 (Table 2).

**TABLE 2:** Association of PDFF and cT1 with brain-PAD.

Liver imaging-derived metrics	No.	Model 1		Model 2		Model 3		Mean ± SE*
		Beta (95%CI)	*P-*value	Beta (95%CI)	*P-*value	Beta (95%CI)	*P-*value	
**PDFF**								
Continuous (log form)	19566	0.79 (0.68, 0.89)	< 0.001	0.42 (0.29, 0.55)	< 0.001	0.18 (0.03, 0.32)	0.015	
<5.5%	14932	Reference	< 0.001	Reference	< 0.001	Reference	< 0.001	1.33 (1.03, 1.63)
≥ 5.5%	4634	1.03 (0.87, 1.20)		0.54 (0.36, 0.73)		0.40 (0.21, 0.59)		1.73 (1.42, 2.03)
Q1	5527	Reference	0.003	Reference	0.981	Reference	< 0.001	0.77 (0.30, 1.24)
Q2	4435	0.31 (0.10, 0.51)		0.00 (−0.21, 0.20)		0.01 (−0.20, 0.21)		0.77 (0.30, 1.24)
Q3	4767	0.70 (0.50, 0.90)	< 0.001	0.15 (−0.07, 0.37)	0.175	0.11 (−0.11, 0.32)	0.335	0.87 (0.41, 1.34)
Q4	4837	1.38 (1.18, 1.58)	< 0.001	0.62 (0.38, 0.86)	< 0.001	0.39 (0.14, 0.64)	0.003	1.15 (0.68, 1.63)
**cT1**								
Continuous (log form)	19566	6.07 (5.13, 7.02)	< 0.001	5.13 (4.10, 6.16)	< 0.001	4.49 (3.34, 5.64)	< 0.001	
<800 ms	18628	Reference	< 0.001	Reference	< 0.001	Reference	< 0.001	0.98 (0.73, 1.23)
≥ 800 ms	938	1.70 (1.37, 2.03)		1.27 (0.93, 1.61)		1.09 (0.74, 1.44)		2.08 (1.67, 2.48)
Q1	5028	Reference	0.012	Reference	0.008	Reference	0.015	0.55 (0.08, 1.02)
Q2	4947	0.25 (0.06, 0.45)		0.26 (0.07, 0.46)		0.24 (0.05, 0.44)		0.80 (0.33, 1.27)
Q3	4716	0.45 (0.25, 0.66)	< 0.001	0.40 (0.20, 0.60)	< 0.001	0.35 (0.14, 0.55)	< 0.001	0.90 (0.43, 1.37)
Q4	4875	1.09 (0.89, 1.29)	< 0.001	0.89 (0.68, 1.11)	< 0.001	0.76 (0.54, 0.99)	< 0.001	1.31 (0.85, 1.78)

PDFF, proton density fat fraction; cT1, iron-corrected T1 mapping, Brain-PAD, brain-Predicted Age Difference, SE, standard error. Model 1 was adjusted for age, sex, location of assessment center, ethnic background, education level, Townsend deprivation index. Model 2 was adjusted for age, sex, location of assessment center, ethnic background, education level, Townsend deprivation index, smoking status, alcohol intake, physical activity, social connection, cardiometabolic burden, and BMI. Model 3 was simultaneous model in which PDFF and cT1 were modeled together on the basis of Model 2.

*Calculated based on simultaneous model.

**FIGURE 1:**
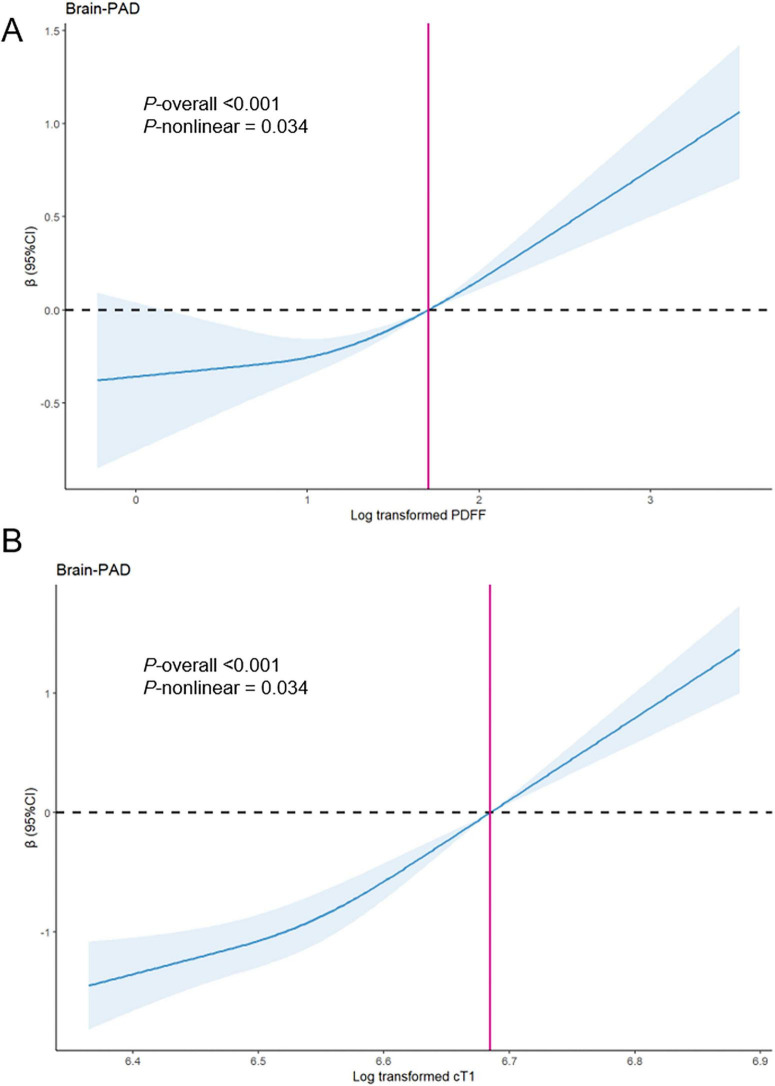
Multiple adjusted restricted cubic splines showing Brain-PAD associated with log transformed PDFF **(A)** and log transformed cT1 **(B)**. Brain-PAD, brain-Predicted Age Difference; PDFF, proton density fat fraction; cT1, iron-corrected T1 mapping. The blue line and blue shaded area represent the beta and 95% CIs, respectively, relative to the reference level (red vertical lines). The red vertical lines represent the log transformed value for PDFF = 5.5% or cT1 = 800 ms. Model was simultaneous model combining PDFF and cT1 with further adjustments for age, sex, location of assessment center, ethnic background, education level, Townsend deprivation index, smoking status, alcohol intake, physical activity, social connection, cardiometabolic burden, and BMI.

In the simultaneous model, both PDFF and cT1 remained significantly associated with higher brain-PAD, although the associations between PDFF and brain-PAD were attenuated. PDFF ≥ 5.5% [β = 0.4 (95% CI 0.21, 0.59)] and cT1 ≥ 800 ms [β = 1.09 (95% CI 0.74, 1.44)] were both associated with an increase in brain-PAD. Specifically, brain age was, on average, 1.33 years older than chronological age among individuals with PDFF < 5.5% and 1.73 years older among those with PDFF ≥ 5.5%. Furthermore, brain age was, on average, 0.98 years older than chronological age among individuals with cT1 < 800 ms and 2.08 years older among those with cT1 ≥ 800 ms (Table 2). No evidence of substantial multicollinearity was observed in our models, as the adjusted generalized variance inflation factors {GVIF^[1/(2⋅Df)]} were only slightly above 1, indicating minimal collinearity (Supplementary Table 11). Restricted cubic spline analysis indicated a positive relationship between PDFF and both cT1 and brain-PAD. Of note, the rate of increase in brain-PAD was found to rise with higher levels of PDFF and cT1, especially after the clinical threshold (red vertical lines) (*P* for non-linear < 0.05) (Figure 1).

Given the pathological correlation between liver fat and liver fibro-inflammation, we divided the sample according to the clinical threshold for PDFF (≥ 5.5%/ < 5.5%) as well as the median (> 3%/ ≤ 3%) to determine whether the strength of the association between cT1 and brain-PAD differed between groups. Interestingly, we found that the association between cT1 and increased brain-PAD was stronger in the group of PDFF < 5.5% or PDFF < 3%, although no significant interaction was identified (*P* for interaction > 0.05) (Supplementary Tables 12, 13).

### Role of sex and cardiometabolic burden

In stratified analyses of the simultaneous model, the association between PDFF ≥ 5.5% and higher brain-PAD was more pronounced in males [β_*male*_ = 0.37 (0.11, 0.64) vs. 0.31 (95% CI 0.04, 0.59)] (*P* for interaction < 0.001) (Supplementary Table 14) and in individuals with a higher cardiometabolic burden (β_*higher cardiometabolic burden*_ = 0.47 [95% CI 0.22, 0.72] vs. 0.29 [−0.02, 0.59]) (*P* for interaction = 0.019) (Supplementary Table 15). A similar pattern was observed for cT1 ≥ 800 ms [sex-stratified, β_*male*_ = 1.43 (95% CI 0.95, 1.91) vs. 0.51 (0.00, 1.02) (*P* for interaction < 0.001); cardiometabolic burden-stratified, β_*higher cardiometabolic burden*_ = 1.12 (95% CI 0.68, 1.55) vs. 0.80 (0.16, 1.45) (P for interaction = 0.09)]. In joint exposure analysis, brain age was 1.61 years older than chronological age among males with PDFF ≥ 5.5%, compared to 0.96 years for males with PDFF < 5.5%. For males with cT1 ≥ 800 ms, brain-PAD increased to 2.36 years older, in contrast to 0.65 years older for males with cT1 < 800 ms; this difference was less pronounced in females (1.54 years vs. 1.32 years) (Supplementary Figure 5). Furthermore, among individuals with a higher cardiometabolic burden (≥ 2 risk factors), PDFF ≥ 5.5% and cT1 ≥ 800 ms were associated with an average brain-PAD of 2.22 and 2.62 years, respectively, compared to 1.18 and 1.29 years among their counterparts with a lower cardiometabolic burden (0-1 risk factors) (Supplementary Figure 6).

### Role of genetic predisposition

**FIGURE 2:**
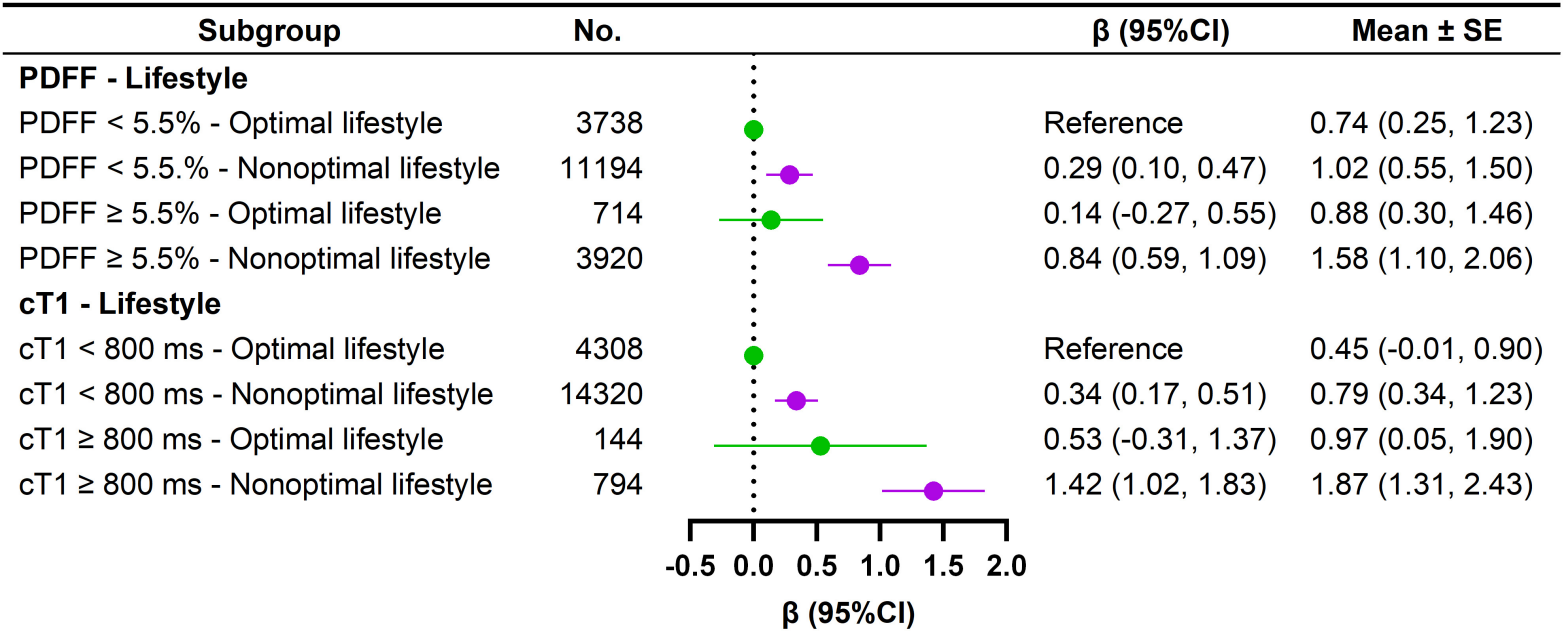
Joint effects of PDFF, cT1, *APOE*ε*4*, and PRS_*AD*_ on brain-PAD. SE, standard error; PDFF, proton density fat fraction; cT1, iron-corrected T1 mapping; PRS_*AD*_, Alzheimer disease (AD)-related polygenic risk score. Model was simultaneous model combining PDFF and cT1 with further adjustments for age, sex, location of assessment center, ethnic background, education level, Townsend deprivation index, smoking status, alcohol intake, physical activity, social connection, cardiometabolic burden, and BMI.

*APOE*ε4 carriers exhibited a 0.18-year higher brain-PAD (95% CI: 0.02–0.34). In analyses stratified by *APOE* ε4 status, the association of PDFF ≥ 5.5% [β_*carriers*_ = 0.51 (95% CI 0.10, 0.91) vs. 0.32 (0.10, 0.53)] and cT1 ≥ 800 ms [β_*carriers*_ = 1.47 (95% CI 0.69, 2.25) vs. 1.07 (95% CI 0.67, 1.46)] with elevated brain-PAD was more pronounced in *APOE* ε4 carriers compared to non-carriers, although significant interactions were not observed (All *P* for interaction > 0.05) (Supplementary Table 16). In joint exposure analysis, *APOE* ε4 carriers with PDFF ≥ 5.5% were associated with 0.62 (95% CI 0.29, 0.95) years increase in brain-PAD relative to non-carriers with PDFF < 5.5%. Moreover, *APOE* ε4 carriers with cT1 ≥ 800 ms had a 1.64-year higher brain-PAD (95% CI: 0.93–2.35) than non-carriers with cT1 < 800 ms (Figure 2).

We observed no association between PRS_*AD*_ and brain-PAD. However, the association between PDFF ≥ 5.5% and higher brain-PAD was particularly pronounced in individuals with intermediate and high PRS_*AD*_ (*P* for interaction = 0.158) (Supplementary Table 17). Joint exposure analyses indicated that individuals with PDFF ≥ 5.5% and high PRS_*AD*_ exhibited the highest brain-PAD [β = 0.54 (95% CI 0.23, 0.85)] compared to those with PDFF < 5.5% and low PRS_*AD*_. Furthermore, those with cT1 ≥ 800 ms and intermediate PRS_*AD*_ had the highest brain-PAD [β = 1.39 (95% CI 0.82, 1.96)] compared to individuals with cT1 < 800 ms and low PRS_*AD*_ (Figure 2).

### Healthy lifestyle attenuates the excess brain-PAD caused by high PDFF and cT1

**FIGURE 3:**
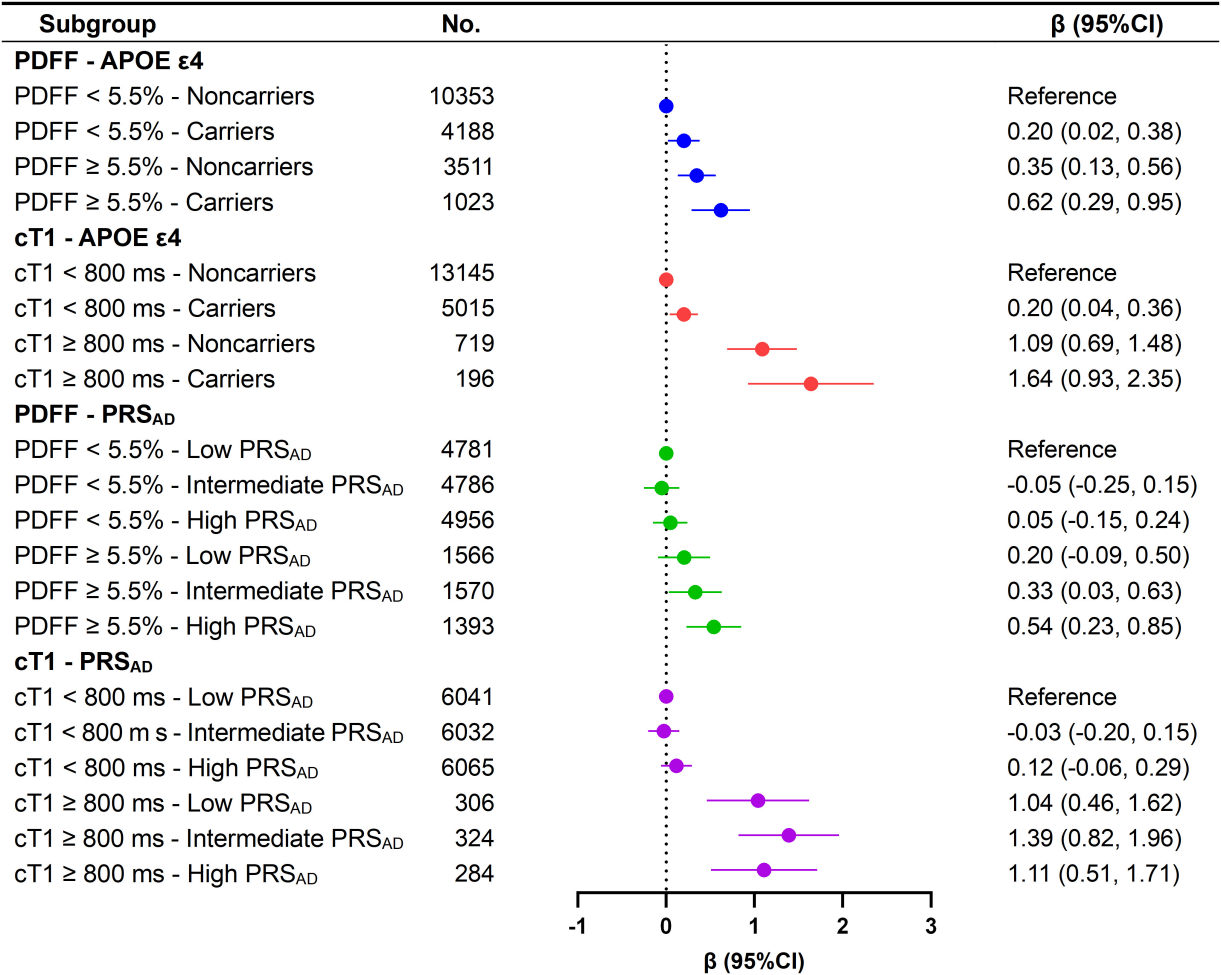
Joint effects of PDFF, cT1, and lifestyle on brain-PAD. SE, standard error; PDFF, proton density fat fraction; cT1, iron-corrected T1 mapping. Model was simultaneous model combining PDFF and cT1 with further adjustments for age, sex, location of assessment center, ethnic background, education level, Townsend deprivation index, social connection, cardiometabolic burden, and BMI.

Lifestyle-stratified analyses revealed that the association of PDFF ≥ 5.5% [β = 0.52 (0.30, 0.73)] and cT1 ≥ 800 ms [β = 1.04 (95% CI 0.65, 1.43)] with higher brain-PAD was significant only among individuals with a non-optimal lifestyle (Supplementary Table 18). Furthermore, PDFF, when treated as a continuous variable, was significantly associated with elevated brain-PAD exclusively in those with a non-optimal lifestyle [β = 0.45 (95% CI 0.29, 0.61)] (*P* for interaction < 0.001). In a joint exposure analysis, an optimal healthy lifestyle mitigated the association of PDFF ≥ 5.5% and cT1 ≥ 800 ms with brain-PAD. Specifically, individuals with PDFF ≥ 5.5% and an optimal lifestyle exhibited an average brain age that was 0.88 years older than their chronological age, compared to 1.58 years older among those with PDFF ≥ 5.5% and a non-optimal lifestyle (Figure 3). A similar pattern was observed for cT1, where brain-PAD was, on average, 0.97 years older among individuals with cT1 ≥ 800 ms and an optimal lifestyle, compared to 1.87 years among those with cT1 ≥ 800 ms and a non-optimal lifestyle. Overall, a healthy lifestyle was associated with a reduction in brain-PAD of approximately 0.7–0.9 years among individuals with PDFF ≥ 5.5% or cT1 ≥ 800 ms.

### Sensitivity analysis

Sensitivity analyses indicated that the observed associations remained largely unchanged when we repeated the analyses: (1) in a subsample excluding individuals with other known liver diseases or excessive alcohol intake (Supplementary Table 19); (2) utilizing MASLD instead of SLD (Supplementary Table 20); (3) employing models that further adjusted for waist circumference or visceral fat (Supplementary Table 21); and 4) in different age group (Supplementary Table 22). In addition, both PDFF and cT1 were associated with increased standardized WMH. The association between cT1 and WMH was no longer evident among individuals with healthy lifestyle choices, although no significant interaction was detected (Supplementary Table 23). After excluding participants with hemochromatosis, our conclusion remained unchanged (Supplementary Table 24). Finally, mediation analyses indicated that both cT1 and PDFF were directly associated with brain-PAD. BMI and cardiometabolic burden mediated 23.3 and 21.0% of the association between elevated cT1 (≥ 800 ms vs. < 800 ms) and brain-PAD, respectively, and mediated 32.3 and 37.3% of the association between elevated PDFF (≥ 5.5% vs. < 5.5%) and brain-PAD, respectively (Supplementary Figure 7).

## Discussion

Our findings showed that both hepatic steatosis (PDFF) and hepatic fibro-inflammation (cT1) were positively associated with higher brain-PAD, and these associations remained after extensive adjustment and simultaneous inclusion of PDFF and cT1 in the same model. Overall, the association with brain-PAD was stronger for cT1, suggesting that fibro-inflammatory activity may better capture pathophysiological processes related to brain aging than liver fat alone. Using clinically relevant thresholds, participants with cT1 ≥ 800 ms had approximately 1.09 years higher brain-PAD than those with cT1 < 800 ms (2.08 vs. 0.98 years), whereas PDFF ≥ 5.5% was associated with a smaller difference of about 0.40 years compared with PDFF < 5.5% (1.73 vs. 1.33 years). Given prior evidence that each additional year of brain-PAD is associated with an approximately 3–9% higher risk of future dementia diagnosis (Biondo et al., 2022; Wang et al., 2019), these brain-PAD differences translate—roughly—into an estimated 3–10% (cT1) and 1–4% (PDFF) relative increase in risk, suggesting potential clinical relevance.

A previous study found that NAFLD is linked to approximately 4.2 years of brain aging (Weinstein et al., 2018), which is significantly higher than our estimated effect. This discrepancy can be attributed to differing methods of estimating brain age and definition of SLD. Most prior studies have relied solely on T1-weighted imaging to estimate brain age, but our study integrates information from six brain MRI modalities (T1-weighted imaging, T2-FLAIR, T2*, diffusion MRI, resting-state fMRI, and task fMRI). Although T1-weighted imaging provides the highest independent accuracy for brain age estimation, a combination of multiple MRI modalities yields optimal results (Cole, 2020). Of note, the accumulation of liver fat, even in the absence of NAFLD, has been associated with adverse health outcomes (Arold et al., 2024). While earlier studies have primarily examined the relationship between NAFLD and structural brain imaging (Weinstein et al., 2018; Weinstein et al., 2024), MRI-derived PDFF offers the opportunity to elucidate a non-linear dose-response relationship between subtle liver fat accumulations and increased brain age. Our findings indicate that even subtle accumulations of liver fat can elevate brain-PAD, with the rate of increase accelerating alongside liver fat accumulation. This underscores the necessity of quantifying liver fat for the screening of high-risk populations.

We reveal, for the first time, the link between liver fibro-inflammation and brain age, which is independent of waist circumference, liver fat, and visceral fat. Recent studies have found that liver fibro-inflammation, but not liver fat content, is independently associated with new-onset cardiovascular disease and all-cause mortality (Roca-Fernandez et al., 2023). Interestingly, the association between cT1 and increased brain-PAD was stronger in those with PDFF < 5.5%, and the association between liver fat and increased brain age is significantly attenuated when liver cT1 is included in the model, suggesting that fibro-inflammation, rather than liver fat, is the primary driver of brain aging. Consistently, a study on brain phenotypes found that liver fat did not directly associate with adverse brain phenotypes when liver cT1 and PDFF were modeled simultaneously (McCracken et al., 2022). Indeed, lower PDFF is a common feature of advanced CLD and may not serve as a reliable biomarker for risk stratification (Roca-Fernandez et al., 2023). Encouragingly, early liver fibro-inflammation is reversible. Lifestyle interventions such as exercise and a healthy diet are associated with reduced liver fibro-inflammation (Chen et al., 2024; Sherry et al., 2023). Thus, the potential benefits to brain health provide an additional motivation for lifestyle therapies aimed at promoting liver health.

The association of liver fat and liver fibro-inflammation with brain-PAD was found to be stronger in males and in individuals with a higher cardiometabolic burden. These results highlight the complex interplay between CLD, sex, and cardiometabolic factors in relation to brain health, emphasizing the importance of identifying individuals who may benefit most from interventions. Healthy lifestyles have been shown to mitigate the impact of risk factors on health outcomes (Marseglia et al., 2020). Our study documented a mitigating effect of lifestyle behaviors on the association of liver fat and fibro-inflammation with brain-PAD, although further interventional studies are needed to verify these findings.

Vascular injury may represent a potential mechanism underlying the liver-brain axis (You et al., 2015). Abnormal liver function may influence brain health through multiple mechanisms. On the one hand, excessive fat accumulation in the liver can induce hepatocellular stress, leading to cell death and the release of hepatokines or cytokines, which in turn promote hepatic and systemic inflammation (Peiseler et al., 2022). Such inflammatory processes may not only accelerate the progression of liver disease but may also disrupt peripheral amyloid-β (Aβ) clearance, thereby exacerbating Alzheimer’s disease–related pathology, suggesting that the liver–brain relationship could be bidirectional (Weinstein et al., 2022). However, given the cross-sectional design, our findings indicate correlations rather than causality, and the possibility of reverse causation should be considered. For example, brain aging—particularly Aβ accumulation in Alzheimer’s disease—may affect metabolic pathways via neuroendocrine and hormonal regulation, thereby contributing to hepatic fat deposition, metabolic dysregulation, and the development of liver fibrosis (Tsay et al., 2024). This reverse pathway could play an important role in liver disease progression, where brain aging–related metabolic disturbances may increase hepatic burden, promote chronic systemic and hepatic inflammation, and potentially create a vicious cycle. In addition, residual confounding may partially explain the observed associations, as lifestyle factors (e.g., diet, physical activity, smoking) and genetic susceptibility (e.g., metabolic syndrome, obesity) may simultaneously influence both liver and brain health and may not be fully captured in our analyses. Therefore, future studies should incorporate longitudinal designs to track temporal changes in liver health and brain aging to better clarify directionality and causality, and causal inference approaches such as Mendelian randomization should also be considered.

The strength of this study lies in the utilization of a large sample with comprehensive imaging phenotypic data, including precise estimates of brain age, liver fat content, and fibro-inflammation. However, several limitations should be acknowledged. First, the UK Biobank is subject to healthy volunteer bias and selection bias, which may limit the generalizability of our findings to broader and more clinically diverse populations. Second, although we adjusted for a range of covariates, we could not fully account for residual and unmeasured confounding—particularly participant-level factors such as detailed dietary patterns, medication use, lifetime socioeconomic circumstances, comorbidities, and other health behaviors—which may partially explain the observed associations. Third, the cross-sectional design precludes establishing temporality and restricts causal inference. In UK Biobank, liver and brain MRI measures were obtained during the same initial imaging assessment visit (instance 2), typically within a single visit window, which minimizes long time gaps between exposure and outcome measurements. Nevertheless, scans are acquired sequentially and may still be separated by hours, and short-term physiological variability (e.g., fasting status or transient metabolic/inflammatory fluctuations) could introduce non-differential measurement error, likely biasing associations toward the null. Further research, including longitudinal designs and causal inference approaches, is warranted to clarify directionality and to better establish the clinical validity of these metrics.

## Conclusion

The accumulation of liver fat and fibro-inflammation was associated with accelerated brain aging, with fibro-inflammation serving as a significant driving factor. These associations were more pronounced in males and in individuals with a high cardiometabolic burden, but may be attenuated in those with healthy lifestyle choices. Given that SLD and fibro-inflammation are reversible, if our findings are validated in prospective studies and clinical trials, this may suggest that the prevention and treatment of SLD and fibro-inflammation could provide substantial extra-hepatic benefits, including the potential preservation of brain function.

## Data Availability

The original contributions presented in this study are included in the article/Supplementary material, further inquiries can be directed to this corresponding authors.
