# Supplementary material for: The role of healthy lifestyle in the association between hepatic fibro-inflammation and steatosis and brain aging—a cross-sectional study
Source: Front Aging Neurosci. 2026 Mar 16;18:1801577. doi: 10.3389/fnagi.2026.1801577 (PMC13033754; doi:10.3389/fnagi.2026.1801577)
Supplement: Supplementary file 1 [file Data_Sheet_1.docx]

**
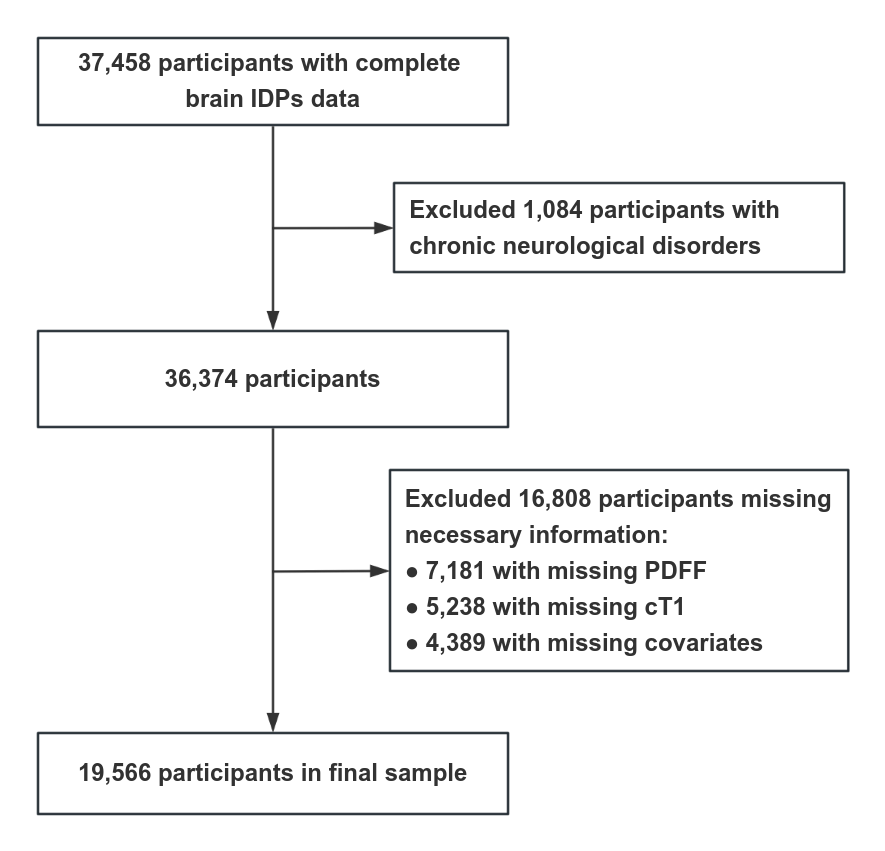
**

**sFigure 1 Flowchart of participants included in the study.**

Abbreviations: PDFF, proton density fat fraction; cT1, iron-corrected T1 mapping; IDPs, Imaging-Derived Phenotypes


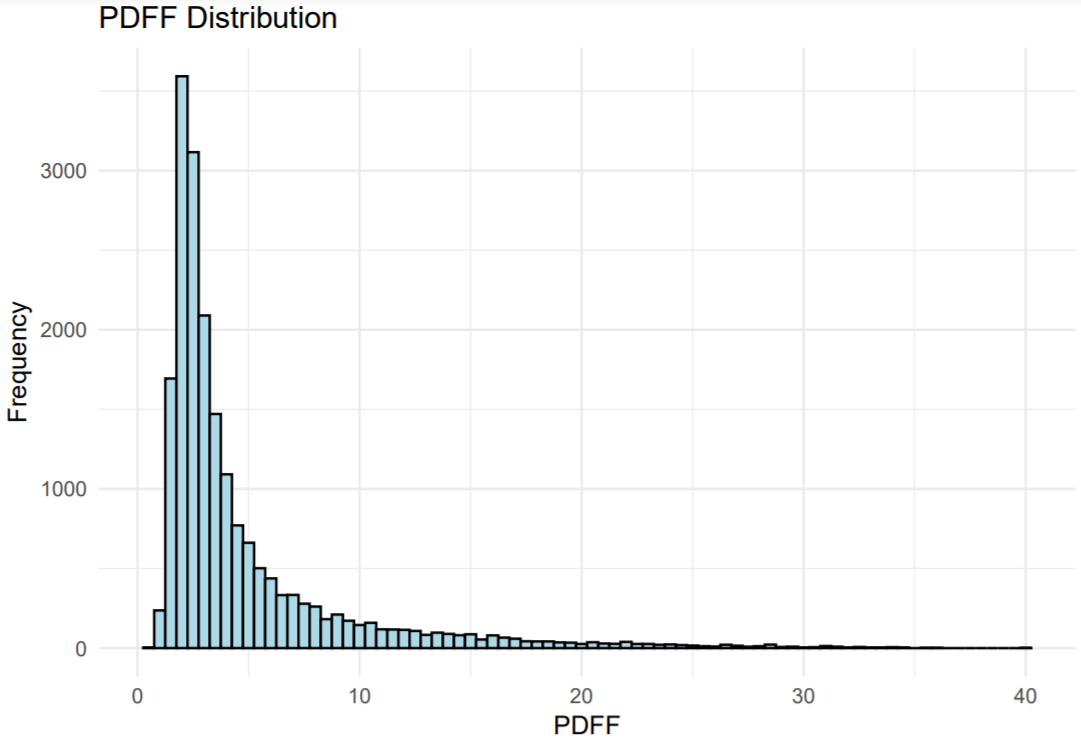

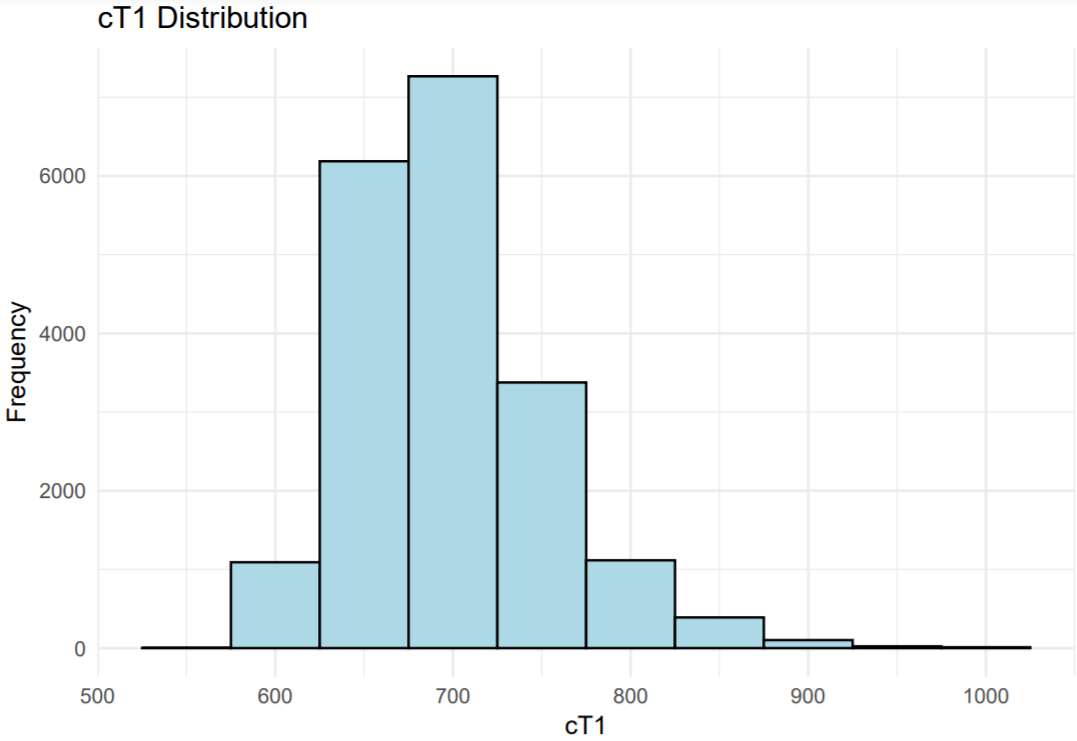


**sFigure 2 Distribution of PDFF and cT1.**

Abbreviations: PDFF, proton density fat fraction; cT1, iron-corrected T1 mapping

**Supplementary methods**

Definition of covariates

Covariates includes age (continue), sex, location of assessment center (England, Scotland, and Wales), Townsend Deprivation Index (quartiles), education level (high [college/university degree or above], intermediate [advanced/advanced subsidiary levels, ordinary levels, general certificate of secondary education, certificate of secondary education, national vocational qualification or higher national diploma, or equivalent, and other professional qualifications] and low [none of the above]), smoking status (current, former, and never), alcohol intake (continue), physical activity (high, moderate, and low), body mass index (continue), social connection (regular [“almost daily,” “2–4 times a week,” “about once a week,” and “about once a month”] and irregular [“once every few months,” “never or almost never,” and “no friends/family outside household”]), and cardiometabolic burden (ranging from 0 to 4).

To collect information on alcohol intake, a computer-assisted touch-screen system was utilized during the UK Biobank interview. Participants were asked to report their average alcohol consumption on a weekly or monthly basis, specifying the number of glasses of red wine, glasses of champagne or white wine, pints of beer or cider, measures of spirits, glasses of fortified wine, and glasses of other types of alcoholic drinks. For those who did not consume alcohol weekly or who drank occasionally, the amount of alcohol consumed was reported in 'average months' to provide a more reliable estimate for infrequent drinkers. To calculate the average alcohol consumption per participant, it was assumed that a pint of beer or cider contained 2 units (16g) of pure alcohol; a glass of red wine, champagne, white wine, as well as spirits and 'other' alcoholic beverages contained 1.5 units (12g) of alcohol; and a glass of spirits contained 1 unit (8g) of alcohol. The total monthly grams were divided by 30 to calculate the average daily intake. Excessive alcohol intake was defined as consumption exceeding 30g/day for men and 20g/day for women.

Physical activity was self-reported and categorized into three levels (high, moderate, and low) using the International Physical Activity Questionnaire (IPAQ). The Townsend deprivation index combines information on social class, employment, car availability, and housing, and is classified into quartiles, with higher quartiles indicating greater levels of area-based socioeconomic deprivation.

A score reflecting cardiometabolic burden, ranging from 0 to 4, was generated based on the total number of cardiometabolic risk components present  [1] .

These components include:

- BMI > 25 kg/m2 OR waist circumference > 94 cm (male) 80 cm (female).
- Type 2 diabetes (self-reported [field 1712], ICD-10 summary code E11) OR antidiabetic drug medication (field 6177 and 6153; field 20003 entries “metformin”, “rosiglitazone 1mg / metformin 500mg tablet”, “glimepiride”, “gliclazide”, “pioglitazone”, “rosiglitazone”).
- Blood pressure > 130/85 mmHg OR antihypertensive drug treatment (field 6177 and 6153).
- ICD-10 summary code E78.5 and Lipid lowering treatment (field 6177 and 6153).

Additional plasma-based cardiometabolic risk factors were not included, as blood sampling occurred, on average, 10 years prior to the data collection used in this study (imaging visit).

Definition of MASLD

Participants with MASLD constituted a subgroup of the SLD cohort, characterized by the presence of at least one cardiometabolic risk factor and the absence of other potential causes for steatosis. Specifically, this included no excessive alcohol intake (defined as < 30 g/day for men and < 20 g/day for women) and no other liver diseases (see Supplementary Table 9).

**Detailed description for calculating the brain age**

The workflow of the models training is as follows.


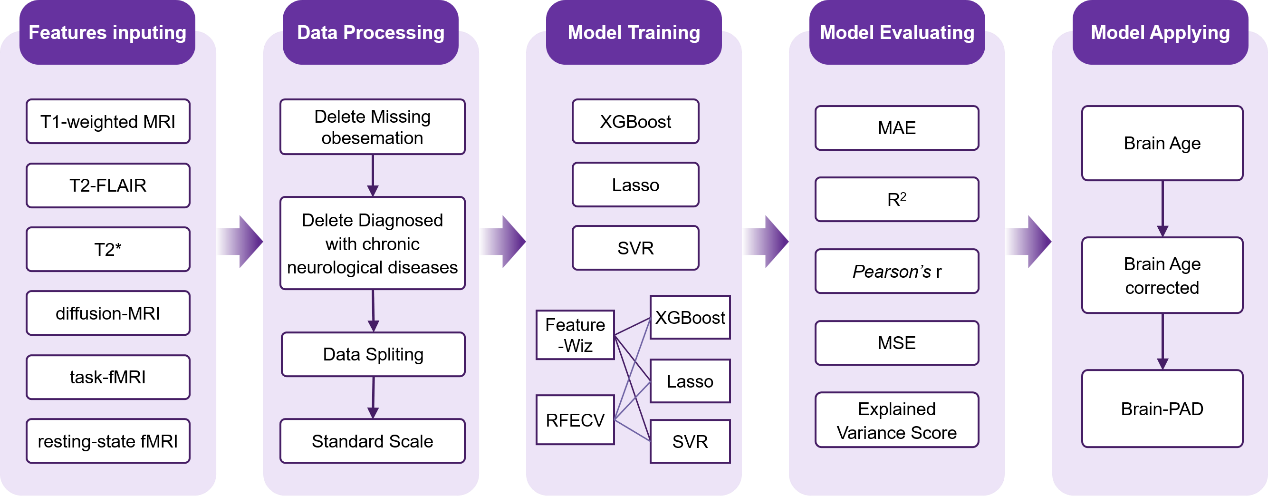


**sFigure 3 Workflow to model brain age.**

* Abbreviation: MAE, mean absolute error; MSE, mean square error; RFECV, recursive feature elimination cross-validation; SVR, support vector regression; XGBoost, eXtreme Gradient Boosting.

**Feature Inputting.**

A total of 1,079 Imaging-Derived Phenotypes (IDPs) were incorporated from six MRI modalities (Supplementary Table 1): T1-weighted MRI, T2-FLAIR, diffusion MRI, task fMRI, and resting-state fMRI, as detailed in Supplementary Table 3. The variable in Resting-state fMRI is a partial correlation matrix with 25 dimensionalities, that had been decoded into 210 elements.

**Data Processing.**

We excluded participants with missing IDPs, resulting in a final sample of 37,458 individuals. We selected healthy participants for model training. The healthy participant is defined as one without ICD-10 diagnoses (Field ID: 41270), long-standing illness, disability, or infirmity (Field ID: 2188), diabetes (Field ID: 2443), or stroke (Field ID: 4056), and who rated their overall health as excellent or good (Field ID: 2178). A total of 4,333 healthy participants were randomly divided into a training set (3466) and a test set (867) to facilitate model training and performance verification. Finally, all 1,079 IDPs were standardized using Z-score conversion.

**Model training.**

We combined three feature selection strategies (no feature selector, FeatureWiz, and recursive feature elimination with cross validation) with three commonly used machine learning models (least absolute shrinkage and selection operator regression [LASSO], eXtreme gradient boosting, and support vector regression) for predicting brain age to obtain a total of nine models. Feature-Wiz is a *featurewiz* package implemented in Python, utilizing the well-established Minimum Redundancy Maximum Relevance (MRMR) algorithm. Detailed usage instructions can be found at https://github.com/AutoViML/featurewiz. RFECV, imported from Python's *sklearn* package via *from sklearn.feature_selection import RFECV*, employs cross-validation based on recursive feature elimination (RFE) to retain the features that demonstrate optimal performance. LASSO, eXtreme gradient boosting, and support vector regression were implemented using Python's *sklearn*, *xgboost,* and *sklearn* packages, respectively. Bayesian optimization (init_points = 10, n_iter = 100) was employed to identify the optimal hyperparameters for the nine models, utilizing five-fold cross-validation, with '*neg_mean_squared_error*' as the evaluation metric. The hyperparameter ranges for each model and the corresponding best hyperparameter results are available in Supplementary Table 4 and 5. The best hyperparameters for each model were then applied to fit the training set (5-fold cross-validation), followed by testing on the test set. The process of Bayesian optimization is detailed in Supplementary Table 6.

**Model Evaluating.**

We used several metrics including Mean Absolute Error (MAE), R-squared (R2), Pearson's correlation coefficient (r), Mean Squared Error (MSE), and Explained Variance Score to assess the model's performance. The performance results for the nine models are presented in Supplementary Table 7. The LASSO model without feature selection was adopted as the final model because of its ideal performance in training set and test set. In this model, 236 of the 1,079 IDPs contributed significantly to the brain age estimate (Supplementary Table 8).

**Model applying**

The LASSO model without feature selection model, after being trained by all healthy samples, was utilized to predict brain age in a cohort of non-healthy participants. By integrating the predicted brain ages of both healthy and non-healthy individuals, the overall brain age of all participants can be determined.

Brain age tends to be overpredicted in younger individuals and underpredicted in older individuals [2]. To address this age bias, we corrected the brain age estimates using the following formula: *brain age_corrected_ =  (brain age_original_ - β) / α*, where coefficients α and β represent the slope and intercept from the training set regression equation: *brain age_training set_ = α * chronological age_training set_ + β* [3, 4]. Brain-PAD was calculated as *brain-PAD = brain age_corrected_ – chronological age_time of MRI_*.


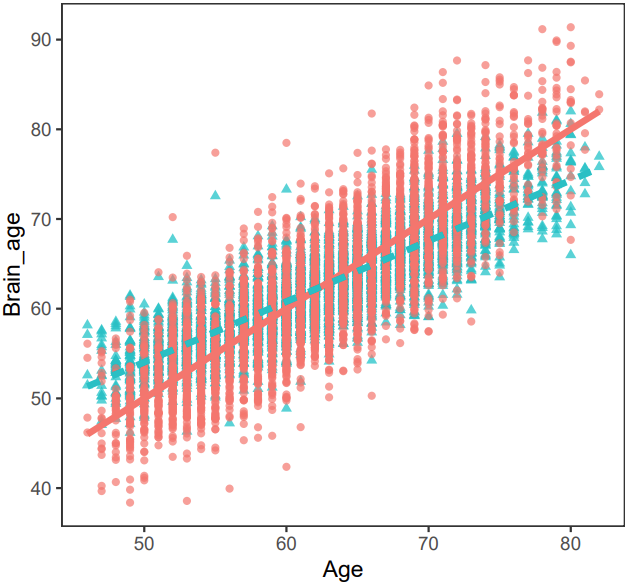

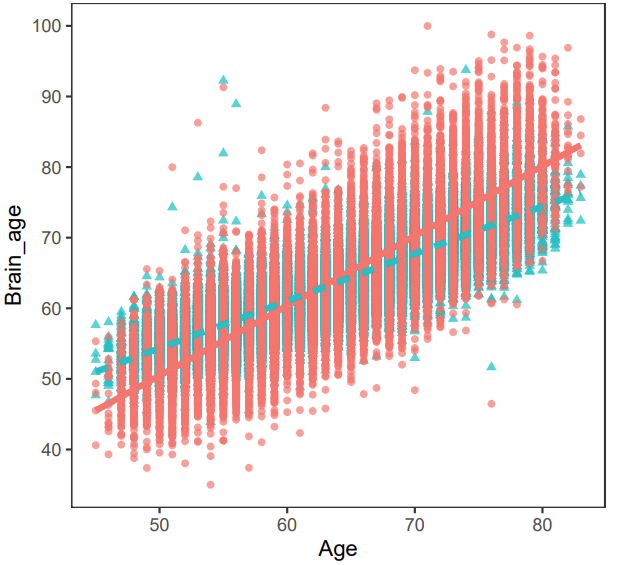

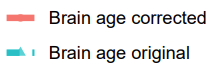


A

A

**sFigure 4 Original and corrected brain age as a function of chronological age in the (A) training set and (B) test set.**

Reference

1. Arold D, Bornstein SR, Perakakis N, Ehrlich S, Bernardoni F: **Regional gray matter changes in steatotic liver disease provide a neurobiological link to depression: A cross-sectional UK Biobank cohort study**. *Metabolism: clinical and experimental* 2024, **159**:155983.

2. Beheshti I, Nugent S, Potvin O, Duchesne S: **Bias-adjustment in neuroimaging-based brain age frameworks: A robust scheme**. *Neuroimage Clin* 2019, **24**:102063.

3. Huang H, Wang J, Dunk MM, Guo J, Dove A, Ma J, Bennett DA, Xu W: **Association of Cardiovascular Health With Brain Age Estimated Using Machine Learning Methods in Middle-Aged and Older Adults**. *Neurology* 2024, **103**(2):e209530.

4. Dove A, Wang J, Huang H, Dunk MM, Sakakibara S, Guitart-Masip M, Papenberg G, Xu W: **Diabetes, Prediabetes, and Brain Aging: The Role of Healthy Lifestyle**. *Diabetes care* 2024, **47**(10):1794-1802.

**sFigure 5 Joint effects of PDFF, cT1 and sex on brain-PAD.**

Abbreviations: SE, standard error; PDFF, proton density fat fraction; cT1, iron-corrected T1 mapping.

Model was simultaneous model combining PDFF and cT1 with further adjustments for age, location of assessment center, ethnic background, education level, Townsend deprivation index, smoking status, alcohol intake, physical activity, social connection, cardiometabolic burden, and BMI.

**sFigure 6** **Joint effects of PDFF, cT1 and cardiometabolic burden on brain-PAD****.**

Abbreviations: SE, standard error; PDFF, proton density fat fraction; cT1, iron-corrected T1 mapping.

Model was simultaneous model combining PDFF and cT1 with further adjustments for age, sex, location of assessment center, ethnic background, education level, Townsend deprivation index, smoking status, alcohol intake, physical activity, social connection, and BMI.

**
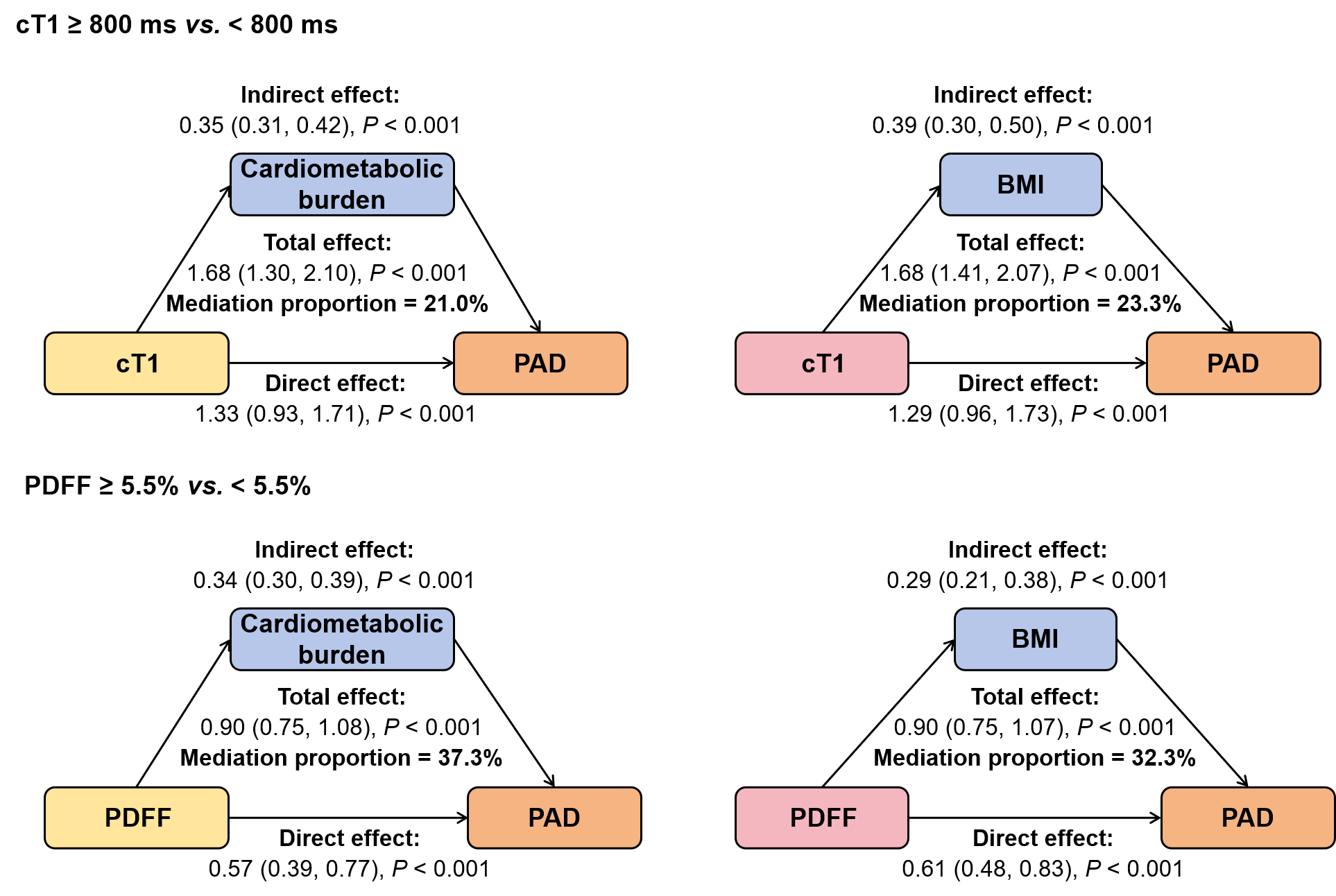
sFigure 7 Mediation effects of BMI and cardiometabolic burden on the associations of PDFF and cT1 with brain-PAD.**

Abbreviations: PDFF, proton density fat fraction; cT1, iron-corrected T1 mapping.

Model was simultaneous model combining PDFF and cT1 with further adjustments for age, sex, location of assessment center, ethnic background, education level, Townsend deprivation index, smoking status, alcohol intake, physical activity, social connection, and BMI.
