# Supplementary material for: The role of healthy lifestyle in the association between hepatic fibro-inflammation and steatosis and brain aging—a cross-sectional study
Source: Front Aging Neurosci. 2026 Mar 16;18:1801577. doi: 10.3389/fnagi.2026.1801577 (PMC13033754; doi:10.3389/fnagi.2026.1801577)
Supplement: Supplementary file 2 [file Data_Sheet_2.docx]

| **sTable1 Miss count of imaging derived phenotypes** |  |
| --- | --- |
| **IDPs** | **Missing** |
| Volumetric scaling from T1 head image to standard space | 4 |
| Volume of peripheral cortical grey matter (normalised for head size) | 4 |
| Volume of peripheral cortical grey matter | 4 |
| Volume of ventricular cerebrospinal fluid | 4 |
| Volume of ventricular cerebrospinal fluid (normalised for head size) | 4 |
| Volume of grey matter (normalised for head size) | 4 |
| Volume of grey matter | 4 |
| Volume of white matter | 4 |
| Volume of white matter (normalised for head size) | 4 |
| Volume of brain grey+white matter | 4 |
| Volume of brain grey+white matter (normalised for head size) | 4 |
| Volume of thalamus (left) | 4 |
| Volume of thalamus (right) | 4 |
| Volume of caudate (left) | 4 |
| Volume of caudate (right) | 4 |
| Volume of putamen (left) | 4 |
| Volume of putamen (right) | 4 |
| Volume of pallidum (left) | 4 |
| Volume of pallidum (right) | 4 |
| Volume of hippocampus (left) | 4 |
| Volume of hippocampus (right) | 4 |
| Volume of amygdala (left) | 4 |
| Volume of amygdala (right) | 4 |
| Volume of accumbens (left) | 4 |
| Volume of accumbens (right) | 4 |
| Volume of brain stem + 4th ventricle | 4 |
| Volume of grey matter in Frontal Pole (left) | 4 |
| Volume of grey matter in Frontal Pole (right) | 4 |
| Volume of grey matter in Insular Cortex (left) | 4 |
| Volume of grey matter in Insular Cortex (right) | 4 |
| Volume of grey matter in Superior Frontal Gyrus (left) | 4 |
| Volume of grey matter in Superior Frontal Gyrus (right) | 4 |
| Volume of grey matter in Middle Frontal Gyrus (left) | 4 |
| Volume of grey matter in Middle Frontal Gyrus (right) | 4 |
| Volume of grey matter in Inferior Frontal Gyrus pars triangularis (left) | 4 |
| Volume of grey matter in Inferior Frontal Gyrus pars triangularis (right) | 4 |
| Volume of grey matter in Inferior Frontal Gyrus pars opercularis (left) | 4 |
| Volume of grey matter in Inferior Frontal Gyrus pars opercularis (right) | 4 |
| Volume of grey matter in Precentral Gyrus (left) | 4 |
| Volume of grey matter in Precentral Gyrus (right) | 4 |
| Volume of grey matter in Temporal Pole (left) | 4 |
| Volume of grey matter in Temporal Pole (right) | 4 |
| Volume of grey matter in Superior Temporal Gyrus anterior division (left) | 4 |
| Volume of grey matter in Superior Temporal Gyrus anterior division (right) | 4 |
| Volume of grey matter in Superior Temporal Gyrus posterior division (left) | 4 |
| Volume of grey matter in Superior Temporal Gyrus posterior division (right) | 4 |
| Volume of grey matter in Middle Temporal Gyrus anterior division (left) | 4 |
| Volume of grey matter in Middle Temporal Gyrus anterior division (right) | 4 |
| Volume of grey matter in Middle Temporal Gyrus posterior division (left) | 4 |
| Volume of grey matter in Middle Temporal Gyrus posterior division (right) | 4 |
| Volume of grey matter in Middle Temporal Gyrus temporooccipital part (left) | 4 |
| Volume of grey matter in Middle Temporal Gyrus temporooccipital part (right) | 4 |
| Volume of grey matter in Inferior Temporal Gyrus anterior division (left) | 4 |
| Volume of grey matter in Inferior Temporal Gyrus anterior division (right) | 4 |
| Volume of grey matter in Inferior Temporal Gyrus posterior division (left) | 4 |
| Volume of grey matter in Inferior Temporal Gyrus posterior division (right) | 4 |
| Volume of grey matter in Inferior Temporal Gyrus temporooccipital part (left) | 4 |
| Volume of grey matter in Inferior Temporal Gyrus temporooccipital part (right) | 4 |
| Volume of grey matter in Postcentral Gyrus (left) | 4 |
| Volume of grey matter in Postcentral Gyrus (right) | 4 |
| Volume of grey matter in Superior Parietal Lobule (left) | 4 |
| Volume of grey matter in Superior Parietal Lobule (right) | 4 |
| Volume of grey matter in Supramarginal Gyrus anterior division (left) | 4 |
| Volume of grey matter in Supramarginal Gyrus anterior division (right) | 4 |
| Volume of grey matter in Supramarginal Gyrus posterior division (left) | 4 |
| Volume of grey matter in Supramarginal Gyrus posterior division (right) | 4 |
| Volume of grey matter in Angular Gyrus (left) | 4 |
| Volume of grey matter in Angular Gyrus (right) | 4 |
| Volume of grey matter in Lateral Occipital Cortex superior division (left) | 4 |
| Volume of grey matter in Lateral Occipital Cortex superior division (right) | 4 |
| Volume of grey matter in Lateral Occipital Cortex inferior division (left) | 4 |
| Volume of grey matter in Lateral Occipital Cortex inferior division (right) | 4 |
| Volume of grey matter in Intracalcarine Cortex (left) | 4 |
| Volume of grey matter in Intracalcarine Cortex (right) | 4 |
| Volume of grey matter in Frontal Medial Cortex (left) | 4 |
| Volume of grey matter in Frontal Medial Cortex (right) | 4 |
| Volume of grey matter in Juxtapositional Lobule Cortex (formerly Supplementary Motor Cortex) (left) | 4 |
| Volume of grey matter in Juxtapositional Lobule Cortex (formerly Supplementary Motor Cortex) (right) | 4 |
| Volume of grey matter in Subcallosal Cortex (left) | 4 |
| Volume of grey matter in Subcallosal Cortex (right) | 4 |
| Volume of grey matter in Paracingulate Gyrus (left) | 4 |
| Volume of grey matter in Paracingulate Gyrus (right) | 4 |
| Volume of grey matter in Cingulate Gyrus anterior division (left) | 4 |
| Volume of grey matter in Cingulate Gyrus anterior division (right) | 4 |
| Volume of grey matter in Cingulate Gyrus posterior division (left) | 4 |
| Volume of grey matter in Cingulate Gyrus posterior division (right) | 4 |
| Volume of grey matter in Precuneous Cortex (left) | 4 |
| Volume of grey matter in Precuneous Cortex (right) | 4 |
| Volume of grey matter in Cuneal Cortex (left) | 4 |
| Volume of grey matter in Cuneal Cortex (right) | 4 |
| Volume of grey matter in Frontal Orbital Cortex (left) | 4 |
| Volume of grey matter in Frontal Orbital Cortex (right) | 4 |
| Volume of grey matter in Parahippocampal Gyrus anterior division (left) | 4 |
| Volume of grey matter in Parahippocampal Gyrus anterior division (right) | 4 |
| Volume of grey matter in Parahippocampal Gyrus posterior division (left) | 4 |
| Volume of grey matter in Parahippocampal Gyrus posterior division (right) | 4 |
| Volume of grey matter in Lingual Gyrus (left) | 4 |
| Volume of grey matter in Lingual Gyrus (right) | 4 |
| Volume of grey matter in Temporal Fusiform Cortex anterior division (left) | 4 |
| Volume of grey matter in Temporal Fusiform Cortex anterior division (right) | 4 |
| Volume of grey matter in Temporal Fusiform Cortex posterior division (left) | 4 |
| Volume of grey matter in Temporal Fusiform Cortex posterior division (right) | 4 |
| Volume of grey matter in Temporal Occipital Fusiform Cortex (left) | 4 |
| Volume of grey matter in Temporal Occipital Fusiform Cortex (right) | 4 |
| Volume of grey matter in Occipital Fusiform Gyrus (left) | 4 |
| Volume of grey matter in Occipital Fusiform Gyrus (right) | 4 |
| Volume of grey matter in Frontal Operculum Cortex (left) | 4 |
| Volume of grey matter in Frontal Operculum Cortex (right) | 4 |
| Volume of grey matter in Central Opercular Cortex (left) | 4 |
| Volume of grey matter in Central Opercular Cortex (right) | 4 |
| Volume of grey matter in Parietal Operculum Cortex (left) | 4 |
| Volume of grey matter in Parietal Operculum Cortex (right) | 4 |
| Volume of grey matter in Planum Polare (left) | 4 |
| Volume of grey matter in Planum Polare (right) | 4 |
| Volume of grey matter in Heschl's Gyrus (includes H1 and H2) (left) | 4 |
| Volume of grey matter in Heschl's Gyrus (includes H1 and H2) (right) | 4 |
| Volume of grey matter in Planum Temporale (left) | 4 |
| Volume of grey matter in Planum Temporale (right) | 4 |
| Volume of grey matter in Supracalcarine Cortex (left) | 4 |
| Volume of grey matter in Supracalcarine Cortex (right) | 4 |
| Volume of grey matter in Occipital Pole (left) | 4 |
| Volume of grey matter in Occipital Pole (right) | 4 |
| Volume of grey matter in Thalamus (left) | 4 |
| Volume of grey matter in Thalamus (right) | 4 |
| Volume of grey matter in Caudate (left) | 4 |
| Volume of grey matter in Caudate (right) | 4 |
| Volume of grey matter in Putamen (left) | 4 |
| Volume of grey matter in Putamen (right) | 4 |
| Volume of grey matter in Pallidum (left) | 4 |
| Volume of grey matter in Pallidum (right) | 4 |
| Volume of grey matter in Hippocampus (left) | 4 |
| Volume of grey matter in Hippocampus (right) | 4 |
| Volume of grey matter in Amygdala (left) | 4 |
| Volume of grey matter in Amygdala (right) | 4 |
| Volume of grey matter in Ventral Striatum (left) | 4 |
| Volume of grey matter in Ventral Striatum (right) | 4 |
| Volume of grey matter in Brain-Stem | 4 |
| Volume of grey matter in I-IV Cerebellum (left) | 4 |
| Volume of grey matter in I-IV Cerebellum (right) | 4 |
| Volume of grey matter in V Cerebellum (left) | 4 |
| Volume of grey matter in V Cerebellum (right) | 4 |
| Volume of grey matter in VI Cerebellum (left) | 4 |
| Volume of grey matter in VI Cerebellum (right) | 4 |
| Volume of grey matter in VI Cerebellum (vermis) | 4 |
| Volume of grey matter in Crus I Cerebellum (left) | 4 |
| Volume of grey matter in Crus I Cerebellum (right) | 4 |
| Volume of grey matter in Crus I Cerebellum (vermis) | 4 |
| Volume of grey matter in Crus II Cerebellum (left) | 4 |
| Volume of grey matter in Crus II Cerebellum (right) | 4 |
| Volume of grey matter in Crus II Cerebellum (vermis) | 4 |
| Volume of grey matter in VIIb Cerebellum (left) | 4 |
| Volume of grey matter in VIIb Cerebellum (right) | 4 |
| Volume of grey matter in VIIb Cerebellum (vermis) | 4 |
| Volume of grey matter in VIIIa Cerebellum (left) | 4 |
| Volume of grey matter in VIIIa Cerebellum (right) | 4 |
| Volume of grey matter in VIIIa Cerebellum (vermis) | 4 |
| Volume of grey matter in VIIIb Cerebellum (left) | 4 |
| Volume of grey matter in VIIIb Cerebellum (right) | 4 |
| Volume of grey matter in VIIIb Cerebellum (vermis) | 4 |
| Volume of grey matter in IX Cerebellum (left) | 4 |
| Volume of grey matter in IX Cerebellum (right) | 4 |
| Volume of grey matter in IX Cerebellum (vermis) | 4 |
| Volume of grey matter in X Cerebellum (left) | 4 |
| Volume of grey matter in X Cerebellum (vermis) | 4 |
| Volume of grey matter in X Cerebellum (right) | 4 |
| Total volume of white matter hyperintensities (from T1 and T2 FLAIR images) | 605 |
| Median T2star in thalamus (left) | 2601 |
| Median T2star in thalamus (right) | 2601 |
| Median T2star in caudate (left) | 2601 |
| Median T2star in caudate (right) | 2601 |
| Median T2star in putamen (left) | 2601 |
| Median T2star in putamen (right) | 2601 |
| Median T2star in pallidum (left) | 2601 |
| Median T2star in pallidum (right) | 2601 |
| Median T2star in hippocampus (left) | 2601 |
| Median T2star in hippocampus (right) | 2601 |
| Median T2star in amygdala (left) | 2601 |
| Median T2star in amygdala (right) | 2601 |
| Median T2star in accumbens (left) | 2601 |
| Median T2star in accumbens (right) | 2601 |
| Mean FA in middle cerebellar peduncle on FA skeleton | 528 |
| Mean FA in pontine crossing tract on FA skeleton | 528 |
| Mean FA in genu of corpus callosum on FA skeleton | 528 |
| Mean FA in body of corpus callosum on FA skeleton | 528 |
| Mean FA in splenium of corpus callosum on FA skeleton | 528 |
| Mean FA in fornix on FA skeleton | 528 |
| Mean FA in corticospinal tract on FA skeleton (left) | 528 |
| Mean FA in corticospinal tract on FA skeleton (right) | 528 |
| Mean FA in medial lemniscus on FA skeleton (left) | 528 |
| Mean FA in medial lemniscus on FA skeleton (right) | 528 |
| Mean FA in inferior cerebellar peduncle on FA skeleton (left) | 528 |
| Mean FA in inferior cerebellar peduncle on FA skeleton (right) | 528 |
| Mean FA in superior cerebellar peduncle on FA skeleton (left) | 528 |
| Mean FA in superior cerebellar peduncle on FA skeleton (right) | 528 |
| Mean FA in cerebral peduncle on FA skeleton (left) | 528 |
| Mean FA in cerebral peduncle on FA skeleton (right) | 528 |
| Mean FA in anterior limb of internal capsule on FA skeleton (left) | 528 |
| Mean FA in anterior limb of internal capsule on FA skeleton (right) | 528 |
| Mean FA in posterior limb of internal capsule on FA skeleton (left) | 528 |
| Mean FA in posterior limb of internal capsule on FA skeleton (right) | 528 |
| Mean FA in retrolenticular part of internal capsule on FA skeleton (left) | 528 |
| Mean FA in retrolenticular part of internal capsule on FA skeleton (right) | 528 |
| Mean FA in anterior corona radiata on FA skeleton (left) | 528 |
| Mean FA in anterior corona radiata on FA skeleton (right) | 528 |
| Mean FA in superior corona radiata on FA skeleton (left) | 528 |
| Mean FA in superior corona radiata on FA skeleton (right) | 528 |
| Mean FA in posterior corona radiata on FA skeleton (left) | 528 |
| Mean FA in posterior corona radiata on FA skeleton (right) | 528 |
| Mean FA in posterior thalamic radiation on FA skeleton (left) | 528 |
| Mean FA in posterior thalamic radiation on FA skeleton (right) | 528 |
| Mean FA in sagittal stratum on FA skeleton (left) | 528 |
| Mean FA in sagittal stratum on FA skeleton (right) | 528 |
| Mean FA in external capsule on FA skeleton (left) | 528 |
| Mean FA in external capsule on FA skeleton (right) | 528 |
| Mean FA in cingulum cingulate gyrus on FA skeleton (left) | 528 |
| Mean FA in cingulum cingulate gyrus on FA skeleton (right) | 528 |
| Mean FA in cingulum hippocampus on FA skeleton (left) | 528 |
| Mean FA in cingulum hippocampus on FA skeleton (right) | 528 |
| Mean FA in fornix cres+stria terminalis on FA skeleton (left) | 528 |
| Mean FA in fornix cres+stria terminalis on FA skeleton (right) | 528 |
| Mean FA in superior longitudinal fasciculus on FA skeleton (left) | 528 |
| Mean FA in superior longitudinal fasciculus on FA skeleton (right) | 528 |
| Mean FA in superior fronto-occipital fasciculus on FA skeleton (left) | 528 |
| Mean FA in superior fronto-occipital fasciculus on FA skeleton (right) | 528 |
| Mean FA in uncinate fasciculus on FA skeleton (left) | 528 |
| Mean FA in uncinate fasciculus on FA skeleton (right) | 528 |
| Mean FA in tapetum on FA skeleton (left) | 528 |
| Mean FA in tapetum on FA skeleton (right) | 528 |
| Mean MD in middle cerebellar peduncle on FA skeleton | 528 |
| Mean MD in pontine crossing tract on FA skeleton | 528 |
| Mean MD in genu of corpus callosum on FA skeleton | 528 |
| Mean MD in body of corpus callosum on FA skeleton | 528 |
| Mean MD in splenium of corpus callosum on FA skeleton | 528 |
| Mean MD in fornix on FA skeleton | 528 |
| Mean MD in corticospinal tract on FA skeleton (left) | 528 |
| Mean MD in corticospinal tract on FA skeleton (right) | 528 |
| Mean MD in medial lemniscus on FA skeleton (left) | 528 |
| Mean MD in medial lemniscus on FA skeleton (right) | 528 |
| Mean MD in inferior cerebellar peduncle on FA skeleton (left) | 528 |
| Mean MD in inferior cerebellar peduncle on FA skeleton (right) | 528 |
| Mean MD in superior cerebellar peduncle on FA skeleton (left) | 528 |
| Mean MD in superior cerebellar peduncle on FA skeleton (right) | 528 |
| Mean MD in cerebral peduncle on FA skeleton (left) | 528 |
| Mean MD in cerebral peduncle on FA skeleton (right) | 528 |
| Mean MD in anterior limb of internal capsule on FA skeleton (left) | 528 |
| Mean MD in anterior limb of internal capsule on FA skeleton (right) | 528 |
| Mean MD in posterior limb of internal capsule on FA skeleton (left) | 528 |
| Mean MD in posterior limb of internal capsule on FA skeleton (right) | 528 |
| Mean MD in retrolenticular part of internal capsule on FA skeleton (left) | 528 |
| Mean MD in retrolenticular part of internal capsule on FA skeleton (right) | 528 |
| Mean MD in anterior corona radiata on FA skeleton (left) | 528 |
| Mean MD in anterior corona radiata on FA skeleton (right) | 528 |
| Mean MD in superior corona radiata on FA skeleton (left) | 528 |
| Mean MD in superior corona radiata on FA skeleton (right) | 528 |
| Mean MD in posterior corona radiata on FA skeleton (left) | 528 |
| Mean MD in posterior corona radiata on FA skeleton (right) | 528 |
| Mean MD in posterior thalamic radiation on FA skeleton (left) | 528 |
| Mean MD in posterior thalamic radiation on FA skeleton (right) | 528 |
| Mean MD in sagittal stratum on FA skeleton (left) | 528 |
| Mean MD in sagittal stratum on FA skeleton (right) | 528 |
| Mean MD in external capsule on FA skeleton (left) | 528 |
| Mean MD in external capsule on FA skeleton (right) | 528 |
| Mean MD in cingulum cingulate gyrus on FA skeleton (left) | 528 |
| Mean MD in cingulum cingulate gyrus on FA skeleton (right) | 528 |
| Mean MD in cingulum hippocampus on FA skeleton (left) | 528 |
| Mean MD in cingulum hippocampus on FA skeleton (right) | 528 |
| Mean MD in fornix cres+stria terminalis on FA skeleton (left) | 528 |
| Mean MD in fornix cres+stria terminalis on FA skeleton (right) | 528 |
| Mean MD in superior longitudinal fasciculus on FA skeleton (left) | 528 |
| Mean MD in superior longitudinal fasciculus on FA skeleton (right) | 528 |
| Mean MD in superior fronto-occipital fasciculus on FA skeleton (left) | 528 |
| Mean MD in superior fronto-occipital fasciculus on FA skeleton (right) | 528 |
| Mean MD in uncinate fasciculus on FA skeleton (left) | 528 |
| Mean MD in uncinate fasciculus on FA skeleton (right) | 528 |
| Mean MD in tapetum on FA skeleton (left) | 528 |
| Mean MD in tapetum on FA skeleton (right) | 528 |
| Mean MO in middle cerebellar peduncle on FA skeleton | 528 |
| Mean MO in pontine crossing tract on FA skeleton | 528 |
| Mean MO in genu of corpus callosum on FA skeleton | 528 |
| Mean MO in body of corpus callosum on FA skeleton | 528 |
| Mean MO in splenium of corpus callosum on FA skeleton | 528 |
| Mean MO in fornix on FA skeleton | 528 |
| Mean MO in corticospinal tract on FA skeleton (left) | 528 |
| Mean MO in corticospinal tract on FA skeleton (right) | 528 |
| Mean MO in medial lemniscus on FA skeleton (left) | 528 |
| Mean MO in medial lemniscus on FA skeleton (right) | 528 |
| Mean MO in inferior cerebellar peduncle on FA skeleton (left) | 528 |
| Mean MO in inferior cerebellar peduncle on FA skeleton (right) | 528 |
| Mean MO in superior cerebellar peduncle on FA skeleton (left) | 528 |
| Mean MO in superior cerebellar peduncle on FA skeleton (right) | 528 |
| Mean MO in cerebral peduncle on FA skeleton (left) | 528 |
| Mean MO in cerebral peduncle on FA skeleton (right) | 528 |
| Mean MO in anterior limb of internal capsule on FA skeleton (left) | 528 |
| Mean MO in anterior limb of internal capsule on FA skeleton (right) | 528 |
| Mean MO in posterior limb of internal capsule on FA skeleton (left) | 528 |
| Mean MO in posterior limb of internal capsule on FA skeleton (right) | 528 |
| Mean MO in retrolenticular part of internal capsule on FA skeleton (left) | 528 |
| Mean MO in retrolenticular part of internal capsule on FA skeleton (right) | 528 |
| Mean MO in anterior corona radiata on FA skeleton (left) | 528 |
| Mean MO in anterior corona radiata on FA skeleton (right) | 528 |
| Mean MO in superior corona radiata on FA skeleton (left) | 528 |
| Mean MO in superior corona radiata on FA skeleton (right) | 528 |
| Mean MO in posterior corona radiata on FA skeleton (left) | 528 |
| Mean MO in posterior corona radiata on FA skeleton (right) | 528 |
| Mean MO in posterior thalamic radiation on FA skeleton (left) | 528 |
| Mean MO in posterior thalamic radiation on FA skeleton (right) | 528 |
| Mean MO in sagittal stratum on FA skeleton (left) | 528 |
| Mean MO in sagittal stratum on FA skeleton (right) | 528 |
| Mean MO in external capsule on FA skeleton (left) | 528 |
| Mean MO in external capsule on FA skeleton (right) | 528 |
| Mean MO in cingulum cingulate gyrus on FA skeleton (left) | 528 |
| Mean MO in cingulum cingulate gyrus on FA skeleton (right) | 528 |
| Mean MO in cingulum hippocampus on FA skeleton (left) | 528 |
| Mean MO in cingulum hippocampus on FA skeleton (right) | 528 |
| Mean MO in fornix cres+stria terminalis on FA skeleton (left) | 528 |
| Mean MO in fornix cres+stria terminalis on FA skeleton (right) | 528 |
| Mean MO in superior longitudinal fasciculus on FA skeleton (left) | 528 |
| Mean MO in superior longitudinal fasciculus on FA skeleton (right) | 528 |
| Mean MO in superior fronto-occipital fasciculus on FA skeleton (left) | 528 |
| Mean MO in superior fronto-occipital fasciculus on FA skeleton (right) | 528 |
| Mean MO in uncinate fasciculus on FA skeleton (left) | 528 |
| Mean MO in uncinate fasciculus on FA skeleton (right) | 528 |
| Mean MO in tapetum on FA skeleton (left) | 528 |
| Mean MO in tapetum on FA skeleton (right) | 528 |
| Mean L1 in middle cerebellar peduncle on FA skeleton | 528 |
| Mean L1 in pontine crossing tract on FA skeleton | 528 |
| Mean L1 in genu of corpus callosum on FA skeleton | 528 |
| Mean L1 in body of corpus callosum on FA skeleton | 528 |
| Mean L1 in splenium of corpus callosum on FA skeleton | 528 |
| Mean L1 in fornix on FA skeleton | 528 |
| Mean L1 in corticospinal tract on FA skeleton (left) | 528 |
| Mean L1 in corticospinal tract on FA skeleton (right) | 528 |
| Mean L1 in medial lemniscus on FA skeleton (left) | 528 |
| Mean L1 in medial lemniscus on FA skeleton (right) | 528 |
| Mean L1 in inferior cerebellar peduncle on FA skeleton (left) | 528 |
| Mean L1 in inferior cerebellar peduncle on FA skeleton (right) | 528 |
| Mean L1 in superior cerebellar peduncle on FA skeleton (left) | 528 |
| Mean L1 in superior cerebellar peduncle on FA skeleton (right) | 528 |
| Mean L1 in cerebral peduncle on FA skeleton (left) | 528 |
| Mean L1 in cerebral peduncle on FA skeleton (right) | 528 |
| Mean L1 in anterior limb of internal capsule on FA skeleton (left) | 528 |
| Mean L1 in anterior limb of internal capsule on FA skeleton (right) | 528 |
| Mean L1 in posterior limb of internal capsule on FA skeleton (left) | 528 |
| Mean L1 in posterior limb of internal capsule on FA skeleton (right) | 528 |
| Mean L1 in retrolenticular part of internal capsule on FA skeleton (left) | 528 |
| Mean L1 in retrolenticular part of internal capsule on FA skeleton (right) | 528 |
| Mean L1 in anterior corona radiata on FA skeleton (left) | 528 |
| Mean L1 in anterior corona radiata on FA skeleton (right) | 528 |
| Mean L1 in superior corona radiata on FA skeleton (left) | 528 |
| Mean L1 in superior corona radiata on FA skeleton (right) | 528 |
| Mean L1 in posterior corona radiata on FA skeleton (left) | 528 |
| Mean L1 in posterior corona radiata on FA skeleton (right) | 528 |
| Mean L1 in posterior thalamic radiation on FA skeleton (left) | 528 |
| Mean L1 in posterior thalamic radiation on FA skeleton (right) | 528 |
| Mean L1 in sagittal stratum on FA skeleton (left) | 528 |
| Mean L1 in sagittal stratum on FA skeleton (right) | 528 |
| Mean L1 in external capsule on FA skeleton (left) | 528 |
| Mean L1 in external capsule on FA skeleton (right) | 528 |
| Mean L1 in cingulum cingulate gyrus on FA skeleton (left) | 528 |
| Mean L1 in cingulum cingulate gyrus on FA skeleton (right) | 528 |
| Mean L1 in cingulum hippocampus on FA skeleton (left) | 528 |
| Mean L1 in cingulum hippocampus on FA skeleton (right) | 528 |
| Mean L1 in fornix cres+stria terminalis on FA skeleton (left) | 528 |
| Mean L1 in fornix cres+stria terminalis on FA skeleton (right) | 528 |
| Mean L1 in superior longitudinal fasciculus on FA skeleton (left) | 528 |
| Mean L1 in superior longitudinal fasciculus on FA skeleton (right) | 528 |
| Mean L1 in superior fronto-occipital fasciculus on FA skeleton (left) | 528 |
| Mean L1 in superior fronto-occipital fasciculus on FA skeleton (right) | 528 |
| Mean L1 in uncinate fasciculus on FA skeleton (left) | 528 |
| Mean L1 in uncinate fasciculus on FA skeleton (right) | 528 |
| Mean L1 in tapetum on FA skeleton (left) | 528 |
| Mean L1 in tapetum on FA skeleton (right) | 528 |
| Mean L2 in middle cerebellar peduncle on FA skeleton | 528 |
| Mean L2 in pontine crossing tract on FA skeleton | 528 |
| Mean L2 in genu of corpus callosum on FA skeleton | 528 |
| Mean L2 in body of corpus callosum on FA skeleton | 528 |
| Mean L2 in splenium of corpus callosum on FA skeleton | 528 |
| Mean L2 in fornix on FA skeleton | 528 |
| Mean L2 in corticospinal tract on FA skeleton (left) | 528 |
| Mean L2 in corticospinal tract on FA skeleton (right) | 528 |
| Mean L2 in medial lemniscus on FA skeleton (left) | 528 |
| Mean L2 in medial lemniscus on FA skeleton (right) | 528 |
| Mean L2 in inferior cerebellar peduncle on FA skeleton (left) | 528 |
| Mean L2 in inferior cerebellar peduncle on FA skeleton (right) | 528 |
| Mean L2 in superior cerebellar peduncle on FA skeleton (left) | 528 |
| Mean L2 in superior cerebellar peduncle on FA skeleton (right) | 528 |
| Mean L2 in cerebral peduncle on FA skeleton (left) | 528 |
| Mean L2 in cerebral peduncle on FA skeleton (right) | 528 |
| Mean L2 in anterior limb of internal capsule on FA skeleton (left) | 528 |
| Mean L2 in anterior limb of internal capsule on FA skeleton (right) | 528 |
| Mean L2 in posterior limb of internal capsule on FA skeleton (left) | 528 |
| Mean L2 in posterior limb of internal capsule on FA skeleton (right) | 528 |
| Mean L2 in retrolenticular part of internal capsule on FA skeleton (left) | 528 |
| Mean L2 in retrolenticular part of internal capsule on FA skeleton (right) | 528 |
| Mean L2 in anterior corona radiata on FA skeleton (left) | 528 |
| Mean L2 in anterior corona radiata on FA skeleton (right) | 528 |
| Mean L2 in superior corona radiata on FA skeleton (left) | 528 |
| Mean L2 in superior corona radiata on FA skeleton (right) | 528 |
| Mean L2 in posterior corona radiata on FA skeleton (left) | 528 |
| Mean L2 in posterior corona radiata on FA skeleton (right) | 528 |
| Mean L2 in posterior thalamic radiation on FA skeleton (left) | 528 |
| Mean L2 in posterior thalamic radiation on FA skeleton (right) | 528 |
| Mean L2 in sagittal stratum on FA skeleton (left) | 528 |
| Mean L2 in sagittal stratum on FA skeleton (right) | 528 |
| Mean L2 in external capsule on FA skeleton (left) | 528 |
| Mean L2 in external capsule on FA skeleton (right) | 528 |
| Mean L2 in cingulum cingulate gyrus on FA skeleton (left) | 528 |
| Mean L2 in cingulum cingulate gyrus on FA skeleton (right) | 528 |
| Mean L2 in cingulum hippocampus on FA skeleton (left) | 528 |
| Mean L2 in cingulum hippocampus on FA skeleton (right) | 528 |
| Mean L2 in fornix cres+stria terminalis on FA skeleton (left) | 528 |
| Mean L2 in fornix cres+stria terminalis on FA skeleton (right) | 528 |
| Mean L2 in superior longitudinal fasciculus on FA skeleton (left) | 528 |
| Mean L2 in superior longitudinal fasciculus on FA skeleton (right) | 528 |
| Mean L2 in superior fronto-occipital fasciculus on FA skeleton (left) | 528 |
| Mean L2 in superior fronto-occipital fasciculus on FA skeleton (right) | 528 |
| Mean L2 in uncinate fasciculus on FA skeleton (left) | 528 |
| Mean L2 in uncinate fasciculus on FA skeleton (right) | 528 |
| Mean L2 in tapetum on FA skeleton (left) | 528 |
| Mean L2 in tapetum on FA skeleton (right) | 528 |
| Mean L3 in middle cerebellar peduncle on FA skeleton | 528 |
| Mean L3 in pontine crossing tract on FA skeleton | 528 |
| Mean L3 in genu of corpus callosum on FA skeleton | 528 |
| Mean L3 in body of corpus callosum on FA skeleton | 528 |
| Mean L3 in splenium of corpus callosum on FA skeleton | 528 |
| Mean L3 in fornix on FA skeleton | 528 |
| Mean L3 in corticospinal tract on FA skeleton (left) | 528 |
| Mean L3 in corticospinal tract on FA skeleton (right) | 528 |
| Mean L3 in medial lemniscus on FA skeleton (left) | 528 |
| Mean L3 in medial lemniscus on FA skeleton (right) | 528 |
| Mean L3 in inferior cerebellar peduncle on FA skeleton (left) | 528 |
| Mean L3 in inferior cerebellar peduncle on FA skeleton (right) | 528 |
| Mean L3 in superior cerebellar peduncle on FA skeleton (left) | 528 |
| Mean L3 in superior cerebellar peduncle on FA skeleton (right) | 528 |
| Mean L3 in cerebral peduncle on FA skeleton (left) | 528 |
| Mean L3 in cerebral peduncle on FA skeleton (right) | 528 |
| Mean L3 in anterior limb of internal capsule on FA skeleton (left) | 528 |
| Mean L3 in anterior limb of internal capsule on FA skeleton (right) | 528 |
| Mean L3 in posterior limb of internal capsule on FA skeleton (left) | 528 |
| Mean L3 in posterior limb of internal capsule on FA skeleton (right) | 528 |
| Mean L3 in retrolenticular part of internal capsule on FA skeleton (left) | 528 |
| Mean L3 in retrolenticular part of internal capsule on FA skeleton (right) | 528 |
| Mean L3 in anterior corona radiata on FA skeleton (left) | 528 |
| Mean L3 in anterior corona radiata on FA skeleton (right) | 528 |
| Mean L3 in superior corona radiata on FA skeleton (left) | 528 |
| Mean L3 in superior corona radiata on FA skeleton (right) | 528 |
| Mean L3 in posterior corona radiata on FA skeleton (left) | 528 |
| Mean L3 in posterior corona radiata on FA skeleton (right) | 528 |
| Mean L3 in posterior thalamic radiation on FA skeleton (left) | 528 |
| Mean L3 in posterior thalamic radiation on FA skeleton (right) | 528 |
| Mean L3 in sagittal stratum on FA skeleton (left) | 528 |
| Mean L3 in sagittal stratum on FA skeleton (right) | 528 |
| Mean L3 in external capsule on FA skeleton (left) | 528 |
| Mean L3 in external capsule on FA skeleton (right) | 528 |
| Mean L3 in cingulum cingulate gyrus on FA skeleton (left) | 528 |
| Mean L3 in cingulum cingulate gyrus on FA skeleton (right) | 528 |
| Mean L3 in cingulum hippocampus on FA skeleton (left) | 528 |
| Mean L3 in cingulum hippocampus on FA skeleton (right) | 528 |
| Mean L3 in fornix cres+stria terminalis on FA skeleton (left) | 528 |
| Mean L3 in fornix cres+stria terminalis on FA skeleton (right) | 528 |
| Mean L3 in superior longitudinal fasciculus on FA skeleton (left) | 528 |
| Mean L3 in superior longitudinal fasciculus on FA skeleton (right) | 528 |
| Mean L3 in superior fronto-occipital fasciculus on FA skeleton (left) | 528 |
| Mean L3 in superior fronto-occipital fasciculus on FA skeleton (right) | 528 |
| Mean L3 in uncinate fasciculus on FA skeleton (left) | 528 |
| Mean L3 in uncinate fasciculus on FA skeleton (right) | 528 |
| Mean L3 in tapetum on FA skeleton (left) | 528 |
| Mean L3 in tapetum on FA skeleton (right) | 528 |
| Mean ICVF in middle cerebellar peduncle on FA skeleton | 529 |
| Mean ICVF in pontine crossing tract on FA skeleton | 529 |
| Mean ICVF in genu of corpus callosum on FA skeleton | 529 |
| Mean ICVF in body of corpus callosum on FA skeleton | 529 |
| Mean ICVF in splenium of corpus callosum on FA skeleton | 529 |
| Mean ICVF in fornix on FA skeleton | 529 |
| Mean ICVF in corticospinal tract on FA skeleton (left) | 529 |
| Mean ICVF in corticospinal tract on FA skeleton (right) | 529 |
| Mean ICVF in medial lemniscus on FA skeleton (left) | 529 |
| Mean ICVF in medial lemniscus on FA skeleton (right) | 529 |
| Mean ICVF in inferior cerebellar peduncle on FA skeleton (left) | 529 |
| Mean ICVF in inferior cerebellar peduncle on FA skeleton (right) | 529 |
| Mean ICVF in superior cerebellar peduncle on FA skeleton (left) | 529 |
| Mean ICVF in superior cerebellar peduncle on FA skeleton (right) | 529 |
| Mean ICVF in cerebral peduncle on FA skeleton (left) | 529 |
| Mean ICVF in cerebral peduncle on FA skeleton (right) | 529 |
| Mean ICVF in anterior limb of internal capsule on FA skeleton (left) | 529 |
| Mean ICVF in anterior limb of internal capsule on FA skeleton (right) | 529 |
| Mean ICVF in posterior limb of internal capsule on FA skeleton (left) | 529 |
| Mean ICVF in posterior limb of internal capsule on FA skeleton (right) | 529 |
| Mean ICVF in retrolenticular part of internal capsule on FA skeleton (left) | 529 |
| Mean ICVF in retrolenticular part of internal capsule on FA skeleton (right) | 529 |
| Mean ICVF in anterior corona radiata on FA skeleton (left) | 529 |
| Mean ICVF in anterior corona radiata on FA skeleton (right) | 529 |
| Mean ICVF in superior corona radiata on FA skeleton (left) | 529 |
| Mean ICVF in superior corona radiata on FA skeleton (right) | 529 |
| Mean ICVF in posterior corona radiata on FA skeleton (left) | 529 |
| Mean ICVF in posterior corona radiata on FA skeleton (right) | 529 |
| Mean ICVF in posterior thalamic radiation on FA skeleton (left) | 529 |
| Mean ICVF in posterior thalamic radiation on FA skeleton (right) | 529 |
| Mean ICVF in sagittal stratum on FA skeleton (left) | 529 |
| Mean ICVF in sagittal stratum on FA skeleton (right) | 529 |
| Mean ICVF in external capsule on FA skeleton (left) | 529 |
| Mean ICVF in external capsule on FA skeleton (right) | 529 |
| Mean ICVF in cingulum cingulate gyrus on FA skeleton (left) | 529 |
| Mean ICVF in cingulum cingulate gyrus on FA skeleton (right) | 529 |
| Mean ICVF in cingulum hippocampus on FA skeleton (left) | 529 |
| Mean ICVF in cingulum hippocampus on FA skeleton (right) | 529 |
| Mean ICVF in fornix cres+stria terminalis on FA skeleton (left) | 529 |
| Mean ICVF in fornix cres+stria terminalis on FA skeleton (right) | 529 |
| Mean ICVF in superior longitudinal fasciculus on FA skeleton (left) | 529 |
| Mean ICVF in superior longitudinal fasciculus on FA skeleton (right) | 529 |
| Mean ICVF in superior fronto-occipital fasciculus on FA skeleton (left) | 529 |
| Mean ICVF in superior fronto-occipital fasciculus on FA skeleton (right) | 529 |
| Mean ICVF in uncinate fasciculus on FA skeleton (left) | 529 |
| Mean ICVF in uncinate fasciculus on FA skeleton (right) | 529 |
| Mean ICVF in tapetum on FA skeleton (left) | 529 |
| Mean ICVF in tapetum on FA skeleton (right) | 529 |
| Mean OD in middle cerebellar peduncle on FA skeleton | 529 |
| Mean OD in pontine crossing tract on FA skeleton | 529 |
| Mean OD in genu of corpus callosum on FA skeleton | 529 |
| Mean OD in body of corpus callosum on FA skeleton | 529 |
| Mean OD in splenium of corpus callosum on FA skeleton | 529 |
| Mean OD in fornix on FA skeleton | 529 |
| Mean OD in corticospinal tract on FA skeleton (left) | 529 |
| Mean OD in corticospinal tract on FA skeleton (right) | 529 |
| Mean OD in medial lemniscus on FA skeleton (left) | 529 |
| Mean OD in medial lemniscus on FA skeleton (right) | 529 |
| Mean OD in inferior cerebellar peduncle on FA skeleton (left) | 529 |
| Mean OD in inferior cerebellar peduncle on FA skeleton (right) | 529 |
| Mean OD in superior cerebellar peduncle on FA skeleton (left) | 529 |
| Mean OD in superior cerebellar peduncle on FA skeleton (right) | 529 |
| Mean OD in cerebral peduncle on FA skeleton (left) | 529 |
| Mean OD in cerebral peduncle on FA skeleton (right) | 529 |
| Mean OD in anterior limb of internal capsule on FA skeleton (left) | 529 |
| Mean OD in anterior limb of internal capsule on FA skeleton (right) | 529 |
| Mean OD in posterior limb of internal capsule on FA skeleton (left) | 529 |
| Mean OD in posterior limb of internal capsule on FA skeleton (right) | 529 |
| Mean OD in retrolenticular part of internal capsule on FA skeleton (left) | 529 |
| Mean OD in retrolenticular part of internal capsule on FA skeleton (right) | 529 |
| Mean OD in anterior corona radiata on FA skeleton (left) | 529 |
| Mean OD in anterior corona radiata on FA skeleton (right) | 529 |
| Mean OD in superior corona radiata on FA skeleton (left) | 529 |
| Mean OD in superior corona radiata on FA skeleton (right) | 529 |
| Mean OD in posterior corona radiata on FA skeleton (left) | 529 |
| Mean OD in posterior corona radiata on FA skeleton (right) | 529 |
| Mean OD in posterior thalamic radiation on FA skeleton (left) | 529 |
| Mean OD in posterior thalamic radiation on FA skeleton (right) | 529 |
| Mean OD in sagittal stratum on FA skeleton (left) | 529 |
| Mean OD in sagittal stratum on FA skeleton (right) | 529 |
| Mean OD in external capsule on FA skeleton (left) | 529 |
| Mean OD in external capsule on FA skeleton (right) | 529 |
| Mean OD in cingulum cingulate gyrus on FA skeleton (left) | 529 |
| Mean OD in cingulum cingulate gyrus on FA skeleton (right) | 529 |
| Mean OD in cingulum hippocampus on FA skeleton (left) | 529 |
| Mean OD in cingulum hippocampus on FA skeleton (right) | 529 |
| Mean OD in fornix cres+stria terminalis on FA skeleton (left) | 529 |
| Mean OD in fornix cres+stria terminalis on FA skeleton (right) | 529 |
| Mean OD in superior longitudinal fasciculus on FA skeleton (left) | 529 |
| Mean OD in superior longitudinal fasciculus on FA skeleton (right) | 529 |
| Mean OD in superior fronto-occipital fasciculus on FA skeleton (left) | 529 |
| Mean OD in superior fronto-occipital fasciculus on FA skeleton (right) | 529 |
| Mean OD in uncinate fasciculus on FA skeleton (left) | 529 |
| Mean OD in uncinate fasciculus on FA skeleton (right) | 529 |
| Mean OD in tapetum on FA skeleton (left) | 529 |
| Mean OD in tapetum on FA skeleton (right) | 529 |
| Mean ISOVF in middle cerebellar peduncle on FA skeleton | 529 |
| Mean ISOVF in pontine crossing tract on FA skeleton | 529 |
| Mean ISOVF in genu of corpus callosum on FA skeleton | 529 |
| Mean ISOVF in body of corpus callosum on FA skeleton | 529 |
| Mean ISOVF in splenium of corpus callosum on FA skeleton | 529 |
| Mean ISOVF in fornix on FA skeleton | 529 |
| Mean ISOVF in corticospinal tract on FA skeleton (left) | 529 |
| Mean ISOVF in corticospinal tract on FA skeleton (right) | 529 |
| Mean ISOVF in medial lemniscus on FA skeleton (left) | 529 |
| Mean ISOVF in medial lemniscus on FA skeleton (right) | 529 |
| Mean ISOVF in inferior cerebellar peduncle on FA skeleton (left) | 529 |
| Mean ISOVF in inferior cerebellar peduncle on FA skeleton (right) | 529 |
| Mean ISOVF in superior cerebellar peduncle on FA skeleton (left) | 529 |
| Mean ISOVF in superior cerebellar peduncle on FA skeleton (right) | 529 |
| Mean ISOVF in cerebral peduncle on FA skeleton (left) | 529 |
| Mean ISOVF in cerebral peduncle on FA skeleton (right) | 529 |
| Mean ISOVF in anterior limb of internal capsule on FA skeleton (left) | 529 |
| Mean ISOVF in anterior limb of internal capsule on FA skeleton (right) | 529 |
| Mean ISOVF in posterior limb of internal capsule on FA skeleton (left) | 529 |
| Mean ISOVF in posterior limb of internal capsule on FA skeleton (right) | 529 |
| Mean ISOVF in retrolenticular part of internal capsule on FA skeleton (left) | 529 |
| Mean ISOVF in retrolenticular part of internal capsule on FA skeleton (right) | 529 |
| Mean ISOVF in anterior corona radiata on FA skeleton (left) | 529 |
| Mean ISOVF in anterior corona radiata on FA skeleton (right) | 529 |
| Mean ISOVF in superior corona radiata on FA skeleton (left) | 529 |
| Mean ISOVF in superior corona radiata on FA skeleton (right) | 529 |
| Mean ISOVF in posterior corona radiata on FA skeleton (left) | 529 |
| Mean ISOVF in posterior corona radiata on FA skeleton (right) | 529 |
| Mean ISOVF in posterior thalamic radiation on FA skeleton (left) | 529 |
| Mean ISOVF in posterior thalamic radiation on FA skeleton (right) | 529 |
| Mean ISOVF in sagittal stratum on FA skeleton (left) | 529 |
| Mean ISOVF in sagittal stratum on FA skeleton (right) | 529 |
| Mean ISOVF in external capsule on FA skeleton (left) | 529 |
| Mean ISOVF in external capsule on FA skeleton (right) | 529 |
| Mean ISOVF in cingulum cingulate gyrus on FA skeleton (left) | 529 |
| Mean ISOVF in cingulum cingulate gyrus on FA skeleton (right) | 529 |
| Mean ISOVF in cingulum hippocampus on FA skeleton (left) | 529 |
| Mean ISOVF in cingulum hippocampus on FA skeleton (right) | 529 |
| Mean ISOVF in fornix cres+stria terminalis on FA skeleton (left) | 529 |
| Mean ISOVF in fornix cres+stria terminalis on FA skeleton (right) | 529 |
| Mean ISOVF in superior longitudinal fasciculus on FA skeleton (left) | 529 |
| Mean ISOVF in superior longitudinal fasciculus on FA skeleton (right) | 529 |
| Mean ISOVF in superior fronto-occipital fasciculus on FA skeleton (left) | 529 |
| Mean ISOVF in superior fronto-occipital fasciculus on FA skeleton (right) | 529 |
| Mean ISOVF in uncinate fasciculus on FA skeleton (left) | 529 |
| Mean ISOVF in uncinate fasciculus on FA skeleton (right) | 529 |
| Mean ISOVF in tapetum on FA skeleton (left) | 529 |
| Mean ISOVF in tapetum on FA skeleton (right) | 529 |
| Weighted-mean FA in tract acoustic radiation (left) | 528 |
| Weighted-mean FA in tract acoustic radiation (right) | 528 |
| Weighted-mean FA in tract anterior thalamic radiation (left) | 528 |
| Weighted-mean FA in tract anterior thalamic radiation (right) | 528 |
| Weighted-mean FA in tract cingulate gyrus part of cingulum (left) | 528 |
| Weighted-mean FA in tract cingulate gyrus part of cingulum (right) | 528 |
| Weighted-mean FA in tract parahippocampal part of cingulum (left) | 528 |
| Weighted-mean FA in tract parahippocampal part of cingulum (right) | 528 |
| Weighted-mean FA in tract corticospinal tract (left) | 528 |
| Weighted-mean FA in tract corticospinal tract (right) | 528 |
| Weighted-mean FA in tract forceps major | 528 |
| Weighted-mean FA in tract forceps minor | 528 |
| Weighted-mean FA in tract inferior fronto-occipital fasciculus (left) | 528 |
| Weighted-mean FA in tract inferior fronto-occipital fasciculus (right) | 528 |
| Weighted-mean FA in tract inferior longitudinal fasciculus (left) | 528 |
| Weighted-mean FA in tract inferior longitudinal fasciculus (right) | 528 |
| Weighted-mean FA in tract middle cerebellar peduncle | 528 |
| Weighted-mean FA in tract medial lemniscus (left) | 528 |
| Weighted-mean FA in tract medial lemniscus (right) | 528 |
| Weighted-mean FA in tract posterior thalamic radiation (left) | 528 |
| Weighted-mean FA in tract posterior thalamic radiation (right) | 528 |
| Weighted-mean FA in tract superior longitudinal fasciculus (left) | 528 |
| Weighted-mean FA in tract superior longitudinal fasciculus (right) | 528 |
| Weighted-mean FA in tract superior thalamic radiation (left) | 528 |
| Weighted-mean FA in tract superior thalamic radiation (right) | 528 |
| Weighted-mean FA in tract uncinate fasciculus (left) | 528 |
| Weighted-mean FA in tract uncinate fasciculus (right) | 528 |
| Weighted-mean MD in tract acoustic radiation (left) | 528 |
| Weighted-mean MD in tract acoustic radiation (right) | 528 |
| Weighted-mean MD in tract anterior thalamic radiation (left) | 528 |
| Weighted-mean MD in tract anterior thalamic radiation (right) | 528 |
| Weighted-mean MD in tract cingulate gyrus part of cingulum (left) | 528 |
| Weighted-mean MD in tract cingulate gyrus part of cingulum (right) | 528 |
| Weighted-mean MD in tract parahippocampal part of cingulum (left) | 528 |
| Weighted-mean MD in tract parahippocampal part of cingulum (right) | 528 |
| Weighted-mean MD in tract corticospinal tract (left) | 528 |
| Weighted-mean MD in tract corticospinal tract (right) | 528 |
| Weighted-mean MD in tract forceps major | 528 |
| Weighted-mean MD in tract forceps minor | 528 |
| Weighted-mean MD in tract inferior fronto-occipital fasciculus (left) | 528 |
| Weighted-mean MD in tract inferior fronto-occipital fasciculus (right) | 528 |
| Weighted-mean MD in tract inferior longitudinal fasciculus (left) | 528 |
| Weighted-mean MD in tract inferior longitudinal fasciculus (right) | 528 |
| Weighted-mean MD in tract middle cerebellar peduncle | 528 |
| Weighted-mean MD in tract medial lemniscus (left) | 528 |
| Weighted-mean MD in tract medial lemniscus (right) | 528 |
| Weighted-mean MD in tract posterior thalamic radiation (left) | 528 |
| Weighted-mean MD in tract posterior thalamic radiation (right) | 528 |
| Weighted-mean MD in tract superior longitudinal fasciculus (left) | 528 |
| Weighted-mean MD in tract superior longitudinal fasciculus (right) | 528 |
| Weighted-mean MD in tract superior thalamic radiation (left) | 528 |
| Weighted-mean MD in tract superior thalamic radiation (right) | 528 |
| Weighted-mean MD in tract uncinate fasciculus (left) | 528 |
| Weighted-mean MD in tract uncinate fasciculus (right) | 528 |
| Weighted-mean MO in tract acoustic radiation (left) | 528 |
| Weighted-mean MO in tract acoustic radiation (right) | 528 |
| Weighted-mean MO in tract anterior thalamic radiation (left) | 528 |
| Weighted-mean MO in tract anterior thalamic radiation (right) | 528 |
| Weighted-mean MO in tract cingulate gyrus part of cingulum (left) | 528 |
| Weighted-mean MO in tract cingulate gyrus part of cingulum (right) | 528 |
| Weighted-mean MO in tract parahippocampal part of cingulum (left) | 528 |
| Weighted-mean MO in tract parahippocampal part of cingulum (right) | 528 |
| Weighted-mean MO in tract corticospinal tract (left) | 528 |
| Weighted-mean MO in tract corticospinal tract (right) | 528 |
| Weighted-mean MO in tract forceps major | 528 |
| Weighted-mean MO in tract forceps minor | 528 |
| Weighted-mean MO in tract inferior fronto-occipital fasciculus (left) | 528 |
| Weighted-mean MO in tract inferior fronto-occipital fasciculus (right) | 528 |
| Weighted-mean MO in tract inferior longitudinal fasciculus (left) | 528 |
| Weighted-mean MO in tract inferior longitudinal fasciculus (right) | 528 |
| Weighted-mean MO in tract middle cerebellar peduncle | 528 |
| Weighted-mean MO in tract medial lemniscus (left) | 528 |
| Weighted-mean MO in tract medial lemniscus (right) | 528 |
| Weighted-mean MO in tract posterior thalamic radiation (left) | 528 |
| Weighted-mean MO in tract posterior thalamic radiation (right) | 528 |
| Weighted-mean MO in tract superior longitudinal fasciculus (left) | 528 |
| Weighted-mean MO in tract superior longitudinal fasciculus (right) | 528 |
| Weighted-mean MO in tract superior thalamic radiation (left) | 528 |
| Weighted-mean MO in tract superior thalamic radiation (right) | 528 |
| Weighted-mean MO in tract uncinate fasciculus (left) | 528 |
| Weighted-mean MO in tract uncinate fasciculus (right) | 528 |
| Weighted-mean L1 in tract acoustic radiation (left) | 528 |
| Weighted-mean L1 in tract acoustic radiation (right) | 528 |
| Weighted-mean L1 in tract anterior thalamic radiation (left) | 528 |
| Weighted-mean L1 in tract anterior thalamic radiation (right) | 528 |
| Weighted-mean L1 in tract cingulate gyrus part of cingulum (left) | 528 |
| Weighted-mean L1 in tract cingulate gyrus part of cingulum (right) | 528 |
| Weighted-mean L1 in tract parahippocampal part of cingulum (left) | 528 |
| Weighted-mean L1 in tract parahippocampal part of cingulum (right) | 528 |
| Weighted-mean L1 in tract corticospinal tract (left) | 528 |
| Weighted-mean L1 in tract corticospinal tract (right) | 528 |
| Weighted-mean L1 in tract forceps major | 528 |
| Weighted-mean L1 in tract forceps minor | 528 |
| Weighted-mean L1 in tract inferior fronto-occipital fasciculus (left) | 528 |
| Weighted-mean L1 in tract inferior fronto-occipital fasciculus (right) | 528 |
| Weighted-mean L1 in tract inferior longitudinal fasciculus (left) | 528 |
| Weighted-mean L1 in tract inferior longitudinal fasciculus (right) | 528 |
| Weighted-mean L1 in tract middle cerebellar peduncle | 528 |
| Weighted-mean L1 in tract medial lemniscus (left) | 528 |
| Weighted-mean L1 in tract medial lemniscus (right) | 528 |
| Weighted-mean L1 in tract posterior thalamic radiation (left) | 528 |
| Weighted-mean L1 in tract posterior thalamic radiation (right) | 528 |
| Weighted-mean L1 in tract superior longitudinal fasciculus (left) | 528 |
| Weighted-mean L1 in tract superior longitudinal fasciculus (right) | 528 |
| Weighted-mean L1 in tract superior thalamic radiation (left) | 528 |
| Weighted-mean L1 in tract superior thalamic radiation (right) | 528 |
| Weighted-mean L1 in tract uncinate fasciculus (left) | 528 |
| Weighted-mean L1 in tract uncinate fasciculus (right) | 528 |
| Weighted-mean L2 in tract acoustic radiation (left) | 528 |
| Weighted-mean L2 in tract acoustic radiation (right) | 528 |
| Weighted-mean L2 in tract anterior thalamic radiation (left) | 528 |
| Weighted-mean L2 in tract anterior thalamic radiation (right) | 528 |
| Weighted-mean L2 in tract cingulate gyrus part of cingulum (left) | 528 |
| Weighted-mean L2 in tract cingulate gyrus part of cingulum (right) | 528 |
| Weighted-mean L2 in tract parahippocampal part of cingulum (left) | 528 |
| Weighted-mean L2 in tract parahippocampal part of cingulum (right) | 528 |
| Weighted-mean L2 in tract corticospinal tract (left) | 528 |
| Weighted-mean L2 in tract corticospinal tract (right) | 528 |
| Weighted-mean L2 in tract forceps major | 528 |
| Weighted-mean L2 in tract forceps minor | 528 |
| Weighted-mean L2 in tract inferior fronto-occipital fasciculus (left) | 528 |
| Weighted-mean L2 in tract inferior fronto-occipital fasciculus (right) | 528 |
| Weighted-mean L2 in tract inferior longitudinal fasciculus (left) | 528 |
| Weighted-mean L2 in tract inferior longitudinal fasciculus (right) | 528 |
| Weighted-mean L2 in tract middle cerebellar peduncle | 528 |
| Weighted-mean L2 in tract medial lemniscus (left) | 528 |
| Weighted-mean L2 in tract medial lemniscus (right) | 528 |
| Weighted-mean L2 in tract posterior thalamic radiation (left) | 528 |
| Weighted-mean L2 in tract posterior thalamic radiation (right) | 528 |
| Weighted-mean L2 in tract superior longitudinal fasciculus (left) | 528 |
| Weighted-mean L2 in tract superior longitudinal fasciculus (right) | 528 |
| Weighted-mean L2 in tract superior thalamic radiation (left) | 528 |
| Weighted-mean L2 in tract superior thalamic radiation (right) | 528 |
| Weighted-mean L2 in tract uncinate fasciculus (left) | 528 |
| Weighted-mean L2 in tract uncinate fasciculus (right) | 528 |
| Weighted-mean L3 in tract acoustic radiation (left) | 528 |
| Weighted-mean L3 in tract acoustic radiation (right) | 528 |
| Weighted-mean L3 in tract anterior thalamic radiation (left) | 528 |
| Weighted-mean L3 in tract anterior thalamic radiation (right) | 528 |
| Weighted-mean L3 in tract cingulate gyrus part of cingulum (left) | 528 |
| Weighted-mean L3 in tract cingulate gyrus part of cingulum (right) | 528 |
| Weighted-mean L3 in tract parahippocampal part of cingulum (left) | 528 |
| Weighted-mean L3 in tract parahippocampal part of cingulum (right) | 528 |
| Weighted-mean L3 in tract corticospinal tract (left) | 528 |
| Weighted-mean L3 in tract corticospinal tract (right) | 528 |
| Weighted-mean L3 in tract forceps major | 528 |
| Weighted-mean L3 in tract forceps minor | 528 |
| Weighted-mean L3 in tract inferior fronto-occipital fasciculus (left) | 528 |
| Weighted-mean L3 in tract inferior fronto-occipital fasciculus (right) | 528 |
| Weighted-mean L3 in tract inferior longitudinal fasciculus (left) | 528 |
| Weighted-mean L3 in tract inferior longitudinal fasciculus (right) | 528 |
| Weighted-mean L3 in tract middle cerebellar peduncle | 528 |
| Weighted-mean L3 in tract medial lemniscus (left) | 528 |
| Weighted-mean L3 in tract medial lemniscus (right) | 528 |
| Weighted-mean L3 in tract posterior thalamic radiation (left) | 528 |
| Weighted-mean L3 in tract posterior thalamic radiation (right) | 528 |
| Weighted-mean L3 in tract superior longitudinal fasciculus (left) | 528 |
| Weighted-mean L3 in tract superior longitudinal fasciculus (right) | 528 |
| Weighted-mean L3 in tract superior thalamic radiation (left) | 528 |
| Weighted-mean L3 in tract superior thalamic radiation (right) | 528 |
| Weighted-mean L3 in tract uncinate fasciculus (left) | 528 |
| Weighted-mean L3 in tract uncinate fasciculus (right) | 528 |
| Weighted-mean ICVF in tract acoustic radiation (left) | 529 |
| Weighted-mean ICVF in tract acoustic radiation (right) | 529 |
| Weighted-mean ICVF in tract anterior thalamic radiation (left) | 529 |
| Weighted-mean ICVF in tract anterior thalamic radiation (right) | 529 |
| Weighted-mean ICVF in tract cingulate gyrus part of cingulum (left) | 529 |
| Weighted-mean ICVF in tract cingulate gyrus part of cingulum (right) | 529 |
| Weighted-mean ICVF in tract parahippocampal part of cingulum (left) | 529 |
| Weighted-mean ICVF in tract parahippocampal part of cingulum (right) | 529 |
| Weighted-mean ICVF in tract corticospinal tract (left) | 529 |
| Weighted-mean ICVF in tract corticospinal tract (right) | 529 |
| Weighted-mean ICVF in tract forceps major | 529 |
| Weighted-mean ICVF in tract forceps minor | 529 |
| Weighted-mean ICVF in tract inferior fronto-occipital fasciculus (left) | 529 |
| Weighted-mean ICVF in tract inferior fronto-occipital fasciculus (right) | 529 |
| Weighted-mean ICVF in tract inferior longitudinal fasciculus (left) | 529 |
| Weighted-mean ICVF in tract inferior longitudinal fasciculus (right) | 529 |
| Weighted-mean ICVF in tract middle cerebellar peduncle | 529 |
| Weighted-mean ICVF in tract medial lemniscus (left) | 529 |
| Weighted-mean ICVF in tract medial lemniscus (right) | 529 |
| Weighted-mean ICVF in tract posterior thalamic radiation (left) | 529 |
| Weighted-mean ICVF in tract posterior thalamic radiation (right) | 529 |
| Weighted-mean ICVF in tract superior longitudinal fasciculus (left) | 529 |
| Weighted-mean ICVF in tract superior longitudinal fasciculus (right) | 529 |
| Weighted-mean ICVF in tract superior thalamic radiation (left) | 529 |
| Weighted-mean ICVF in tract superior thalamic radiation (right) | 529 |
| Weighted-mean ICVF in tract uncinate fasciculus (left) | 529 |
| Weighted-mean ICVF in tract uncinate fasciculus (right) | 529 |
| Weighted-mean OD in tract acoustic radiation (left) | 529 |
| Weighted-mean OD in tract acoustic radiation (right) | 529 |
| Weighted-mean OD in tract anterior thalamic radiation (left) | 529 |
| Weighted-mean OD in tract anterior thalamic radiation (right) | 529 |
| Weighted-mean OD in tract cingulate gyrus part of cingulum (left) | 529 |
| Weighted-mean OD in tract cingulate gyrus part of cingulum (right) | 529 |
| Weighted-mean OD in tract parahippocampal part of cingulum (left) | 529 |
| Weighted-mean OD in tract parahippocampal part of cingulum (right) | 529 |
| Weighted-mean OD in tract corticospinal tract (left) | 529 |
| Weighted-mean OD in tract corticospinal tract (right) | 529 |
| Weighted-mean OD in tract forceps major | 529 |
| Weighted-mean OD in tract forceps minor | 529 |
| Weighted-mean OD in tract inferior fronto-occipital fasciculus (left) | 529 |
| Weighted-mean OD in tract inferior fronto-occipital fasciculus (right) | 529 |
| Weighted-mean OD in tract inferior longitudinal fasciculus (left) | 529 |
| Weighted-mean OD in tract inferior longitudinal fasciculus (right) | 529 |
| Weighted-mean OD in tract middle cerebellar peduncle | 529 |
| Weighted-mean OD in tract medial lemniscus (left) | 529 |
| Weighted-mean OD in tract medial lemniscus (right) | 529 |
| Weighted-mean OD in tract posterior thalamic radiation (left) | 529 |
| Weighted-mean OD in tract posterior thalamic radiation (right) | 529 |
| Weighted-mean OD in tract superior longitudinal fasciculus (left) | 529 |
| Weighted-mean OD in tract superior longitudinal fasciculus (right) | 529 |
| Weighted-mean OD in tract superior thalamic radiation (left) | 529 |
| Weighted-mean OD in tract superior thalamic radiation (right) | 529 |
| Weighted-mean OD in tract uncinate fasciculus (left) | 529 |
| Weighted-mean OD in tract uncinate fasciculus (right) | 529 |
| Weighted-mean ISOVF in tract acoustic radiation (left) | 529 |
| Weighted-mean ISOVF in tract acoustic radiation (right) | 529 |
| Weighted-mean ISOVF in tract anterior thalamic radiation (left) | 529 |
| Weighted-mean ISOVF in tract anterior thalamic radiation (right) | 529 |
| Weighted-mean ISOVF in tract cingulate gyrus part of cingulum (left) | 529 |
| Weighted-mean ISOVF in tract cingulate gyrus part of cingulum (right) | 529 |
| Weighted-mean ISOVF in tract parahippocampal part of cingulum (left) | 529 |
| Weighted-mean ISOVF in tract parahippocampal part of cingulum (right) | 529 |
| Weighted-mean ISOVF in tract corticospinal tract (left) | 529 |
| Weighted-mean ISOVF in tract corticospinal tract (right) | 529 |
| Weighted-mean ISOVF in tract forceps major | 529 |
| Weighted-mean ISOVF in tract forceps minor | 529 |
| Weighted-mean ISOVF in tract inferior fronto-occipital fasciculus (left) | 529 |
| Weighted-mean ISOVF in tract inferior fronto-occipital fasciculus (right) | 529 |
| Weighted-mean ISOVF in tract inferior longitudinal fasciculus (left) | 529 |
| Weighted-mean ISOVF in tract inferior longitudinal fasciculus (right) | 529 |
| Weighted-mean ISOVF in tract middle cerebellar peduncle | 529 |
| Weighted-mean ISOVF in tract medial lemniscus (left) | 529 |
| Weighted-mean ISOVF in tract medial lemniscus (right) | 529 |
| Weighted-mean ISOVF in tract posterior thalamic radiation (left) | 529 |
| Weighted-mean ISOVF in tract posterior thalamic radiation (right) | 529 |
| Weighted-mean ISOVF in tract superior longitudinal fasciculus (left) | 529 |
| Weighted-mean ISOVF in tract superior longitudinal fasciculus (right) | 529 |
| Weighted-mean ISOVF in tract superior thalamic radiation (left) | 529 |
| Weighted-mean ISOVF in tract superior thalamic radiation (right) | 529 |
| Weighted-mean ISOVF in tract uncinate fasciculus (left) | 529 |
| Weighted-mean ISOVF in tract uncinate fasciculus (right) | 529 |
| Median BOLD effect (in group-defined mask) for shapes activation | 5901 |
| Median z-statistic (in group-defined mask) for shapes activation | 5901 |
| Median BOLD effect (in group-defined mask) for faces activation | 5901 |
| Median z-statistic (in group-defined mask) for faces activation | 5901 |
| Median BOLD effect (in group-defined mask) for faces-shapes contrast | 5901 |
| Median BOLD effect (in group-defined amygdala activation mask) for faces-shapes contrast | 5901 |
| Median z-statistic (in group-defined amygdala activation mask) for faces-shapes contrast | 5901 |
| 90th percentile of BOLD effect (in group-defined mask) for shapes activation | 5901 |
| 90th percentile of z-statistic (in group-defined mask) for shapes activation | 5901 |
| 90th percentile of BOLD effect (in group-defined mask) for faces activation | 5901 |
| 90th percentile of z-statistic (in group-defined mask) for faces activation | 5901 |
| 90th percentile of BOLD effect (in group-defined mask) for faces-shapes contrast | 5901 |
| 90th percentile of BOLD effect (in group-defined amygdala activation mask) for faces-shapes contrast | 5901 |
| 90th percentile of z-statistic (in group-defined amygdala activation mask) for faces-shapes contrast | 5901 |
| rfMRI partial correlation matrix, dimension 25* | 0 |
| Abbreviations: IDPs, imaging-derived phenotypes |  |
| *rfMRI partial correlation matrix is a partial correlation matrix with 25 dimensionalities, that had been converted into vectors with 210 elements | |

| **sTable2 Neurological disorders used as exclusion criteria for the neuroimaging subsample** | |
| --- | --- |
| **Self-reported illness** | **Code** |
| **Field ID 20001** |  |
| Meningeal cancer | 1031 |
| Brain cancer | 1032 |
| **Field ID 20002** |  |
| Stroke or ischemic stroke | 1081 |
| Transient ischemic attack | 1082 |
| Subdural hematoma | 1083 |
| Subarachnoid hemorrhage | 1086 |
| Neurological injury/trauma | 1240 |
| Infections of the nervous system | 1244 |
| Brain/intracranial abscess | 1245 |
| Encephalitis | 1246 |
| Meningitis | 1247 |
| Guillain-Barré syndrome | 1256 |
| Chronic degenerative neurological | 1258 |
| Motor Neuron Disease | 1259 |
| Multiple Sclerosis | 1261 |
| Parkinson's disease | 1262 |
| Dementia or Alzheimer's disease | 1263 |
| Epilepsy | 1264 |
| Head injury | 1266 |
| Other demyelinating diseases | 1397 |
| Cerebral aneurysm | 1425 |
| Cerebral palsy | 1433 |
| Other neurological problem | 1434 |
| Brain hemorrhage | 1491 |
| Spina bifida | 1524 |
| Ischemic stroke | 1583 |
| Meningioma (benign) | 1659 |
| Neuroma (benign) | 1683 |

| **sTable3 Brain MRI image acquisition protocol and IDPs collected by various modalities** | | | | |
| --- | --- | --- | --- | --- |
| **Modalities** | **Duration (minutes)** | **Voxel, Matrix** | **Key Parameters** | **IDPs needed** |
| T1 | 4,54 | 1×1×1 mm, 208×256×256 | 3D MPRAGE, sagittal, R=2, TI/TR=880/2000 ms | 165 |
| T2 FLAIR | 5,52 | 1.05×1.0×1.0 mm 192×256×256 | FLAIR, 3D SPACE, sagittal, R=2, PF 7/8, fat sat, TI/TR=1800/5000 ms, elliptical | 1 |
| T2 * | 2,34 | 0.8×0.8×3 mm 256×288×48 | 3D GRE, axial, R=2, PF 7/8 TE1/TE2/TR = 9.4/20/27 ms | 14 |
| Diffusion MRI | 7,08 | 2.0×2.0×2.0 mm 104×104×72 | MB=3, R=1, TE/TR=92/3600 ms, PF 6/8, fat sat, b=0 s/mm2 (5x + 3×phase-encoding reversed), b=1 000 s/mm2 (50×), b=2000 s/mm2 (50×) | 675 |
| Resting-state fMRI | 6,10 | 2.4×2.4×2.4 mm 88×88×64 | TE/TR = 39/735 ms, MB = 8, R = 1, flip angle 52°, fat sat | 210 |
| Task fMRI | 4,13 | 2.4×2.4×2.4 mm 88×88×64 | Acquisition same as rfMRI. Task is faces/shapes “emotion” task. | 14 |
| Abbreviations: R, in-plane acceleration factor; MB, multiband factor; PF, partial Fourier; IDPs, imaging-derived phenotypes. | | | |  |

| **sTable4 Hyperparameter ranges for each model** |  |  |  |
| --- | --- | --- | --- |
| **Model** | **Feature Selector** | **Hyperparameter** | **Range** |
| **XGBoost** | no feature selector | alpha | 0.001 to 1000 |
| **XGBoost** | no feature selector | gamma | 0.001 to 1000 |
| **XGBoost** | no feature selector | learning_rate | 0.01 to 0.3 |
| **XGBoost** | no feature selector | max_depth | 2 to 6 |
| **XGBoost** | no feature selector | n_estimators | 200 to 800 |
| **XGBoost** | no feature selector | reg_lambda | 0.001 to 1000 |
| **XGBoost** | no feature selector | subsample | 0.6 to 1 |
| **XGBoost** | FeatureWiz | alpha | 0.001 to 1000 |
| **XGBoost** | FeatureWiz | gamma | 0.001 to 1000 |
| **XGBoost** | FeatureWiz | learning_rate | 0.01 to 0.3 |
| **XGBoost** | FeatureWiz | max_depth | 2 to 6 |
| **XGBoost** | FeatureWiz | n_estimators | 200 to 800 |
| **XGBoost** | FeatureWiz | reg_lambda | 0.001 to 1000 |
| **XGBoost** | FeatureWiz | subsample | 0.6 to 1 |
| **XGBoost** | RFECV | alpha | 0.001 to 1000 |
| **XGBoost** | RFECV | gamma | 0.001 to 1000 |
| **XGBoost** | RFECV | learning_rate | 0.01 to 0.3 |
| **XGBoost** | RFECV | max_depth | 2 to 6 |
| **XGBoost** | RFECV | n_estimators | 200 to 800 |
| **XGBoost** | RFECV | reg_lambda | 0.001 to 1000 |
| **XGBoost** | RFECV | subsample | 0.6 to 1 |
| **LASSO** | no feature selector | alpha | 0.07 to 1 |
| **LASSO** | FeatureWiz | alpha | 0.07 to 1 |
| **LASSO** | RFECV | alpha | 0.07 to 1 |
| **SVR** | no feature selector | C | 1 to 1000 |
| **SVR** | FeatureWiz | C | 1 to 1000 |
| **SVR** | RFECV | C | 1 to 1000 |
| Abbreviation: XGBoost, eXtreme gradient boosting; LASSO, least absolute shrinkage and selection operator regression; SVR, support vector regression; RFECV, recursive feature elimination with cross validation | | | |

| **sTable5 Best hyperparameter for each model** |  |
| --- | --- |
| **Model/Hyperparameter** | **Value** |
| **None feature Selector** | |
| **XGBoost** |  |
| alpha | 68.531656 |
| gamma | 81.2240555 |
| learning_rate | 0.05888361 |
| max_depth | 2 |
| n_estimators | 523 |
| reg_lambda | 0.05888361 |
| subsample | 0.6 |
| **LASSO** |  |
| alpha | 0.07 |
| **SVR** |  |
| C | 33 |
| **FeatureWiz** | |
| **XGBoost** |  |
| alpha | 4.35917561 |
| gamma | 10.4665242 |
| learning_rate | 0.10992448 |
| max_depth | 5 |
| n_estimators | 787 |
| reg_lambda | 840.543668 |
| subsample | 0.8430267 |
| **LASSO** |  |
| alpha | 0.07 |
| **SVR** |  |
| C | 588 |
| **RFECV** | |
| **XGBoost** |  |
| alpha | 134.27321 |
| gamma | 0.001 |
| learning_rate | 0.3 |
| max_depth | 2 |
| n_estimators | 568 |
| reg_lambda | 637.212531 |
| subsample | 0.6 |
| **LASSO** |  |
| alpha | 0.07 |
| **SVR** |  |
| C | 7 |
| Abbreviation: XGBoost, eXtreme gradient boosting; LASSO, least absolute shrinkage and selection operator regression; SVR, support vector regression; RFECV, recursive feature elimination with cross-validation. | |

| **sTable6 Bayesian optimization process of nine models** | | | | |  |  |  |  |  |  |  |  |  |  |  |  |
| --- | --- | --- | --- | --- | --- | --- | --- | --- | --- | --- | --- | --- | --- | --- | --- | --- |
| **Model, XGBoost. Feature selector, None.** | | | | | | | | |  | **Model, LASSO. Feature selector, None.** | | |  | **Model, SVR. Feature selector, None.** | | |
| iter | target | alpha | gamma | learning_rate | max_depth | n_estimators | reg_lambda | subsample |  | iter | target | alpha |  | iter | target | C |
| 1 | -27.89 | 588 | 699.1 | 0.06456 | 2.175 | 323 | 106.1 | 0.8909 |  | 1 | -24.12 | 0.6169 |  | 1 | -17.43 | 588.4 |
| 2 | -28.24 | 679.4 | 473.8 | 0.14 | 2.076 | 651.6 | 602.4 | 0.9847 |  | 2 | -25.04 | 0.7202 |  | 2 | -17.43 | 699.4 |
| 3 | -30.39 | 664.4 | 606.6 | 0.1403 | 2.901 | 602.1 | 735.8 | 0.7032 |  | 3 | -20.14 | 0.245 |  | 3 | -17.43 | 189 |
| 4 | -26.78 | 95.54 | 960.9 | 0.08301 | 3.129 | 661 | 797.9 | 0.8176 |  | 4 | -17.55 | 0.1107 |  | 4 | -17.43 | 44.76 |
| 5 | -25.9 | 382.7 | 381.7 | 0.09289 | 4.961 | 343.4 | 437.7 | 0.9534 |  | 5 | -20.39 | 0.2607 |  | 5 | -17.43 | 205.8 |
| 6 | -27.67 | 289.3 | 784.5 | 0.2301 | 3.671 | 335.5 | 420.1 | 0.6257 |  | 6 | -18.76 | 0.1686 |  | 6 | -17.43 | 107 |
| 7 | -31.23 | 596.4 | 837.3 | 0.2688 | 2.802 | 501.4 | 895.4 | 0.7024 |  | 7 | -25.27 | 0.7463 |  | 7 | -17.43 | 727.5 |
| 8 | -26.71 | 867.2 | 16.49 | 0.1702 | 4.112 | 754 | 245.9 | 0.6256 |  | 8 | -24.88 | 0.7018 |  | 8 | -17.43 | 679.7 |
| 9 | -31.17 | 902.1 | 874 | 0.05746 | 5.999 | 408.1 | 312.9 | 0.9388 |  | 9 | -23.16 | 0.5107 |  | 9 | -17.43 | 474.4 |
| 10 | -33.41 | 880.2 | 676.6 | 0.02557 | 4.237 | 616.7 | 824.2 | 0.7246 |  | 10 | -22.95 | 0.4869 |  | 10 | -17.43 | 448.8 |
| 11 | -23.06 | 134.5 | 211.2 | 0.3 | 2 | 800 | 312.8 | 1 |  | 11 | -16.76 | 0.07 |  | 11 | -17.43 | 1.00E+03 |
| 12 | -22.86 | 134.8 | 211.5 | 0.01213 | 2.587 | 799.7 | 313.1 | 1 |  | 12 | -16.76 | 0.07 |  | 12 | -17.43 | 36.59 |
| 13 | -23.29 | 143.5 | 220.3 | 0.3 | 3.599 | 791.1 | 321.8 | 1 |  | 13 | -16.76 | 0.07 |  | 13 | -17.43 | 36.59 |
| 14 | -23.24 | 132.1 | 208.7 | 0.01 | 6 | 778 | 310.5 | 0.8539 |  | 14 | -16.76 | 0.07 |  | 14 | -17.43 | 833.8 |
| 15 | -23.15 | 118.7 | 231.2 | 0.01 | 6 | 792.4 | 315 | 1 |  | 15 | -16.76 | 0.07 |  | 15 | -17.43 | 103.8 |
| 16 | -23.3 | 143.4 | 230.5 | 0.01 | 6 | 793.5 | 291.7 | 1 |  | 16 | -16.76 | 0.07 |  | 16 | -17.43 | 36.59 |
| 17 | -23.83 | 117.7 | 204.3 | 0.01 | 6 | 796.3 | 345.7 | 0.6 |  | 17 | -16.76 | 0.07 |  | 17 | -17.43 | 553.8 |
| 18 | -23.79 | 114.9 | 245.2 | 0.01 | 6 | 761.2 | 278.6 | 0.6 |  | 18 | -16.76 | 0.07 |  | 18 | -17.43 | 637 |
| 19 | -23.9 | 119.9 | 274.3 | 0.01 | 6 | 798.5 | 283.8 | 0.6 |  | 19 | -16.76 | 0.07 |  | 19 | -17.43 | 36.58 |
| 20 | -22.94 | 98.93 | 237.8 | 0.01 | 6 | 800 | 258.7 | 1 |  | 20 | -16.76 | 0.07 |  | 20 | -17.43 | 36.58 |
| 21 | -23.39 | 69.28 | 255.2 | 0.01 | 6 | 788.3 | 285 | 0.6 |  | 21 | -16.76 | 0.07 |  | 21 | -17.43 | 267.8 |
| 22 | -22.97 | 84.83 | 273.4 | 0.01 | 6 | 778.7 | 240.3 | 1 |  | 22 | -16.76 | 0.07 |  | 22 | -17.43 | 232.3 |
| 23 | -23.04 | 65.59 | 229.2 | 0.01 | 6 | 764.9 | 241.9 | 0.6 |  | 23 | -16.76 | 0.07 |  | 23 | -17.43 | 36.58 |
| 24 | -22.49 | 42.49 | 254.4 | 0.01 | 6 | 800 | 233.8 | 1 |  | 24 | -16.76 | 0.07 |  | 24 | -17.43 | 880.8 |
| 25 | -22.85 | 78.88 | 238.7 | 0.01 | 6 | 800 | 200.6 | 0.6 |  | 25 | -16.76 | 0.07 |  | 25 | -17.43 | 743.8 |
| 26 | -22.73 | 43.13 | 265 | 0.01 | 6 | 762.9 | 195.4 | 0.6 |  | 26 | -16.76 | 0.07 |  | 26 | -17.43 | 539.6 |
| 27 | -23.01 | 57.6 | 300.5 | 0.01 | 2 | 800 | 195.6 | 0.6 |  | 27 | -16.76 | 0.07 |  | 27 | -17.43 | 36.58 |
| 28 | -23.01 | 100.3 | 276.9 | 0.01 | 6 | 759.5 | 175.8 | 1 |  | 28 | -16.76 | 0.07 |  | 28 | -17.43 | 307.1 |
| 29 | -23.01 | 21.76 | 299.8 | 0.01 | 6 | 764.2 | 243.8 | 0.6 |  | 29 | -16.76 | 0.07 |  | 29 | -17.43 | 935.4 |
| 30 | -23.39 | 64.83 | 319 | 0.01 | 6 | 733.8 | 205.2 | 0.6 |  | 30 | -16.76 | 0.07 |  | 30 | -17.43 | 36.6 |
| 31 | -22.81 | 81.98 | 254 | 0.01 | 6 | 707.8 | 208.3 | 1 |  | 31 | -16.76 | 0.07 |  | 31 | -17.43 | 380.1 |
| 32 | -22.58 | 28.41 | 263.2 | 0.01 | 6 | 708.4 | 243.5 | 1 |  | 32 | -16.76 | 0.07 |  | 32 | -17.43 | 629.5 |
| 33 | -23.9 | 74.77 | 294.3 | 0.01 | 6 | 702.8 | 268 | 0.6 |  | 33 | -16.76 | 0.07 |  | 33 | -17.43 | 36.61 |
| 34 | -22.18 | 23.9 | 217.2 | 0.01 | 6 | 710.8 | 197.2 | 1 |  | 34 | -16.76 | 0.07 |  | 34 | -17.43 | 36.6 |
| 35 | -22.86 | 0.001 | 224.5 | 0.01 | 2 | 752.2 | 232.5 | 1 |  | 35 | -16.76 | 0.07 |  | 35 | -17.43 | 36.57 |
| 36 | -23.04 | 41.94 | 199.1 | 0.01 | 6 | 687.5 | 247.6 | 0.6 |  | 36 | -16.76 | 0.07 |  | 36 | -17.43 | 599 |
| 37 | -22.32 | 74.14 | 195.9 | 0.01 | 6 | 735.5 | 171.9 | 1 |  | 37 | -16.76 | 0.07 |  | 37 | -17.43 | 178.8 |
| 38 | -22.87 | 52.76 | 242.9 | 0.01 | 2 | 707.8 | 139.9 | 0.6 |  | 38 | -16.76 | 0.07 |  | 38 | -17.43 | 478.4 |
| 39 | -23.33 | 68.11 | 193.6 | 0.01 | 2 | 667.9 | 175.6 | 0.7021 |  | 39 | -16.76 | 0.07 |  | 39 | -17.43 | 467.6 |
| 40 | -21.82 | 15.81 | 199.9 | 0.01 | 6 | 766 | 156.6 | 1 |  | 40 | -16.76 | 0.07 |  | 40 | -17.43 | 36.62 |
| 41 | -21.93 | 20 | 153 | 0.01 | 6 | 742 | 190.8 | 0.6 |  | 41 | -16.76 | 0.07 |  | 41 | -17.43 | 36.62 |
| 42 | -21.83 | 34.77 | 170 | 0.01 | 6 | 800 | 196.8 | 1 |  | 42 | -16.76 | 0.07 |  | 42 | -17.43 | 36.55 |
| 43 | -21.66 | 48.41 | 143.9 | 0.01 | 6 | 779.3 | 139.4 | 1 |  | 43 | -16.76 | 0.07 |  | 43 | -17.43 | 36.54 |
| 44 | -21.56 | 21.07 | 158 | 0.01 | 6 | 720.9 | 120.1 | 1 |  | 44 | -16.76 | 0.07 |  | 44 | -17.43 | 36.64 |
| 45 | -20.57 | 0.001 | 106.8 | 0.3 | 2 | 757.9 | 136.3 | 0.6 |  | 45 | -16.76 | 0.07 |  | 45 | -17.43 | 36.52 |
| 46 | -20.37 | 0.001 | 137.2 | 0.01693 | 2 | 779.7 | 89.51 | 0.6 |  | 46 | -16.76 | 0.07 |  | 46 | -17.43 | 36.41 |
| 47 | -21 | 24.65 | 92.29 | 0.01 | 6 | 748.5 | 79.13 | 1 |  | 47 | -16.76 | 0.07 |  | 47 | -17.43 | 36.4 |
| 48 | -20.52 | 0.001 | 76.71 | 0.01 | 6 | 800 | 100.6 | 0.6 |  | 48 | -16.76 | 0.07 |  | 48 | -17.43 | 36.43 |
| 49 | -21.78 | 0.001 | 99.3 | 0.3 | 2 | 800 | 30.91 | 0.6 |  | 49 | -16.76 | 0.07 |  | 49 | -17.43 | 36.5 |
| 50 | -22.44 | 47.62 | 48.87 | 0.01 | 2 | 776.7 | 143.8 | 0.6 |  | 50 | -16.76 | 0.07 |  | 50 | -17.43 | 36.45 |
| 51 | -21.89 | 64.35 | 111.8 | 0.01 | 2 | 800 | 69.41 | 0.6 |  | 51 | -16.76 | 0.07 |  | 51 | -17.43 | 36.48 |
| 52 | -21.15 | 0.001 | 162 | 0.3 | 2 | 732.9 | 33.46 | 0.6 |  | 52 | -16.76 | 0.07 |  | 52 | -17.43 | 36.38 |
| 53 | -21.79 | 19.66 | 203.2 | 0.01 | 6 | 797.4 | 50.91 | 1 |  | 53 | -16.76 | 0.07 |  | 53 | -17.43 | 36.36 |
| 54 | -21.55 | 78.13 | 170 | 0.01 | 6 | 732.2 | 39.85 | 0.6 |  | 54 | -16.76 | 0.07 |  | 54 | -17.43 | 36.66 |
| 55 | -21.19 | 35.69 | 118.1 | 0.3 | 2 | 667.7 | 33.51 | 0.6 |  | 55 | -16.76 | 0.07 |  | 55 | -17.43 | 483.4 |
| 56 | -21.1 | 0.001 | 78.79 | 0.3 | 2 | 669.8 | 103.4 | 0.6 |  | 56 | -16.76 | 0.07 |  | 56 | -17.43 | 36.34 |
| 57 | -22.33 | 0.001 | 44.96 | 0.3 | 2 | 694.6 | 26.68 | 0.6 |  | 57 | -16.76 | 0.07 |  | 57 | -17.43 | 36.75 |
| 58 | -21.22 | 0.001 | 157.9 | 0.01 | 6 | 629.7 | 77.46 | 0.6 |  | 58 | -16.76 | 0.07 |  | 58 | -17.43 | 36.78 |
| 59 | -21.34 | 0.001 | 82.76 | 0.2247 | 6 | 591.4 | 57.53 | 0.6043 |  | 59 | -16.76 | 0.07 |  | 59 | -17.43 | 36.73 |
| 60 | -21.89 | 71.78 | 98.16 | 0.01 | 6 | 620 | 95.12 | 0.6 |  | 60 | -16.76 | 0.07 |  | 60 | -17.43 | 36.8 |
| 61 | -21.66 | 42.52 | 154.4 | 0.01 | 6 | 583.7 | 10.64 | 1 |  | 61 | -16.76 | 0.07 |  | 61 | -17.43 | 36.83 |
| 62 | -23.44 | 0.001 | 104.3 | 0.01 | 2 | 585.6 | 147.8 | 1 |  | 62 | -16.76 | 0.07 |  | 62 | -17.43 | 36.32 |
| 63 | -21.15 | 69.76 | 61.95 | 0.01 | 6 | 599.8 | 0.001 | 1 |  | 63 | -16.76 | 0.07 |  | 63 | -17.43 | 36.85 |
| 64 | -19.29 | 68.53 | 81.22 | 0.05888 | 2 | 523 | 33.37 | 0.6 |  | 64 | -16.76 | 0.07 |  | 64 | -17.43 | 36.94 |
| 65 | -21.75 | 22.34 | 57.51 | 0.3 | 2 | 513.6 | 0.001 | 0.6 |  | 65 | -16.76 | 0.07 |  | 65 | -17.43 | 36.88 |
| 66 | -21.29 | 127.1 | 98.91 | 0.3 | 2 | 535.2 | 18.69 | 0.6 |  | 66 | -16.76 | 0.07 |  | 66 | -17.43 | 36.91 |
| 67 | -22.35 | 60.36 | 129.3 | 0.01 | 6 | 495 | 63.54 | 0.6 |  | 67 | -16.76 | 0.07 |  | 67 | -17.43 | 36.69 |
| 68 | -23.5 | 85.63 | 30.12 | 0.01 | 2 | 538.7 | 59.79 | 0.6 |  | 68 | -16.76 | 0.07 |  | 68 | -17.43 | 36.19 |
| 69 | -23.16 | 64.83 | 109.1 | 0.01 | 2 | 534.5 | 0.001 | 1 |  | 69 | -16.76 | 0.07 |  | 69 | -17.43 | 36.21 |
| 70 | -21.08 | 28.13 | 76.74 | 0.3 | 2 | 523.7 | 63.74 | 0.6 |  | 70 | -16.76 | 0.07 |  | 70 | -17.43 | 36.17 |
| 71 | -20.88 | 85.58 | 67.39 | 0.3 | 2 | 472.4 | 29.39 | 0.6 |  | 71 | -16.76 | 0.07 |  | 71 | -17.43 | 36.15 |
| 72 | -21.77 | 115.1 | 129.4 | 0.01 | 6 | 635.4 | 20.78 | 0.6 |  | 72 | -16.76 | 0.07 |  | 72 | -17.43 | 36.3 |
| 73 | -21.11 | 34.37 | 203.3 | 0.01 | 6 | 658.3 | 17.25 | 0.6228 |  | 73 | -16.76 | 0.07 |  | 73 | -17.43 | 36.13 |
| 74 | -21.92 | 0.001 | 2.504 | 0.01 | 2 | 795.5 | 66.8 | 1 |  | 74 | -16.76 | 0.07 |  | 74 | -17.43 | 36.23 |
| 75 | -22.06 | 78.18 | 193.9 | 0.3 | 2 | 616.7 | 65.25 | 1 |  | 75 | -16.76 | 0.07 |  | 75 | -17.43 | 36.11 |
| 76 | -21.28 | 141.8 | 98 | 0.3 | 2 | 491.7 | 80.63 | 0.6 |  | 76 | -16.76 | 0.07 |  | 76 | -17.43 | 36.09 |
| 77 | -20.64 | 89.51 | 104.1 | 0.3 | 2 | 553.2 | 67.04 | 0.6 |  | 77 | -16.76 | 0.07 |  | 77 | -17.43 | 36.01 |
| 78 | -22.91 | 182.7 | 80.15 | 0.01 | 6 | 452.2 | 13.54 | 0.6 |  | 78 | -16.76 | 0.07 |  | 78 | -17.43 | 35.99 |
| 79 | -23.26 | 0.001 | 254.4 | 0.3 | 6 | 718.9 | 17.12 | 1 |  | 79 | -16.76 | 0.07 |  | 79 | -17.43 | 36.03 |
| 80 | -22.08 | 177.3 | 132.8 | 0.3 | 2.003 | 566.1 | 82.98 | 0.6 |  | 80 | -16.76 | 0.07 |  | 80 | -17.43 | 36.01 |
| 81 | -23.04 | 103.8 | 49.22 | 0.3 | 6 | 684.2 | 28.34 | 1 |  | 81 | -16.76 | 0.07 |  | 81 | -17.43 | 36.26 |
| 82 | -22.42 | 158.9 | 136.7 | 0.3 | 2 | 496.5 | 163.4 | 1 |  | 82 | -16.76 | 0.07 |  | 82 | -17.43 | 35.97 |
| 83 | -22.21 | 116.3 | 218.6 | 0.01 | 6 | 673.1 | 0.001 | 0.6 |  | 83 | -16.76 | 0.07 |  | 83 | -17.43 | 36.06 |
| 84 | -21.17 | 100.3 | 70.59 | 0.3 | 2 | 420.4 | 110.2 | 1 |  | 84 | -16.76 | 0.07 |  | 84 | -17.43 | 35.94 |
| 85 | -20.83 | 175.5 | 58.91 | 0.3 | 2 | 423.1 | 136.3 | 0.6 |  | 85 | -16.76 | 0.07 |  | 85 | -17.43 | 35.82 |
| 86 | -21.72 | 158.5 | 122.2 | 0.3 | 2 | 395.4 | 114.7 | 0.6 |  | 86 | -16.76 | 0.07 |  | 86 | -17.43 | 35.84 |
| 87 | -20.9 | 126.8 | 69.95 | 0.3 | 2 | 402.3 | 188.6 | 0.6 |  | 87 | -16.76 | 0.07 |  | 87 | -17.43 | 35.92 |
| 88 | -20.28 | 140.9 | 35.42 | 0.2565 | 2 | 357.9 | 136.9 | 0.8223 |  | 88 | -16.76 | 0.07 |  | 88 | -17.43 | 124.8 |
| 89 | -19.99 | 126.6 | 0.001 | 0.3 | 2 | 410.5 | 156.2 | 0.6 |  | 89 | -16.76 | 0.07 |  | 89 | -17.43 | 35.8 |
| 90 | -21.71 | 177.8 | 4.796 | 0.3 | 6 | 379.4 | 188.2 | 1 |  | 90 | -16.76 | 0.07 |  | 90 | -17.43 | 35.78 |
| 91 | -20.81 | 75.41 | 14.66 | 0.3 | 6 | 368 | 161.6 | 0.6 |  | 91 | -16.76 | 0.07 |  | 91 | -17.43 | 35.75 |
| 92 | -20.05 | 80.07 | 14.9 | 0.3 | 2 | 447.6 | 192.3 | 1 |  | 92 | -16.76 | 0.07 |  | 92 | -17.43 | 35.66 |
| 93 | -26.69 | 95.23 | 0.001 | 0.01 | 2 | 401 | 235.7 | 0.6 |  | 93 | -16.76 | 0.07 |  | 93 | -17.43 | 35.64 |
| 94 | -21.55 | 69.91 | 7.326 | 0.3 | 6 | 441.7 | 136.8 | 1 |  | 94 | -16.76 | 0.07 |  | 94 | -17.43 | 35.73 |
| 95 | -21.13 | 123.1 | 39.19 | 0.3 | 2 | 470.4 | 169.8 | 1 |  | 95 | -16.76 | 0.07 |  | 95 | -17.43 | 35.43 |
| 96 | -21.09 | 144.8 | 0.001 | 0.3 | 6 | 393.9 | 95.04 | 0.6 |  | 96 | -16.76 | 0.07 |  | 96 | -17.43 | 35.7 |
| 97 | -21.05 | 54.43 | 70.33 | 0.3 | 2 | 447 | 181.7 | 1 |  | 97 | -16.76 | 0.07 |  | 97 | -17.43 | 35.68 |
| 98 | -22.44 | 83.58 | 84.03 | 0.3 | 6 | 345.1 | 141.4 | 1 |  | 98 | -16.76 | 0.07 |  | 98 | -17.43 | 35.26 |
| 99 | -19.9 | 52.28 | 14.64 | 0.3 | 2 | 502.6 | 201 | 1 |  | 99 | -16.76 | 0.07 |  | 99 | -17.43 | 35.1 |
| 100 | -20.81 | 103.2 | 0.001 | 0.3 | 6 | 528.5 | 223.1 | 1 |  | 100 | -16.76 | 0.07 |  | 100 | -17.43 | 34.94 |
| 101 | -22.49 | 73.74 | 62.97 | 0.3 | 6 | 506.1 | 235.9 | 1 |  | 101 | -16.76 | 0.07 |  | 101 | -17.43 | 34.78 |
| 102 | -21.09 | 215.7 | 40.4 | 0.3 | 2 | 351 | 115.4 | 0.6 |  | 102 | -16.76 | 0.07 |  | 102 | -17.43 | 34.62 |
| 103 | -20.12 | 152.9 | 0.001 | 0.3 | 2 | 299 | 110.5 | 0.6 |  | 103 | -16.76 | 0.07 |  | 103 | -17.43 | 34.47 |
| 104 | -20.26 | 90.63 | 0.001 | 0.3 | 2 | 325.5 | 97.3 | 0.6 |  | 104 | -16.76 | 0.07 |  | 104 | -17.43 | 34.32 |
| 105 | -20.69 | 144.2 | 38.35 | 0.3 | 2 | 314.7 | 56.18 | 0.6 |  | 105 | -16.76 | 0.07 |  | 105 | -17.43 | 34.17 |
| 106 | -26.11 | 107.7 | 0.001 | 0.01 | 6 | 251.8 | 75.07 | 0.6 |  | 106 | -16.76 | 0.07 |  | 106 | -17.43 | 34.02 |
| 107 | -20 | 191.4 | 0.001 | 0.3 | 2 | 330 | 69.43 | 1 |  | 107 | -16.76 | 0.07 |  | 107 | -17.43 | 33.87 |
| 108 | -20.04 | 222 | 0.001 | 0.3 | 2 | 286.4 | 119.1 | 0.641 |  | 108 | -16.76 | 0.07 |  | 108 | -17.43 | 33.72 |
| 109 | -21.38 | 219.5 | 49.01 | 0.3 | 2 | 284.6 | 69.86 | 0.6 |  | 109 | -16.76 | 0.07 |  | 109 | -17.43 | 33.57 |
| 110 | -22.78 | 188 | 35.64 | 0.02989 | 2 | 287.4 | 166.3 | 1 |  | 110 | -16.76 | 0.07 |  | 110 | -17.43 | 33.42 |

| **sTable7 The performance of the nine models** | | | |  |  |
| --- | --- | --- | --- | --- | --- |
| Model/Feature Selector | MAE | R2 | Pearson's r | MSE | Explained Variance Score |
| **Cross val** | | | | | |
| **XGBoost** |  |  |  |  |  |
| None | 3.464 | 0.636 | 0.798 | 19.124 | 0.636 |
| FeatureWiz | 3.519 | 0.628 | 0.792 | 19.553 | 0.628 |
| RFECV | 3.656 | 0.594 | 0.771 | 21.309 | 0.594 |
| **LASSO** |  |  |  |  |  |
| **None** | **3.260** | **0.681** | **0.826** | **16.757** | **0.681** |
| FeatureWiz | 3.360 | 0.662 | 0.814 | 17.781 | 0.662 |
| RFECV | 3.475 | 0.636 | 0.798 | 19.107 | 0.636 |
| **SVR** |  |  |  |  |  |
| None | 3.314 | 0.668 | 0.818 | 17.425 | 0.668 |
| FeatureWiz | 3.409 | 0.650 | 0.806 | 18.383 | 0.650 |
| RFECV | 3.590 | 0.610 | 0.781 | 20.480 | 0.610 |
| **Test** | | | | | |
| **XGBoost** |  |  |  |  |  |
| None | 3.559 | 0.624 | 0.791 | 19.673 | 0.624 |
| FeatureWiz | 3.654 | 0.608 | 0.780 | 20.539 | 0.608 |
| RFECV | 3.757 | 0.575 | 0.760 | 22.224 | 0.576 |
| **LASSO** |  |  |  |  |  |
| **None** | **3.429** | **0.650** | **0.806** | **18.330** | **0.650** |
| FeatureWiz | 3.531 | 0.635 | 0.797 | 19.118 | 0.635 |
| RFECV | 3.672 | 0.600 | 0.775 | 20.957 | 0.600 |
| **SVR** |  |  |  |  |  |
| None | 3.329 | 0.667 | 0.817 | 17.408 | 0.667 |
| FeatureWiz | 3.430 | 0.645 | 0.804 | 18.580 | 0.645 |
| RFECV | 3.599 | 0.612 | 0.783 | 20.314 | 0.613 |
| Abbreviation: XGBoost, eXtreme gradient boosting; LASSO, least absolute shrinkage and selection operator regression; SVR, support vector regression;RFECV, recursive feature elimination with cross-validation; MAE, Mean Absolute Error; R2,R-squared; MSE,Mean Squared Error. | | | | | |

| **sTable8 236 IDPs contributed significantly and coefficient** |  |
| --- | --- |
| IDPs | Coefficient |
| Mean ISOVF in fornix on FA skeleton | 0.63239638 |
| Volume of brain stem + 4th ventricle | 0.53700073 |
| Mean FA in superior cerebellar peduncle on FA skeleton (right) | 0.40353163 |
| Weighted-mean ICVF in tract superior longitudinal fasciculus (right) | 0.39344698 |
| Mean ICVF in body of corpus callosum on FA skeleton | 0.33811052 |
| Volume of grey matter in Putamen (left) | 0.31834075 |
| Mean ISOVF in superior corona radiata on FA skeleton (right) | 0.31815219 |
| Mean FA in superior cerebellar peduncle on FA skeleton (left) | 0.31204896 |
| Mean MO in fornix on FA skeleton | 0.30426659 |
| Mean L1 in anterior limb of internal capsule on FA skeleton (left) | 0.30242779 |
| rfMRI partial correlation matrix, dimension 25 (element 180) | 0.2868067 |
| Weighted-mean L1 in tract superior thalamic radiation (right) | 0.27858702 |
| Mean L1 in genu of corpus callosum on FA skeleton | 0.27726778 |
| Mean L1 in anterior limb of internal capsule on FA skeleton (right) | 0.26696052 |
| Mean FA in body of corpus callosum on FA skeleton | 0.25222142 |
| Volume of grey matter in IX Cerebellum (left) | 0.23943784 |
| Mean ICVF in superior cerebellar peduncle on FA skeleton (right) | 0.23474188 |
| Mean OD in posterior limb of internal capsule on FA skeleton (left) | 0.21643417 |
| Mean ICVF in corticospinal tract on FA skeleton (left) | 0.20396966 |
| Mean ICVF in superior longitudinal fasciculus on FA skeleton (right) | 0.20006725 |
| Mean ICVF in medial lemniscus on FA skeleton (left) | 0.19544162 |
| Median T2star in thalamus (left) | 0.19197079 |
| Mean MO in medial lemniscus on FA skeleton (left) | 0.19037366 |
| rfMRI partial correlation matrix, dimension 25 (element 127) | 0.19023936 |
| Volume of grey matter in Lateral Occipital Cortex inferior division (left) | 0.17983447 |
| Mean L2 in fornix cres+stria terminalis on FA skeleton (left) | 0.17602046 |
| Weighted-mean ISOVF in tract superior longitudinal fasciculus (right) | 0.17599118 |
| Volume of grey matter in Pallidum (left) | 0.17534543 |
| Volume of grey matter in Insular Cortex (left) | 0.17129106 |
| rfMRI partial correlation matrix, dimension 25 (element 125) | 0.16911254 |
| Volume of grey matter in Subcallosal Cortex (right) | 0.15530884 |
| Weighted-mean ICVF in tract parahippocampal part of cingulum (left) | 0.15521556 |
| rfMRI partial correlation matrix, dimension 25 (element 89) | 0.15469503 |
| Weighted-mean OD in tract cingulate gyrus part of cingulum (left) | 0.15394248 |
| Mean MO in cingulum hippocampus on FA skeleton (right) | 0.15391417 |
| Mean L1 in superior fronto-occipital fasciculus on FA skeleton (left) | 0.14472841 |
| rfMRI partial correlation matrix, dimension 25 (element 183) | 0.14033217 |
| Mean OD in anterior limb of internal capsule on FA skeleton (right) | 0.13788315 |
| Volume of grey matter in IX Cerebellum (right) | 0.13742089 |
| Weighted-mean ISOVF in tract inferior longitudinal fasciculus (right) | 0.13680658 |
| rfMRI partial correlation matrix, dimension 25 (element 172) | 0.13304272 |
| Volume of grey matter in VIIIb Cerebellum (vermis) | 0.1303354 |
| Volume of grey matter in Supramarginal Gyrus anterior division (right) | 0.12786858 |
| Weighted-mean L2 in tract uncinate fasciculus (left) | 0.12720508 |
| Weighted-mean L2 in tract posterior thalamic radiation (right) | 0.12387737 |
| rfMRI partial correlation matrix, dimension 25 (element 96) | 0.11594916 |
| Mean L2 in posterior limb of internal capsule on FA skeleton (right) | 0.11535801 |
| rfMRI partial correlation matrix, dimension 25 (element 156) | 0.11499651 |
| Weighted-mean OD in tract corticospinal tract (left) | 0.10809856 |
| Mean MO in anterior limb of internal capsule on FA skeleton (left) | 0.10517328 |
| Mean OD in posterior limb of internal capsule on FA skeleton (right) | 0.10376177 |
| rfMRI partial correlation matrix, dimension 25 (element 112) | 0.10066096 |
| rfMRI partial correlation matrix, dimension 25 (element 22) | 0.10033753 |
| Volume of grey matter in Crus II Cerebellum (vermis) | 0.09125002 |
| Weighted-mean L2 in tract uncinate fasciculus (right) | 0.0907286 |
| Weighted-mean L3 in tract inferior fronto-occipital fasciculus (right) | 0.08449749 |
| rfMRI partial correlation matrix, dimension 25 (element 104) | 0.07267708 |
| rfMRI partial correlation matrix, dimension 25 (element 58) | 0.07079323 |
| Mean ICVF in corticospinal tract on FA skeleton (right) | 0.06730091 |
| rfMRI partial correlation matrix, dimension 25 (element 18) | 0.06627045 |
| Weighted-mean ISOVF in tract superior longitudinal fasciculus (left) | 0.0649105 |
| rfMRI partial correlation matrix, dimension 25 (element 81) | 0.06410946 |
| rfMRI partial correlation matrix, dimension 25 (element 164) | 0.06325011 |
| rfMRI partial correlation matrix, dimension 25 (element 98) | 0.06241136 |
| Mean OD in posterior thalamic radiation on FA skeleton (right) | 0.06114301 |
| Mean ICVF in retrolenticular part of internal capsule on FA skeleton (right) | 0.06104905 |
| Mean MO in anterior limb of internal capsule on FA skeleton (right) | 0.06101344 |
| Mean FA in superior corona radiata on FA skeleton (left) | 0.06050054 |
| Volume of grey matter in VIIb Cerebellum (left) | 0.05953093 |
| rfMRI partial correlation matrix, dimension 25 (element 75) | 0.05752633 |
| Volume of grey matter in Lateral Occipital Cortex superior division (left) | 0.05501194 |
| Volume of grey matter in Inferior Temporal Gyrus temporooccipital part (left) | 0.05302457 |
| Total volume of white matter hyperintensities (from T1 and T2 FLAIR images) | 0.05267173 |
| rfMRI partial correlation matrix, dimension 25 (element 146) | 0.05110334 |
| rfMRI partial correlation matrix, dimension 25 (element 114) | 0.05010259 |
| rfMRI partial correlation matrix, dimension 25 (element 194) | 0.04987365 |
| Volume of grey matter in Angular Gyrus (left) | 0.04853918 |
| rfMRI partial correlation matrix, dimension 25 (element 202) | 0.04771877 |
| rfMRI partial correlation matrix, dimension 25 (element 1) | 0.0467831 |
| Mean L2 in fornix cres+stria terminalis on FA skeleton (right) | 0.04632566 |
| rfMRI partial correlation matrix, dimension 25 (element 97) | 0.04600262 |
| Mean L1 in superior cerebellar peduncle on FA skeleton (left) | 0.04450035 |
| rfMRI partial correlation matrix, dimension 25 (element 71) | 0.04160236 |
| Mean FA in corticospinal tract on FA skeleton (right) | 0.04153725 |
| Mean L1 in uncinate fasciculus on FA skeleton (right) | 0.03829161 |
| 90th percentile of BOLD effect (in group-defined mask) for shapes activation | 0.03748169 |
| rfMRI partial correlation matrix, dimension 25 (element 55) | 0.03732887 |
| Weighted-mean ISOVF in tract superior thalamic radiation (right) | 0.03351093 |
| Volume of grey matter in Occipital Fusiform Gyrus (left) | 0.03326568 |
| Weighted-mean ISOVF in tract uncinate fasciculus (left) | 0.03278621 |
| Weighted-mean OD in tract inferior longitudinal fasciculus (left) | 0.03068601 |
| rfMRI partial correlation matrix, dimension 25 (element 189) | 0.02858947 |
| rfMRI partial correlation matrix, dimension 25 (element 94) | 0.02755874 |
| Mean OD in cerebral peduncle on FA skeleton (right) | 0.02689543 |
| Volume of grey matter in Insular Cortex (right) | 0.02410251 |
| Volume of grey matter in Putamen (right) | 0.02351362 |
| Mean OD in retrolenticular part of internal capsule on FA skeleton (left) | 0.02235693 |
| Median T2star in thalamus (right) | 0.01985387 |
| rfMRI partial correlation matrix, dimension 25 (element 155) | 0.0191846 |
| Weighted-mean OD in tract uncinate fasciculus (left) | 0.0142717 |
| rfMRI partial correlation matrix, dimension 25 (element 122) | 0.01361218 |
| Mean L1 in superior corona radiata on FA skeleton (right) | 0.0107198 |
| rfMRI partial correlation matrix, dimension 25 (element 210) | 0.01024311 |
| Weighted-mean MO in tract superior longitudinal fasciculus (right) | -0.0146502 |
| Volume of grey matter in Middle Temporal Gyrus anterior division (left) | -0.0148576 |
| Volume of grey matter in Inferior Temporal Gyrus posterior division (right) | -0.0148607 |
| rfMRI partial correlation matrix, dimension 25 (element 56) | -0.0184822 |
| Mean L2 in cingulum cingulate gyrus on FA skeleton (left) | -0.0189058 |
| Mean OD in superior cerebellar peduncle on FA skeleton (left) | -0.0198228 |
| Mean MO in cerebral peduncle on FA skeleton (left) | -0.0206821 |
| Volume of grey matter in Lingual Gyrus (left) | -0.0217873 |
| Weighted-mean MO in tract middle cerebellar peduncle | -0.0222969 |
| rfMRI partial correlation matrix, dimension 25 (element 109) | -0.0227372 |
| Mean OD in corticospinal tract on FA skeleton (left) | -0.0248262 |
| rfMRI partial correlation matrix, dimension 25 (element 35) | -0.0253102 |
| Mean L2 in medial lemniscus on FA skeleton (right) | -0.026349 |
| Weighted-mean MO in tract posterior thalamic radiation (right) | -0.0269028 |
| rfMRI partial correlation matrix, dimension 25 (element 2) | -0.0270056 |
| Volume of grey matter in VI Cerebellum (vermis) | -0.0302406 |
| Mean MO in uncinate fasciculus on FA skeleton (right) | -0.0302816 |
| rfMRI partial correlation matrix, dimension 25 (element 64) | -0.0316925 |
| rfMRI partial correlation matrix, dimension 25 (element 60) | -0.032368 |
| Volume of grey matter in Crus I Cerebellum (right) | -0.0335441 |
| Volume of grey matter in Planum Temporale (right) | -0.0348715 |
| rfMRI partial correlation matrix, dimension 25 (element 7) | -0.0353257 |
| Mean L1 in sagittal stratum on FA skeleton (left) | -0.0355849 |
| Volume of grey matter in Frontal Orbital Cortex (right) | -0.0370673 |
| rfMRI partial correlation matrix, dimension 25 (element 48) | -0.0381027 |
| Volume of grey matter in Superior Frontal Gyrus (left) | -0.0383701 |
| Volume of grey matter in Juxtapositional Lobule Cortex (formerly Supplementary Motor Cortex) (right) | -0.0389228 |
| Weighted-mean FA in tract middle cerebellar peduncle | -0.0465128 |
| Mean ISOVF in cingulum cingulate gyrus on FA skeleton (right) | -0.0468302 |
| Volume of grey matter in Heschl's Gyrus (includes H1 and H2) (left) | -0.0469619 |
| Median T2star in amygdala (left) | -0.0481995 |
| Mean L1 in cerebral peduncle on FA skeleton (left) | -0.0491412 |
| Volume of grey matter in Planum Polare (left) | -0.050273 |
| rfMRI partial correlation matrix, dimension 25 (element 149) | -0.0505063 |
| rfMRI partial correlation matrix, dimension 25 (element 13) | -0.0543527 |
| rfMRI partial correlation matrix, dimension 25 (element 188) | -0.0556467 |
| rfMRI partial correlation matrix, dimension 25 (element 196) | -0.0571417 |
| Weighted-mean ISOVF in tract middle cerebellar peduncle | -0.0572953 |
| Volume of grey matter in Inferior Frontal Gyrus pars triangularis (left) | -0.0578196 |
| Mean ICVF in fornix cres+stria terminalis on FA skeleton (right) | -0.0587825 |
| rfMRI partial correlation matrix, dimension 25 (element 185) | -0.0587943 |
| Volume of grey matter in Planum Polare (right) | -0.0601196 |
| Volume of grey matter in X Cerebellum (right) | -0.0613511 |
| rfMRI partial correlation matrix, dimension 25 (element 135) | -0.061691 |
| rfMRI partial correlation matrix, dimension 25 (element 49) | -0.0624796 |
| Weighted-mean MO in tract superior longitudinal fasciculus (left) | -0.0627618 |
| Mean OD in tapetum on FA skeleton (right) | -0.065449 |
| Weighted-mean MO in tract forceps major | -0.0670275 |
| Volume of putamen (right) | -0.0674604 |
| Mean L1 in cerebral peduncle on FA skeleton (right) | -0.0674991 |
| Mean L2 in retrolenticular part of internal capsule on FA skeleton (right) | -0.0692981 |
| Mean ICVF in inferior cerebellar peduncle on FA skeleton (left) | -0.0702409 |
| Mean MD in retrolenticular part of internal capsule on FA skeleton (left) | -0.0715778 |
| Volume of grey matter in Precentral Gyrus (left) | -0.0733575 |
| Volume of thalamus (right) | -0.0772755 |
| rfMRI partial correlation matrix, dimension 25 (element 87) | -0.0807904 |
| Volume of grey matter in Temporal Occipital Fusiform Cortex (left) | -0.083149 |
| Weighted-mean L1 in tract acoustic radiation (left) | -0.0855802 |
| Mean OD in fornix on FA skeleton | -0.0871757 |
| Volume of grey matter in Juxtapositional Lobule Cortex (formerly Supplementary Motor Cortex) (left) | -0.0875241 |
| rfMRI partial correlation matrix, dimension 25 (element 84) | -0.0878836 |
| Volume of grey matter in I-IV Cerebellum (left) | -0.0892974 |
| Mean L2 in cingulum cingulate gyrus on FA skeleton (right) | -0.0901606 |
| Volume of grey matter in Paracingulate Gyrus (right) | -0.0917054 |
| rfMRI partial correlation matrix, dimension 25 (element 66) | -0.0933159 |
| rfMRI partial correlation matrix, dimension 25 (element 46) | -0.0954863 |
| Weighted-mean L1 in tract corticospinal tract (left) | -0.0958175 |
| Volume of grey matter in Crus I Cerebellum (vermis) | -0.0996528 |
| 90th percentile of z-statistic (in group-defined amygdala activation mask) for faces-shapes contrast | -0.1029211 |
| Volume of grey matter in Lingual Gyrus (right) | -0.1054852 |
| Volume of grey matter in Frontal Operculum Cortex (left) | -0.1055176 |
| rfMRI partial correlation matrix, dimension 25 (element 92) | -0.1145308 |
| rfMRI partial correlation matrix, dimension 25 (element 130) | -0.1146744 |
| Weighted-mean L3 in tract cingulate gyrus part of cingulum (right) | -0.1162433 |
| Weighted-mean ICVF in tract anterior thalamic radiation (left) | -0.1167197 |
| Volume of grey matter in Inferior Temporal Gyrus anterior division (right) | -0.1183565 |
| Median z-statistic (in group-defined amygdala activation mask) for faces-shapes contrast | -0.1196376 |
| Mean FA in fornix cres+stria terminalis on FA skeleton (right) | -0.1204354 |
| Volume of grey matter in X Cerebellum (left) | -0.1214291 |
| Mean ICVF in tapetum on FA skeleton (right) | -0.1271127 |
| Weighted-mean MO in tract medial lemniscus (left) | -0.1330638 |
| Weighted-mean MO in tract acoustic radiation (right) | -0.1359189 |
| Mean FA in fornix on FA skeleton | -0.1377123 |
| Volume of grey matter in Frontal Orbital Cortex (left) | -0.1398305 |
| Volume of grey matter in V Cerebellum (right) | -0.1403827 |
| rfMRI partial correlation matrix, dimension 25 (element 25) | -0.1426281 |
| 90th percentile of BOLD effect (in group-defined mask) for faces-shapes contrast | -0.1443806 |
| Mean OD in medial lemniscus on FA skeleton (right) | -0.1491877 |
| Mean MO in fornix cres+stria terminalis on FA skeleton (right) | -0.1546988 |
| Weighted-mean ICVF in tract middle cerebellar peduncle | -0.1614371 |
| Weighted-mean MO in tract parahippocampal part of cingulum (right) | -0.1663455 |
| Weighted-mean MO in tract acoustic radiation (left) | -0.1675691 |
| Mean MO in external capsule on FA skeleton (right) | -0.1699474 |
| rfMRI partial correlation matrix, dimension 25 (element 131) | -0.1705776 |
| Mean L1 in pontine crossing tract on FA skeleton | -0.1754503 |
| Volume of grey matter in Brain-Stem | -0.1759227 |
| Mean L2 in splenium of corpus callosum on FA skeleton | -0.1766506 |
| Volume of grey matter in Supramarginal Gyrus posterior division (right) | -0.1781439 |
| Weighted-mean OD in tract posterior thalamic radiation (left) | -0.181897 |
| Mean L1 in superior longitudinal fasciculus on FA skeleton (left) | -0.1836557 |
| Volume of grey matter in Crus II Cerebellum (right) | -0.1839178 |
| Mean L1 in sagittal stratum on FA skeleton (right) | -0.1882341 |
| rfMRI partial correlation matrix, dimension 25 (element 44) | -0.1917938 |
| rfMRI partial correlation matrix, dimension 25 (element 151) | -0.1925147 |
| Mean ISOVF in posterior limb of internal capsule on FA skeleton (left) | -0.1975462 |
| Mean MO in inferior cerebellar peduncle on FA skeleton (right) | -0.2018971 |
| Volume of grey matter in Frontal Operculum Cortex (right) | -0.2157617 |
| Volume of peripheral cortical grey matter (normalised for head size) | -0.2235255 |
| rfMRI partial correlation matrix, dimension 25 (element 161) | -0.225094 |
| Volume of grey matter in Heschl's Gyrus (includes H1 and H2) (right) | -0.2357587 |
| rfMRI partial correlation matrix, dimension 25 (element 103) | -0.2384892 |
| Weighted-mean MO in tract posterior thalamic radiation (left) | -0.2497549 |
| Weighted-mean OD in tract posterior thalamic radiation (right) | -0.2632587 |
| Volume of grey matter in Hippocampus (left) | -0.2649431 |
| Volume of grey matter in VI Cerebellum (left) | -0.2716381 |
| Mean L3 in retrolenticular part of internal capsule on FA skeleton (left) | -0.2763911 |
| Mean FA in middle cerebellar peduncle on FA skeleton | -0.2777566 |
| Mean L1 in middle cerebellar peduncle on FA skeleton | -0.2959536 |
| Mean ICVF in posterior thalamic radiation on FA skeleton (left) | -0.3023395 |
| Weighted-mean ICVF in tract anterior thalamic radiation (right) | -0.3132339 |
| Weighted-mean ICVF in tract forceps minor | -0.3233029 |
| Weighted-mean L3 in tract medial lemniscus (right) | -0.3411833 |
| Volume of putamen (left) | -0.3528838 |
| Weighted-mean OD in tract anterior thalamic radiation (left) | -0.3577415 |
| Median T2star in putamen (left) | -0.3770947 |
| Mean MO in fornix cres+stria terminalis on FA skeleton (left) | -0.3833091 |
| Weighted-mean ISOVF in tract forceps minor | -0.4383123 |
| Weighted-mean OD in tract anterior thalamic radiation (right) | -0.4512104 |
| Mean FA in cerebral peduncle on FA skeleton (left) | -0.466973 |
| Volume of grey matter in Ventral Striatum (left) | -0.485192 |
| Volume of thalamus (left) | -0.5469837 |
| Weighted-mean FA in tract forceps minor | -0.6239621 |
| Volume of grey matter (normalised for head size) | -1.2089862 |
| Abbreviations: IDPs, imaging-derived phenotypes |  |
| *rfMRI partial correlation matrix is a partial correlation matrix with 25 dimensionalities, that had been converted into vectors with 210 elements | |

| **sTable9 Other known liver disease or excessive alcohol intake** | |
| --- | --- |
| **Condition** | **ICD-10/definition** |
| Excessive alcohol intake | < 30 g/day for men, < 20 g/day for women |
| Alcohol abuse | F10 |
| Drug_induced_liver_disease | K71 |
| Viral_hepatitis_B_or_C_infection | B16.2, B16.9, B17.0-B17.9, B18.0-B18.9, B19.0- B19.9, B00.8; B25.1 |
| Budd_Chiari | I82 |
| Liver_abscess | K75.0, A06.4 |
| HIV | B20-B24 |
| Hemochromatosis | E83.1 |
| Wilsons_disease | E83.0 |
| Autoimmune_hepatitis | K75.4 |
| Primary_biliary_cholangitis | K74.3, K74.4 |

| **sTable10 Software and algorithms** | |  |  |
| --- | --- | --- | --- |
| Algorithms | Platform | Package | Version |
| Feature-Wiz | python | featurewiz | 0.3.2 |
| RFECV | python | scikit-learn | 1.5.1 |
| XGBoost | python | xgboost | 2.1.1 |
| Lasso | python | scikit-learn | 1.5.1 |
| SVR | python | scikit-learn | 1.5.1 |
| Bayesian Optimization | python | bayesian-optimization | 1.5.1 |
| Modal save | python | joblib | 1.4.2 |
| Baseline table | R | tableone | 0.13.2 |
| RCS figure | R | rms | 6.8-2 |
|  | R | ggplot2 | 3.5.1 |
| Abbreviation: RFECV, recursive feature elimination with cross-validation; XGBoost, eXtreme gradient boosting; LASSO, least absolute shrinkage and selection operator regression; SVR, support vector regression; RCS, restricted cubic splines. | | | |

**sTable11 GVIF Results for Variables in simultaneous model**

| **Variable** | **GVIF** | **Df** | **GVIF^(1/(2*Df))** |
| --- | --- | --- | --- |
| **PDFF** | 1.984201 | 1 | 1.408617 |
| **cT1** | 1.563328 | 1 | 1.250331 |
| **Location of assessment center** | 1.012603 | 3 | 1.00209 |
| **Age** | 1.155346 | 1 | 1.07487 |
| **sex** | 1.215879 | 1 | 1.102669 |
| **ethnic** | 1.03156 | 1 | 1.015657 |
| **Education level** | 1.066044 | 2 | 1.016117 |
| **TDI** | 1.038075 | 3 | 1.006247 |
| **Smoking status** | 1.083007 | 2 | 1.020135 |
| **Alcohol intake** | 1.182586 | 1 | 1.087468 |
| **Physical activity** | 1.047088 | 2 | 1.01157 |
| **Social connection** | 1.017699 | 1 | 1.008811 |
| **Cardiometabolic burden** | 1.655141 | 1 | 1.286523 |
| **BMI** | 1.843078 | 1 | 1.3576 |

Abbreviations: SD, standard deviation; PDFF, proton density fat fraction; cT1, iron-corrected T1 mapping; BMI, body mass index; TDI, Townsend deprivation index; SBP, systolic blood pressure; Brain-PAD, brain-Predicted Age Difference.

| **sTable12 Subgroup analyses for the association between cT1 and brain-PAD stratified by clinical threshold for PDFF (≥ 5.5% / < 5.5%)** | | | | | | | | |
| --- | --- | --- | --- | --- | --- | --- | --- | --- |
|  |  | **< 5.5%** | |  | **≥ 5.5%** | |  | ***P* for interaction** |
|  |  | **Beta (95%CI)** | ***P* value** |  | **Beta (95%CI)** | ***P* value** |  |  |
| **cT1** |  |  |  |  |  |  |  |  |
| < 800 ms |  | Reference |  |  | Reference |  |  |  |
| ≥ 800 ms |  | 1.52 (0.66, 2.37) | <0.001 |  | 0.90 (0.48, 1.31) | <0.001 |  | 0.342 |
| Continuous (log form) |  | 4.87 (3.53, 6.20) | <0.001 |  | 3.79 (1.72, 5.87) | <0.001 |  | 0.803 |
| Abbreviations: PDFF, proton density fat fraction; cT1, iron-corrected T1 mapping, Brain-PAD, brain-Predicted Age Difference. Model was adjusted for age, sex, location of assessment center, ethnic background, education level, Townsend deprivation index, smoking status, alcohol intake, physical activity, social connection, cardiometabolic burden, and BMI. | | | | | | | | |

| **sTable13 Subgroup analyses for the association between cT1 and brain-PAD stratified by median of PDFF (> 3% / ≤ 3 %)** | | | | | | | | |
| --- | --- | --- | --- | --- | --- | --- | --- | --- |
|  |  | **≤ 3%** | |  | **> 3%** | |  | ***P* for interaction** |
|  |  | **Beta (95%CI)** | ***P* value** |  | **Beta (95%CI)** | ***P* value** |  |  |
| **cT1** |  |  |  |  |  |  |  |  |
| < 800 ms |  | Reference |  |  | Reference |  |  |  |
| ≥ 800 ms |  | 2.33 (1.11, 3.56) | <0.001 |  | 1.02 (0.65, 1.40) | <0.001 |  | 0.098 |
| Continuous (log form) |  | 5.10 (3.50, 6.69) | <0.001 |  | 4.18 (2.73, 5.63) | <0.001 |  | 0.886 |
| Abbreviations: PDFF, proton density fat fraction; cT1, iron-corrected T1 mapping, Brain-PAD, brain-Predicted Age Difference. Model was adjusted for age, sex, location of assessment center, ethnic background, education level, Townsend deprivation index, smoking status, alcohol intake, physical activity, social connection, cardiometabolic burden, and BMI. | | | | | | | | |

| **sTable14 Subgroup analyses for the association of PDFF and cT1 with brain-PAD stratified by sex** | | | | | | | | |
| --- | --- | --- | --- | --- | --- | --- | --- | --- |
|  |  | **Female** | |  | **Male** | |  | ***P* for interaction** |
|  |  | **Beta (95%CI)** | ***P* value** |  | **Beta (95%CI)** | ***P* value** |  |  |
| **PDFF** |  |  |  |  |  |  |  |  |
| < 5.5% |  | Reference |  |  | Reference |  |  | <0.001 |
| ≥ 5.5% |  | 0.31 (0.04, 0.59) | 0.027 |  | 0.37 (0.11, 0.64) | 0.006 |  |  |
| Continuous (log form) | | 0.06 (-0.12, 0.25) | 0.505 |  | 0.25 (0.03, 0.46) | 0.026 |  | <0.001 |
| **cT1** |  |  |  |  |  |  |  |  |
| < 800 ms |  | Reference |  |  | Reference |  |  | <0.001 |
| ≥ 800 ms |  | 0.51 (0.00, 1.02) | 0.05 |  | 1.43 (0.95, 1.91) | <0.001 |  |  |
| Continuous (log form) | | 4.56 (3.06, 6.07) | <0.001 |  | 4.13 (2.36, 5.90) | <0.001 |  | 0.002 |
| Abbreviations: PDFF, proton density fat fraction; cT1, iron-corrected T1 mapping, Brain-PAD, brain-Predicted Age Difference.  Model was simultaneous model combining PDFF and cT1 with further adjustments for age, location of assessment center, ethnic background, education level, Townsend deprivation index, smoking status, alcohol intake, physical activity, social connection, cardiometabolic burden, and BMI. | | | | | | | | |

| **sTable15 Subgroup analyses for the association of PDFF and cT1 with brain-PAD stratified by cardiometabolic burden** | | | | | | | | |
| --- | --- | --- | --- | --- | --- | --- | --- | --- |
|  |  | **0-1 Risk factors** | |  | **≥2 Risk factors** | |  | ***P* for interaction** |
|  |  | **Beta (95%CI)** | ***P* value** |  | **Beta (95%CI)** | ***P* value** |  |  |
| **PDFF** |  |  |  |  |  |  |  |  |
| < 5.5% |  | Reference |  |  | Reference |  |  | 0.019 |
| ≥ 5.5% |  | 0.29 (-0.02, 0.60) | 0.062 |  | 0.47 (0.22, 0.72) | <0.001 |  |  |
| Continuous (log form) | | 0.22 (0.02, 0.43) | 0.032 |  | 0.21 (0.01, 0.42) | 0.041 |  | 0.015 |
| **cT1** |  |  |  |  |  |  |  |  |
| < 800 ms |  | Reference |  |  | Reference |  |  | 0.09 |
| ≥ 800 ms |  | 0.80 (0.16, 1.45) | 0.015 |  | 1.12 (0.68, 1.55) | <0.001 |  |  |
| Continuous (log form) | | 3.79 (2.29, 5.30) | <0.001 |  | 4.87 (3.09, 6.66) | <0.001 |  | 0.012 |
| Abbreviations: PDFF, proton density fat fraction; cT1, iron-corrected T1 mapping, Brain-PAD, brain-Predicted Age Difference.  Model was simultaneous model combining PDFF and cT1 with further adjustments for age, sex, location of assessment center, ethnic background, education level, Townsend deprivation index, smoking status, alcohol intake, physical activity, social connection, and BMI. | | | | | | | | |

| **sTable16 Subgroup analyses for the association of PDFF and cT1 with brain-PAD stratified by APOE ε4** | | | | | | | | |
| --- | --- | --- | --- | --- | --- | --- | --- | --- |
|  |  | **Noncarriers** | ***P* value** |  | **Carriers** | ***P* value** |  | ***P* for interaction** |
|  |  | **Beta (95%CI)** |  |  | **Beta (95%CI)** |  |  |  |
| **PDFF** |  |  |  |  |  |  |  |  |
| < 5.5% |  | Reference |  |  | Reference |  |  | 0.706 |
| ≥ 5.5% |  | 0.32 (0.10, 0.53) | 0.005 |  | 0.51 (0.10, 0.91) | 0.014 |  |  |
| Continuous (log form) |  | 0.07 (-0.1, 0.23) | 0.414 |  | 0.41 (0.11, 0.71) | 0.008 |  | 0.548 |
| **cT1** |  |  |  |  |  |  |  |  |
| < 800 ms |  | Reference |  |  | Reference |  |  | 0.392 |
| ≥ 800 ms |  | 1.07 (0.67, 1.46) | <0.001 |  | 1.47 (0.69, 2.25) | <0.001 |  |  |
| Continuous (log form) |  | 5.04 (3.70, 6.38) | <0.001 |  | 3.73 (1.39, 6.08) | 0.002 |  | 0.578 |
| Abbreviations: PDFF, proton density fat fraction; cT1, iron-corrected T1 mapping, Brain-PAD, brain-Predicted Age Difference.  Model was simultaneous model combining PDFF and cT1 with further adjustments for age, sex, location of assessment center, ethnic background, education level, Townsend deprivation index, smoking status, alcohol intake, physical activity, social connection, cardiometabolic burden, and BMI. | | | | | | | | |

| **sTable17 Subgroup analyses for the association of PDFF and cT1 with brain-PAD stratified by PRSAD** | | | | | | | | |  |  |  |
| --- | --- | --- | --- | --- | --- | --- | --- | --- | --- | --- | --- |
|  |  | **Q1** | |  | **Q2** | |  | **Q3** | |  | ***P* for interaction** |
|  |  | **Beta (95%CI)** | ***P* value** |  | **Beta (95%CI)** | ***P* value** |  | **Beta (95%CI)** | ***P* value** |  |  |
| **PDFF** |  |  |  |  |  |  |  |  |  |  |  |
| < 5.5% |  | Reference |  |  | Reference |  |  | Reference |  |  | 0.158 |
| ≥ 5.5% |  | 0.17 (-0.17, 0.51) | 0.322 |  | 0.35 (0.02, 0.68) | 0.037 |  | 0.54 (0.21, 0.87) | 0.001 |  |  |
| Continuous (log form) |  | -0.02 (-0.27, 0.23) | 0.872 |  | 0.11 (-0.14, 0.36) | 0.39 |  | 0.36 (0.12, 0.61) | 0.004 |  | 0.115 |
| **cT1** |  |  |  |  |  |  |  |  |  |  |  |
| < 800 ms |  | Reference |  |  | Reference |  |  | Reference |  |  |  |
| ≥ 800 ms |  | 1.12 (0.49, 1.74) | <0.001 |  | 1.48 (0.88, 2.07) | <0.001 |  | 0.87 (0.26, 1.49) | 0.006 |  |  |
| Continuous (log form) |  | 5.83 (3.76, 7.91) | <0.001 |  | 4.58 (2.56, 6.60) | <0.001 |  | 3.74 (1.77, 5.72) | <0.001 |  | 0.644 |
| Abbreviations: PDFF, proton density fat fraction; cT1, iron-corrected T1 mapping, Brain-PAD, brain-Predicted Age Difference, PRSAD, Alzheimer disease (AD)-related polygenic risk score.  Model was simultaneous model combining PDFF and cT1 with further adjustments for age, sex, location of assessment center, ethnic background, education level, Townsend deprivation index, smoking status, alcohol intake, physical activity, social connection, cardiometabolic burden, and BMI. | | | | | | | | |  |  |  |

| **sTable18 Subgroup analyses for the association of PDFF and cT1 and brain-PAD stratified by lifestyle** | | | | | | | | |
| --- | --- | --- | --- | --- | --- | --- | --- | --- |
|  |  | **Nonoptimal lifestyle** | |  | **Optimal lifestyle** | |  | ***P* for interaction** |
|  |  | **Beta (95%CI)** | ***P* value** |  | **Beta (95%CI)** | ***P* value** |  |  |
| **PDFF** |  |  |  |  |  |  |  |  |
| < 5.5% |  | Reference |  |  | Reference |  |  | 0.065 |
| ≥ 5.5% |  | 0.52 (0.30, 0.73) | <0.001 |  | 0.28 (-0.16, 0.72) | 0.211 |  |  |
| Continuous (log form) |  | 0.45 (0.29, 0.61) | <0.001 |  | 0.00 (-0.30, 0.30) | 0.977 |  | <0.001 |
| **cT1** |  |  |  |  |  |  |  |  |
| < 800 ms |  | Reference |  |  | Reference |  |  | 0.227 |
| ≥ 800 ms |  | 1.04 (0.65, 1.43) | <0.001 |  | 0.75 (-0.09, 1.59) | 0.08 |  |  |
| Continuous (log form) |  | 2.53 (1.22, 3.83) | <0.001 |  | 3.12 (0.82, 5.42) | 0.008 |  | 0.123 |
| Abbreviations: PDFF, proton density fat fraction; cT1, iron-corrected T1 mapping, Brain-PAD, brain-Predicted Age Difference. Model was simultaneous model combining PDFF and cT1 with further adjustments for age, sex, location of assessment center, ethnic background, education level, Townsend deprivation index, social connection, cardiometabolic burden, and BMI. | | | | | | | | |

| **sTable19 Association of brain-PAD with PDFF and cT1 when excluding liver disease or excessive alcohol intake** | | | | | | | | | | |
| --- | --- | --- | --- | --- | --- | --- | --- | --- | --- | --- |
|  | **No.** |  | **Model1** | |  | **Model2** | |  | **Model3** | |
|  |  |  | **Beta (95%CI)** | ***P* value** |  | **Beta (95%CI)** | ***P* value** |  | **Beta (95%CI)** | ***P* value** |
| **PDFF** |  |  |  |  |  |  |  |  |  |  |
| Continuous (log form) | 16278 |  | 0.63 (0.52, 0.75) | <0.001 |  | 0.31 (0.17, 0.44) | <0.001 |  | 0.04 (-0.12, 0.19) | 0.641 |
| < 5.5% | 12697 |  | Reference |  |  | Reference |  |  | Reference |  |
| ≥ 5.5% | 3581 |  | 0.84 (0.66, 1.03) | <0.001 |  | 0.42 (0.21, 0.62) | <0.001 |  | 0.26 (0.05, 0.47) | 0.015 |
| Q1 | 4947 |  | Reference |  |  | Reference |  |  | Reference |  |
| Q2 | 3761 |  | 0.19 (-0.02, 0.40) | 0.082 |  | -0.09 (-0.31, 0.12) | 0.399 |  | -0.09 (-0.31, 0.13) | 0.414 |
| Q3 | 3824 |  | 0.51 (0.30, 0.73) | <0.001 |  | 0.03 (-0.20, 0.26) | 0.797 |  | -0.03 (-0.27, 0.20) | 0.796 |
| Q4 | 3746 |  | 1.07 (0.85, 1.29) | <0.001 |  | 0.40 (0.14, 0.66) | 0.003 |  | 0.15 (-0.12, 0.42) | 0.29 |
| **cT1** |  |  |  |  |  |  |  |  |  |  |
| Continuous (log form) | 16278 |  | 6.60 (5.72, 7.62) | <0.001 |  | 5.11 (3.98, 6.24) | <0.001 |  | 4.98 (3.72, 6.24) | <0.001 |
| < 800 ms | 15508 |  | Reference |  |  | Reference |  |  | Reference |  |
| ≥ 800 ms | 770 |  | 1.70 (1.34, 2.06) | <0.001 |  | 1.26 (0.89, 1.63) | <0.001 |  | 1.14 (0.76, 1.52） | 0.015 |
| Q1 | 3998 |  | Reference |  |  | Reference |  |  | Reference |  |
| Q2 | 4134 |  | 0.33 (0.12, 0.54) | 0.003 |  | 0.28 (0.06, 0.49) | 0.011 |  | 0.27 (0.06, 0.49) | 0.013 |
| Q3 | 3993 |  | 0.59 (0.37, 0.80) | <0.001 |  | 0.45 (0.23, 0.67) | <0.001 |  | 0.42 (0.20, 0.65) | <0.001 |
| Q4 | 4153 |  | 1.18 (0.97, 1.40) | <0.001 |  | 0.87 (0.64, 1.10) | <0.001 |  | 0.80 (0.55, 1.04) | <0.001 |
| Abbreviations: PDFF, proton density fat fraction; cT1, iron-corrected T1 mapping, Brain-PAD, brain-Predicted Age Difference. Model 1 was adjusted for age, sex, location of assessment center, ethnic background, education level, Townsend deprivation index. Model 2 was adjusted for age, sex, location of assessment center, ethnic background, education level, Townsend deprivation index, smoking status, alcohol intake, physical activity, social connection, cardiometabolic burden, and BMI. Model 3 was simultaneous model in which PDFF and cT1 were modeled together on the basis of Model 2. | | | | | | | | | | |

| **sTable20 Association of brain-PAD with MASLD and cT1** | | | | | | | | | | |
| --- | --- | --- | --- | --- | --- | --- | --- | --- | --- | --- |
|  | **No.** |  | **Model1** | |  | **Model2** | |  | **Model3** | |
|  |  |  | **Beta (95%CI)** | ***P* value** |  | **Beta (95%CI)** | ***P* value** |  | **Beta (95%CI)** | ***P* value** |
| **MASLD** |  |  |  |  |  |  |  |  |  |  |
| No | 14967 |  | Reference |  |  | Reference |  |  | Reference |  |
| Yes | 3479 |  | 0.68 (0.50, 0.87) | <0.001 |  | 0.42 (0.22, 0.63) | <0.001 |  | 0.25 (0.04, 0.46) | 0.021 |
| **cT1** |  |  |  |  |  |  |  |  |  |  |
| < 800 ms | 18628 |  | Reference |  |  | Reference |  |  | Reference |  |
| ≥ 800 ms | 938 |  | 1.70 (1.37, 2.03) | <0.001 |  | 1.27 (0.93, 1.61) | <0.001 |  | 1.20 (0.81, 1.58) | <0.001 |
| Abbreviations: Brain-PAD, brain-Predicted Age Difference, MASLD, metabolic dysfunction–associated steatotic liver disease. Model 1 was adjusted for age, sex, location of assessment center, ethnic background, education level, Townsend deprivation index. Model 2 was adjusted for age, sex, location of assessment center, ethnic background, education level, Townsend deprivation index, smoking status, alcohol intake, physical activity, social connection, cardiometabolic burden, and BMI. Model 3 was simultaneous model in which MASLD and cT1 were modeled together on the basis of Model 2. | | | | | | | | | | |

| **sTable21 Association of brain-PAD with PDFF and cT1 when adjusted the waist circumference or visceral fat** | | | | | | | |
| --- | --- | --- | --- | --- | --- | --- | --- |
|  | **No.** |  | **Waist circumference** | |  | **Visceral fat** | |
|  |  |  | **Beta (95%CI)** | ***P* value** |  | **Beta (95%CI)** | ***P* value** |
| **PDFF** |  |  |  |  |  |  |  |
| Continuous (log form) | 19566 |  | 0.15 (0.01, 0.29) | 0.038 |  | 0.01 (-0.14, 0.17) | 0.861 |
| < 5.5% | 14932 |  | Reference |  |  | Reference |  |
| ≥ 5.5% | 4634 |  | 0.37 (0.18, 0.56) | <0.001 |  | 0.20 (0.00, 0.40) | 0.05 |
| Q1 | 5527 |  | Reference |  |  | Reference |  |
| Q2 | 4435 |  | -0.01 (-0.22, 0.19) | 0.914 |  | -0.04 (-0.24, 0.17) | 0.728 |
| Q3 | 4767 |  | 0.07 (-0.15, 0.29) | 0.524 |  | -0.05 (-0.27, 0.18) | 0.688 |
| Q4 | 4837 |  | 0.34 (0.09, 0.59) | 0.009 |  | 0.11 (-0.16, 0.37) | 0.43 |
| **cT1** |  |  |  |  |  |  |  |
| Continuous (log form) | 19566 |  | 4.46 (3.31, 5.61) | <0.001 |  | 4.27 (3.12, 5.42) | <0.001 |
| < 800 ms | 18628 |  | Reference |  |  | Reference |  |
| ≥ 800 ms | 938 |  | 1.07 (0.72, 1.42) | <0.001 |  | 0.99 (0.64, 1.34) | <0.001 |
| Q1 | 5028 |  | Reference |  |  | Reference |  |
| Q2 | 4947 |  | 0.24 (0.05, 0.44) | 0.015 |  | 0.24 (0.04, 0.44) | 0.017 |
| Q3 | 4716 |  | 0.34 (0.14, 0.55) | 0.001 |  | 0.33 (0.13, 0.54) | 0.002 |
| Q4 | 4875 |  | 0.75 (0.52, 0.98) | <0.001 |  | 0.70 (0.47, 0.93) | <0.001 |
| Abbreviations: PDFF, proton density fat fraction; cT1, iron-corrected T1 mapping, Brain-PAD, brain-Predicted Age Difference. Model was simultaneous model combining PDFF and cT1 with further adjustments for age, sex, location of assessment center, ethnic background, education level, Townsend deprivation index, smoking status, alcohol intake, physical activity, social connection, cardiometabolic burden, BMI, and waist circumference or visceral fat. | | | | | | | |

| **sTable22 Subgroup analyses for the association of PDFF and cT1 and brain-PAD stratified by age group** | | | | | | | | |
| --- | --- | --- | --- | --- | --- | --- | --- | --- |
|  |  | **≤ 60 years** | |  | **> 60 years** | |  | ***P* for interaction** |
|  |  | **Beta (95%CI)** | ***P* value** |  | **Beta (95%CI)** | ***P* value** |  |  |
| **PDFF** |  |  |  |  |  |  |  |  |
| < 5.5% |  | Reference |  |  | Reference |  |  |  |
| ≥ 5.5% |  | 0.58 (0.27, 0.89) | <0.001 |  | 0.36 (0.12, 0.60) | 0.003 |  | 0.346 |
| Continuous (log form) |  | 0.32 (0.09, 0.54) | 0.006 |  | 0.19 (0.01, 0.37) | 0.042 |  | 0.662 |
| **cT1** |  |  |  |  |  |  |  |  |
| < 800 ms |  | Reference |  |  | Reference |  |  |  |
| ≥ 800 ms |  | 1.02 (0.49, 1.55) | <0.001 |  | 1.10 (0.64, 1.55) | <0.001 |  | 0.751 |
| Continuous (log form) |  | 3.58 (1.79, 5.36) | <0.001 |  | 4.20 (2.70, 5.70) | <0.001 |  | 0.57 |
| Abbreviations: PDFF, proton density fat fraction; cT1, iron-corrected T1 mapping, Brain-PAD, brain-Predicted Age Difference. Model was simultaneous model combining PDFF and cT1 with further adjustments for age, sex, location of assessment center, ethnic background, education level, Townsend deprivation index, smoking status, alcohol intake, physical activity, social connection, cardiometabolic burden, and BMI. | | | | | | | | |

| **sTable23 Subgroup analyses for the association of PDFF and cT1 and white matter hyperintensity stratified by lifestyle** | | | | | | | | |
| --- | --- | --- | --- | --- | --- | --- | --- | --- |
|  |  | **Nonoptimal lifestyle** | |  | **Optimal lifestyle** | |  | ***P* for interaction** |
|  |  | **Beta (95%CI)** | ***P* value** |  | **Beta (95%CI)** | ***P* value** |  |  |
| **PDFF** |  |  |  |  |  |  |  |  |
| < 5.5% |  | Reference |  |  | Reference |  |  | 0.200 |
| ≥ 5.5% |  | 0.05 (0.01, 0.08) | <0.010 |  | 0.09 (0.02, 0.17) | 0.020 |  |  |
| Continuous (log form) |  | 0.05 (0.03, 0.08) | <0.001 |  | 0.08(0.03, 0.13) | 0.004 |  | <0.185 |
| **cT1** |  |  |  |  |  |  |  |  |
| < 800 ms |  | Reference |  |  | Reference |  |  | 0.180 |
| ≥ 800 ms |  | 0.09 (0.02, 0.15) | 0.010 |  | -0.07(-0.22, 0.08) | 0.350 |  |  |
| Continuous (log form) |  | 0.31(0.10, 0.52) | 0.004 |  | -0.06(-0.46, 0.34) | 0.770 |  | 0.51 |
| Abbreviations: PDFF, proton density fat fraction; cT1, iron-corrected T1 mapping, Brain-PAD, brain-Predicted Age Difference. Model was simultaneous model combining PDFF and cT1 with further adjustments for age, sex, location of assessment center, ethnic background, education level, Townsend deprivation index, social connection, cardiometabolic burden, and BMI. | | | | | | | | |

| **sTable24 Subgroup analyses for the association of PDFF and cT1 and brain-PAD stratified by lifestyle after excluding participants with hemochromatosis** | | | | | | | | |
| --- | --- | --- | --- | --- | --- | --- | --- | --- |
|  |  | **Nonoptimal lifestyle** | |  | **Optimal lifestyle** | |  | ***P* for interaction** |
|  |  | **Beta (95%CI)** | ***P* value** |  | **Beta (95%CI)** | ***P* value** |  |  |
| **PDFF** |  |  |  |  |  |  |  |  |
| < 5.5% |  | Reference |  |  | Reference |  |  | 0.071 |
| ≥ 5.5% |  | 0.51 (0.29, 0.72) | <0.001 |  | 0.28 (-0.16, 0.72) | 0.211 |  |  |
| Continuous (log form) |  | 0.43 (0.27, 0.59) | <0.001 |  | 0.01 (-0.29, 0.31) | 0.94 |  | <0.001 |
| **cT1** |  |  |  |  |  |  |  |  |
| < 800 ms |  | Reference |  |  | Reference |  |  | 0.225 |
| ≥ 800 ms |  | 1.05 (0.66, 1.44) | <0.001 |  | 0.75 (-0.09, 1.59) | 0.08 |  |  |
| Continuous (log form) |  | 2.61 (1.31, 3.92) | <0.001 |  | 3.10 (0.80, 5.40) | 0.010 |  | 0.120 |
| Abbreviations: PDFF, proton density fat fraction; cT1, iron-corrected T1 mapping, Brain-PAD, brain-Predicted Age Difference. Model was simultaneous model combining PDFF and cT1 with further adjustments for age, sex, location of assessment center, ethnic background, education level, Townsend deprivation index, social connection, cardiometabolic burden, and BMI. | | | | | | | | |
